# Sedimentary Ancient DNA Tracks Multi‐Kingdom Ecosystem Reorganizations Following Sequential Human Land Use at Crawford Lake

**DOI:** 10.1111/mec.70529

**Published:** 2026-09-08

**Authors:** Tyler J. Murchie, Matthew V. Emery, Scott L. Cocker, Libby Natola, Melanie Kuch, Linda Y. Rutledge, Francine McCarthy, Michael F. J. Pisaric, Hendrik N. Poinar

**Affiliations:** ^1^ Biodiversity Genomics Hakai Institute Heriot Bay British Columbia Canada; ^2^ Department of Anthropology McMaster University Hamilton Ontario Canada; ^3^ Department of Anthropology Binghamton University Binghamton New York USA; ^4^ School of Human Evolution and Social Change Arizona State University Tempe Arizona USA; ^5^ Department of Earth Sciences Brock University St. Catharines Ontario Canada; ^6^ Department of Geological Sciences Stockholm University Stockholm Sweden; ^7^ Centre for Palaeogenetics Stockholm Sweden; ^8^ Department of Forest and Conservation Sciences University of British Columbia Vancouver British Columbia Canada; ^9^ Department of Geography and Tourism Studies Brock University St. Catharines Ontario Canada; ^10^ Department of Biochemistry and Biology McMaster University Hamilton Ontario Canada

**Keywords:** Anthropocene, Canada goose, eastern woodlands, eutrophication, horticulture, lake ecology, longhouse people, maize, sedimentary ancient DNA (sedaDNA), three sisters agriculture

## Abstract

Crawford Lake has an exceptional stratigraphic record that began recording biannual (varved) sedimentation in the lake basin ~750 years ago, preserving evidence of shifting cultural zones and agricultural practices, from Late Woodland Period Indigenous agriculturalists to the impacts of industrialization during the late 19th century. It was selected as the candidate site for the proposed ‘Anthropocene’ epoch in 2023—a proposal ultimately rejected in 2024—but the lake's significance extends beyond a formal stratigraphic boundary. Its sediments preserve a long record of human‐ecosystem entanglement that captures the cumulative, reverberating nature of local human impacts and global change. While many proxies have been studied at the site, the lake's sedimentary ancient DNA (sedaDNA) record has yet to be investigated. Here, we report on sedaDNA preserved at Crawford Lake over the last ~1300 years. Sedentism and agriculture clearly impacted the entire lake ecosystem, with corresponding shifts observable in the sedaDNA of plants, animals, algae, fungi and bacteria. Canada goose ( *Branta canadensis* ) roosting on the lake—likely drawn by foraging opportunities in fields cleared for Three/Four Sisters agriculture and sedentism—contributed to repeated eutrophications and algal blooms that permanently shifted the lake's ecological structure. Subsequent impacts during the Euro‐Canadian zone furthered anthropogenic succession, although local impacts have been minimal since closure of the sawmill in 1900 ce, allowing for sensitivity to global change. Beyond the molecular ecological history of the lake, we also evaluate the effectiveness of an Arctic/Subarctic bait‐set for palaeoecological reconstructions of the Eastern Woodlands, and the preservation of lake sedaDNA.

## 1 Introduction

Crawford Lake is a small (2.4 ha), deep (~24 m) meromictic lake (where the water column does not mix to the lakebed) in a dolomitic karst basin on the Niagara Escarpment, ~65 km west of Toronto, Ontario, Canada. The site has been studied extensively for its exceptionally preserved palaeoenvironmental and cultural record. The lake occupies a karstic sinkhole that was exposed and likely modified by ice‐marginal meltwater flow ~15,000 cal BP near the end of the Pleistocene during retreat of the Laurentide ice sheet (Karrow 1987). Varved sediments, composed of alternating summer calcite (white) and fall/winter organic laminae (black), have accumulated at times of human impact on the lake beginning in the late 13th century, creating a sub‐annually resolvable stratigraphic record (Dickman 1979, 1985; Llew‐Williams et al. 2024). The stratification of Crawford Lake's water column, reinforced by density gradients and groundwater influx, inhibits bioturbation and fosters the minimally disturbed accumulation of sedimentary sequences with well‐preserved organic matter (McCarthy et al. 2023). This stratigraphic record has enabled high‐resolution investigations into the ecological history of Indigenous Three/Four Sisters agriculture in the area from ~1250 to 1550 ce and Euro‐Canadian logging, lumber milling and farming in the 19th century (McAndrews and Turton 2007). These activities led to successive and varied levels of cultural eutrophication of the lake: the process wherein direct and indirectly linked human activities such as agriculture and deforestation cause an over‐enrichment of nutrients in a lake, leading to algal blooms, oxygen depletion, decreased biodiversity and decreased water quality (Ekdahl et al. 2004).

The exceptional paleolimnological record together with repeated instances of significant human impact and stewardship made it the candidate site for the Global boundary Stratotype Section and Point (GSSP), or ‘golden spike’, for the proposed ‘Anthropocene’ epoch, identified at the contact between the calcitic and organic laminae deposited in 1952 ce and marked by the rapid increase in radionuclides associated with detonation of the first hydrogen bomb (McCarthy, Patterson, et al. 2025; Waters et al. 2024a, 2024b). On March 5, 2024, the Subcommission on Quaternary Stratigraphy (SQS) announced that its ballot on formalizing the ‘Anthropocene’ as a new epoch failed to reach the required 60% supermajority (12 votes against, four in favour, two abstentions) and was therefore rejected what would have signified an end to the Holocene Epoch (characterized by much more stable conditions than the preceding Pleistocene Epoch) as famously posited by Paul Crutzen (Crutzen and Stoermer 2000). Debate over the validity of the ‘Anthropocene’ and the criteria by which epochs are defined are likely to continue (Skelton and Noone 2025) and the Anthropocene Working Group hopes that the GSSP proposal will be revisited, including that for a Crawfordian age that was not considered by the SQS (McCarthy, Head, et al. 2025). Nonetheless, the site records an exceptional history of humans as agents of ecological and geological change over the past millennium, with many opportunities for new methods to leverage those sub‐annual records for high‐resolution palaeoecological reconstructions beyond debates regarding a formalized ‘Anthropocene’.

Palynological analyses first revealed the presence of maize ( *Zea mays* ) pollen at the site (Boyko 1973), with additional lake core analyses and archaeological excavations identifying beans (*Phaseolus*), sunflower (*Helianthus*), squash (*Cucurbita*), purslane (*Portulaca*), cuitlacochi (maize smut, *Ustilago maydis* ) and a wide variety of faunal remains indicating Indigenous agricultural activity (Boyko‐Diakonow 1979; Boyko 1973; Ekdahl et al. 2004; Finlayson and Byrne 1975; McAndrews and Boyko‐Diakonow 1989) with AMS radiocarbon ages of charred maize kernels from excavated hearths recording two major intervals of agricultural settlement, in the mid‐14th and early 15th centuries, consistent with the findings of Ekdahl et al. (2004) and McAndrews and Turton (2007, 2010) from the palynological record. Several studies have identified a third phase of agricultural settlement just prior to permanent site abandonment, dated to the 16th century (Finlayson et al. 2026; Llew‐Williams et al. 2024). Multiproxy studies using macrofossils, charcoal, diatoms, chrysophyte cysts and scales, algal palynomorphs, micro‐XRF and stable isotopes (among other methods) have demonstrated the lake's sensitivity to land‐use change, logging, industrial emissions and atmospheric deposition over the past millennium (Ekdahl et al. 2004; Gushulak et al. 2022; Llew‐Williams et al. 2024; McCarthy, Patterson, et al. 2025; Moraal et al. 2025). The sedimentary record includes clear signals of both localized human impact, such as Indigenous horticulture and Euro‐Canadian farming and lumbering and globally distributed geochemical markers of an Earth system altered by the Great Acceleration (Steffen et al. 2015). This includes radionuclide fallout from mid‐20th century thermonuclear weapons testing, which has been used to propose 1952 ce as the base of the ‘Anthropocene’ series (McCarthy, Patterson, et al. 2025).

While these investigations have illuminated many of the abiotic and microfossil impacts of human‐environment interactions at Crawford Lake, emerging molecular techniques allow for a deeper exploration of the lake's ecological past through metagenomics and targeted enrichment. Sedimentary ancient DNA (sedaDNA) analysis offers the potential to directly detect a broad spectrum of past organisms, including plants, animals, fungi and microbes through shed environmental DNA (eDNA) preserved in sediment even in the absence of visible remains (Capo et al. 2021; Garcés‐Pastor et al. 2023; Giguet‐Covex et al. 2023; Heintzman et al. 2023; Murchie, Giguet‐Covex, et al. 2023). This method complements, and in some cases surpasses, conventional proxies in taxonomic breadth and ecological resolution (Capo et al. 2021; Giguet‐Covex et al. 2014; Graham et al. 2016; Grealy et al. 2015; Heintzman et al. 2023; Kjær et al. 2022; Ledger et al. 2025; Lejzerowicz et al. 2013; Monteath et al. 2023; Murchie et al. 2026; Murchie, Monteath, et al. 2021; Parducci et al. 2017; Pedersen et al. 2015; Wang et al. 2021; Willerslev et al. 2014).

**FIGURE 1:**
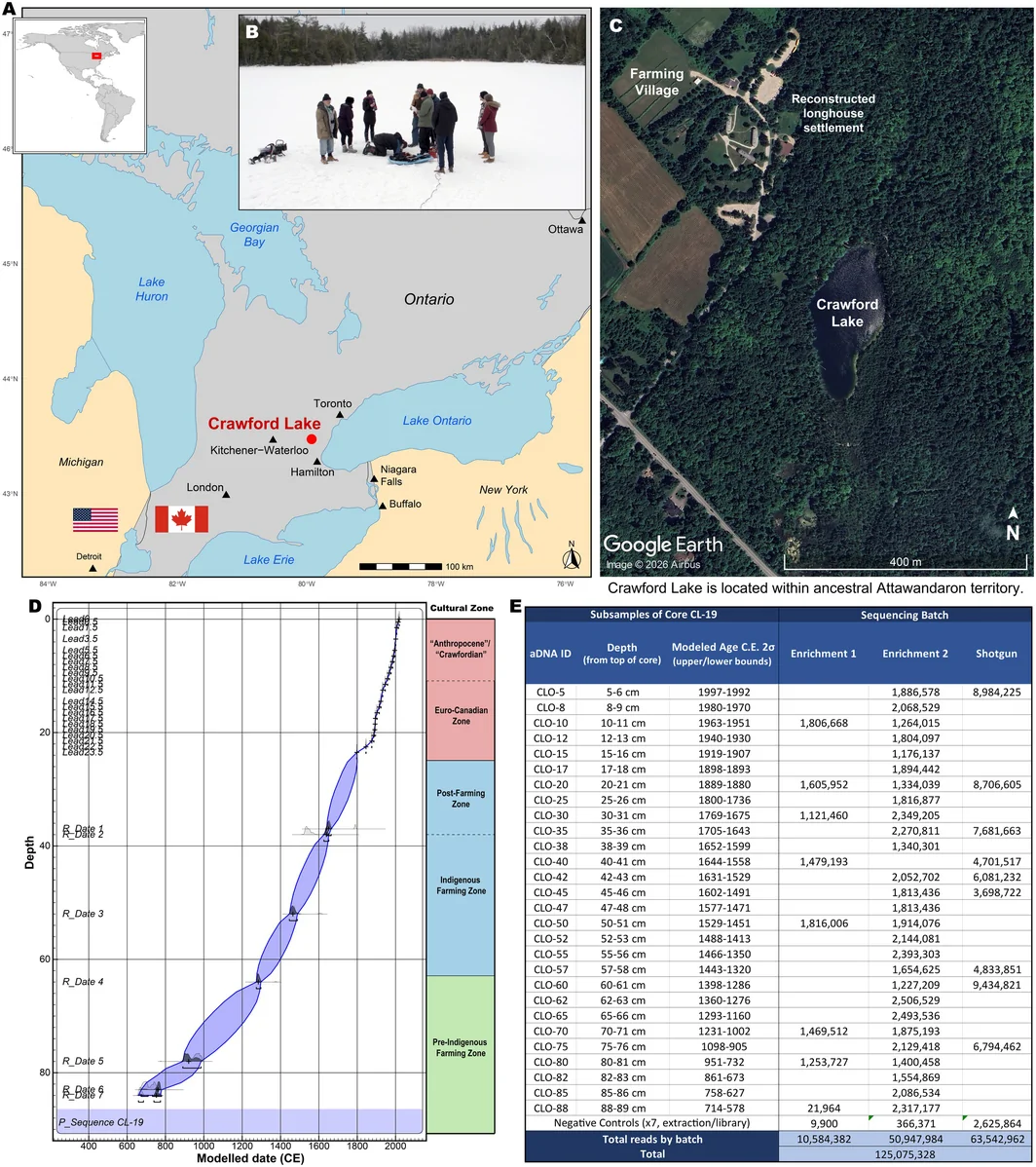
Location of Crawford Lake and associated sedaDNA samples. (A) Southern Ontario. (B) Photograph of core CL‐19 recovery in Winter 2019. (C) Google Earth (Airbus 2026) satellite image of Crawford Lake ~0.5 km from the ancient farming village (43°28′6.15″N 79°56′55.97″W). Imagery date 7/11/2025. (D) Age‐depth model for core CL‐19. Cultural zones based on Ekdahl et al. (2004) with refinement from our metagenomic CONISS analysis with a broken‐stick model (Bennett 1996; Grimm 1987) (see Figures S34–S37 and Table S7). (E) Subsamples processed for sedaDNA with age estimates and sequencing depths.

Here, we explored ~1 m of a gravity surface core recovered from Crawford Lake in 2019 with 28 subsamples of sediment processed for sedaDNA using a combination of shotgun sequencing and targeted capture enrichment (Figure 1). We used the PalaeoChip Arctic v1.0 bait‐set to capture mitochondrial genomes and chloroplast genes from Holarctic flora and fauna (Murchie 2021; Murchie et al. 2022, 2026; Murchie, Kuch, et al. 2021; Murchie, Monteath, et al. 2021). The bait‐set was initially designed for Pleistocene and early Holocene‐aged sites and intentionally excluded agricultural contaminants common in plasticware and reagents. This targeting panel was used here to take advantage of the cross‐capture potential of hybridization enrichment with the foreknowledge that some agricultural plants would likely be missed in this initial investigation. Despite this limitation, we were able to recover a diverse metagenomic spectrum of plant and animal sedaDNA that clearly mark transitions from an onset of Indigenous horticulture, site abandonment and Euro‐Canadian farming. We see a significant increase in grass (Poaceae) and Canada goose ( *Branta canadensis* ) sedaDNA during the Indigenous farming phase, with corresponding transitions in microbial and algal communities associated with previously identified periods of cultural eutrophication (Ekdahl et al. 2004, 2007). This sedaDNA record helps underscore the long history of human interaction with and impacts on Crawford Lake, as well as the utility of sedimentary metagenomics for complementing traditional palaeoecological methods. We also investigated the on‐target capture utility of an Arctic bait‐set for a temperate deciduous forest site, finding that while the method was effective, future analyses would benefit from a more regionally specific RNA bait panel. Furthermore, we evaluate the significant deamination signal detected in these sedaDNA records, which clearly demonstrates that DNA damage is more impacted by local taphonomic conditions rather than age alone (Jia et al. 2022; Kistler et al. 2017; Schwarz et al. 2009; Smith et al. 2003).

## 2 The History of Crawford Lake

Southern Ontario has a rich history of Indigenous Peoples with archaeological records dating back to the retreat of the Laurentide ice sheet. People have lived in the area since at least ~11,000 years ago, which has been archaeologically categorized into three main periods (Ferris and Spence 1995; Willey and Phillips 1958; Wright 1981). The Palaeo‐American Period (~11,000–9500 years ago, also known as the Lithic Stage) is defined by small, mobile, hunter‐gatherer bands of post‐glacial peoples as they rapidly spread throughout the Americas. The Archaic Period (9500–3000 years ago) saw an increase in trade networks, population density and lithic/tool diversity; and the Woodland Period (3000 years ago to contact with Europeans [~1600 ce]) records the introduction of pottery, increased sedentism, significant social complexity and an increased reliance on horticulture (i.e., maize, beans and squash (Ferris and Spence 1995; Mt.Pleasant 2016; Ngapo et al. 2021; Scarry 2008; Willey and Phillips 1958; Wright 1981)).

Most of the lake sediment research at Crawford Lake has focused on the upper deposits that record the last ~1000 years. Longhouse People (the preferred name of the Crawford Lake Indigenous Council, otherwise referred to in the literature as Iroquoian language group people or similar terms) introduced Three/Four Sisters Agriculture (maize, beans, squash and sunflowers) to the southern Great Lakes region during the Late Woodland Period (~1000–1650 ce) (Bamann et al. 1992), with evidence of a dozen longhouse villages in the region around Crawford Lake and the latest in the northwestern part of the Crawford Lake catchment (Finlayson 1998b). Archaeological excavations at the Crawford Lake Village site have revealed evidence for at least two (potentially three) settlement phases dating to the mid‐14th and 15th‐16th centuries, marked by longhouses, storage pits and refuse middens indicative of maize horticulture, supplemented by beans, squash and sunflowers, among other wild plants (Finlayson 1998b; Finlayson et al. 2026; Finlayson and Byrne 1975). Palynological data support this chronology, showing increased nonarboreal (herbaceous) pollen including that of *Zea mays* (corn/maize) and other cultigens and spores of fungal pathogens (McAndrews and Turton 2010), and other anthropogenic indicators during these intervals (McAndrews and Turton 2007; McCarthy et al. 2023). Based on village size and the number of longhouses, it is estimated that the population at the village was ~200 individuals during the first settlement phase and ~400 individuals during the second (Finlayson 1998b). The village size of the potential third occupation is unknown but thought to be minor (Finlayson et al. 2026). There is evidence for as many as seven villages within 3 km of the lake, with a total population in the area estimated up to ~3000 people (Finlayson 1998b). Investigations of the lake's geochemistry, diatom assemblages and other microbes found evidence for eutrophication of the lake that occurred during this farming period, which altered the lake's ecosystem and created the varving that persists today (Ekdahl et al. 2004; Turton and McAndrews 2006).

After abandonment of the site in the ~16th century (Ekdahl et al. 2004), which may correspond with a large flood (Moraal 2024), the region entered a period of ecological change and succession through the Little Ice Age (ca. 1570–1850 ce with prominent cold intervals at ca. 1650, 1770 and 1850 ce) (Matthews and Briffa 2005), although lake conditions prior to human impacts were never reestablished (Ekdahl et al. 2004, 2007; McCarthy et al. 2018). Euro‐Canadian settlement in the 19th century saw renewed impacts from logging and lumber milling in the Crawford Lake catchment and agriculture in the region (McCarthy et al. 2023). This led to another phase of cultural eutrophication and algal blooms (Ekdahl et al. 2004; Gushulak et al. 2022; McCarthy et al. 2018, 2023; McCarthy, Patterson, et al. 2025). The ensuing decades witnessed the progressive transformation of southern Ontario's landscapes under the pressures of industrialization and population growth. Within Crawford Lake itself, these shifts are recorded in a range of geochemical and microfossil proxies, culminating in the distinct suite of stratigraphic markers characteristic of the ‘Anthropocene’ GSSP proposal, including: plutonium isotopes, synthetic compounds, spheroidal carbonaceous particles and isotopic signatures of fossil fuel combustion (McCarthy, Patterson, et al. 2025; Moraal et al. 2025). These markers are concentrated within the upper 20 cm, with 1952 ce occurring ~15 cm in core CL‐19.

Much of the ancient biotic signal reconstructed in previous investigations of the site has either focused on remains that can be visually identified (macro‐ or microscopically) or have targeted the site's geochemistry (Boyko‐Diakonow 1979; Boyko 1973; Clark and Royall 1995; Ekdahl et al. 2004, 2007; Finlayson 1998a; Finlayson and Byrne 1975; Gushulak et al. 2022; Llew‐Williams et al. 2024; McAndrews and Boyko‐Diakonow 1989; McAndrews and Turton 2007, 2010; McCarthy et al. 2023; McCarthy, Patterson, et al. 2025; Moraal et al. 2025; Turton and McAndrews 2006; Waters et al. 2024b). The biomolecular preservation of Crawford Lake has yet to be investigated, with ancient environmental DNA having the potential to recover high resolution biotic signals from all kingdoms of life simultaneously to understand how the lake system has changed in an ecologically holistic sense (Murchie, Long, et al. 2023). SedaDNA also has the potential to add phylogenetic resolution by reassembling genomes of various species from fragments of DNA preserved in sediment that can be analysed in relation to genomes assembled from macrofossils and historic/contemporary specimens, allowing for the study of dispersal or trade patterns/routes for key agricultural taxa as well as genes under selective pressure from farming (Murchie et al. 2022; Murchie, Giguet‐Covex, et al. 2023). We focused our efforts here on the Indigenous farming period ca. 1300–1600 (Ekdahl et al. 2004; Finlayson and Byrne 1975) to investigate the sedaDNA signals of sedentism and horticulture at the site, and to determine how well our Arctic/Subarctic focused baits functioned for enriching Late Woodland Period associated taxa.

## 3 Materials and Methods

### 3.1 Field Work

The Crawford Lake Indigenous Council was informed of our planned research, and prior to sampling it was agreed upon that we would study sedaDNA preserved in the lake core to study the local palaeoecological record. It was also agreed upon that should any endogenous human DNA found in the lake be detected, it would be removed prior to any analyses. The human DNA described in this paper stemmed from contaminating DNA sources and not the original inhabitants living around the lake.

Field coring for this study was conducted on February 19, 2019, using a UWITEC gravity corer deployed through a hole augured in the ice over the deep meromictic basin (22.1 m) of Crawford Lake. A ~90 cm‐long gravity core (CL‐19) was successfully extracted from the basin and immediately subsampled in the field at 1 cm intervals using a UWITEC extruder to avoid the loss of sediment through degassing. Subsampling was performed using contamination‐aware protocols suitable for ancient DNA (aDNA) research (e.g., sterile sampling tools, removing only the interior portion of sediment from the core, frequent changing of gloves), which allowed us to simultaneously collect samples for sedaDNA, pollen, macrofossil and geochemical analyses. SedaDNA subsamples were collected with single‐use sterile plastic scoopulas deposited in sterile collection tubes. Human DNA was not a target of the present study, and as such, initial subsampling was not conducted within a dedicated clean room facility. All sedaDNA subsamples were promptly frozen and transported to the McMaster Ancient DNA Centre (McMaster University, Hamilton, Ontario), where they were archived for biomolecular processing.

### 3.2 Ancient DNA Wet Lab

Ancient DNA wet laboratory processing was conducted in dedicated clean rooms at the McMaster Ancient DNA Centre. The facility has a physically isolated sedimentary subsampling and extraction laboratory (separate buildings from the standard macrofossil [i.e., bone] processing area), along with a physically separate lab space for reagent preparation. The post‐indexing workspace (also used for capture enrichment) is in another physically separated lab, along with a high copy post‐PCR workspace located in a communal lab space in another building. The centre has a unidirectional workflow standard operating procedure (SOP) progressing from low‐ to high‐copy facilities, which is intended to reduce the chances of cross‐contamination between and within projects. Intra‐lab workspaces are physically separated, including air pressure gradients between rooms to reduce airborne DNA contamination, along with clean room specific SOPs. Prior to all phases of laboratory work, dead air hoods and workspaces were cleaned using a 6% solution of sodium hypochlorite (commercial bleach) followed by a wash with Nanopure purified water (Barnstead) and > 30 min of UV irradiation at > 100 mJ/cm^2^. Full‐body Tyvek suits and masks were worn at all times when in the cleanrooms, and gloves were cleaned and changed frequently during sample processing.

#### 3.2.1 Wet‐Lab Subsampling

Subsamples were processed in two batches. In 2020, we processed nine samples at 10 cm intervals along the core as a preliminary screen to assess sedaDNA preservation. After initial results suggested that there was an ecologically significant sedaDNA signal from the farming period samples (~40–60 cm), a second higher resolution batch was processed in 2022/2023 with 29 subsamples intended for both shotgun sequencing and capture enrichment (Figure 1). Both batches were processed identically (except where noted during enrichment) with negative controls processed alongside samples throughout (for a total of five negative controls). Wet‐lab subsampling used sterilized, single use plasticware, with UV‐C sterilized weigh boats.

#### 3.2.2 Extraction, Library Preparation, Enrichment and Sequencing

Sediment DNA was extracted using established sedaDNA protocols with overnight proteinase K digestion, bead beating, inhibitor removal by cold spin and silica‐column purification (Dabney, Knapp, et al. 2013; Karpinski et al. 2016; Murchie, Kuch, et al. 2021). Double‐stranded libraries were prepared without UDG treatment to retain characteristic ancient DNA damage patterns (Meyer and Kircher 2010) and indexed using dual‐unique barcodes (Kircher et al. 2012). Indexed libraries were plant/animal target enriched using the PalaeoChip Arctic v1.0 RNA bait set (Murchie, Kuch, et al. 2021) using the myBaits v4.1 and v5 protocols (Batch 1 and 2) before equimolar pooling, size selection and Illumina paired‐end sequencing. Full details on extraction chemistry, library preparation, enrichment conditions, bait‐panel scope and limitations, sequencing parameters and other associated metagenomic workflows are provided in the Supporting Information, Figures S1–S46, Tables S1–S11 and Datasets S1–, S4.

### 3.3 Bioinformatics

#### 3.3.1 Metagenomics

##### 3.3.1.1 On‐Target Taxa, Plants and Animals

The targeted capture metagenomics followed a 3‐stage approach. First, mapping all filtered reads to a comprehensive set of organelle reference genomes from PalaeoChip taxa to remove prokaryotes, nuclear DNA, and any other sequences with poor reference coverage. Then, after deduplicating the map‐filtered reads, running those through *BLASTn* to retrieve the top 500 alignments per read against a locally stored 2020 copy of the National Centre for Biotechnology Information GenBank Nucleotide Database (NCBI‐NT) (NCBI Resource Coordinators 2018). A large ‘top alignments’ value was used to mitigate the problems of database bias toward organisms of commercial/scientific interest, and the problems of *BLASTn* top sequence recovery limitations (Shah et al. 2019). Finally, the map‐filtered *FASTA* and *BLASTn* files were taxonomically binned using *MEGAN7* (v7.0.9‐beta) (Huson et al. 2007, 2016) to classify the reads with a strict set of LCA parameters. Additional details for this 3‐stage approach are included in the supplementary online materials (see also Figures S1–S6 and S42–S44).

##### 3.3.1.2 Off‐Target Taxa

A secondary set of metagenomic classifications was also carried out for bacteria/archaea, algae and fungi. In this set, *FASTQ* files were again trimmed and merged with *leeHom* and converted to filtered *BAM* files, which were then converted to *FASTA* format with a minimum length of 24 bp, filtered for any lingering similarity to sequencing adapters, and string deduplicated with the NGSXRemoveDuplicates module of *NGSeXplore* (https://github.com/ktmeaton/NGSeXplore). These filtered *FASTA* files were queried with *MegaBLAST* (Altschul et al. 1990) using a July 2020 local copy of *NCBI‐NT* set to return the top 50 alignments (unique accession hits) per read with *e*‐values < 1.0E‐8 (flags: ‐num_alignments 50 ‐max_hsps 1 ‐evalue 0.00001). *MegaBLAST* was used in this case over *BLASTn* because the speed of the algorithm is too slow for a dataset of this size that has not been filtered to a set of curated targets. The outputs were passed to *MEGAN7* where the *BLAST* results were filtered through a lowest common ancestor (LCA) algorithm using the following parameters: MinScore = 50.0; MaxExpected = 1.0E‐5; MinPercentIdentity = 95.0; TopPercent = 10.0; MinSupport = 3; LCA = naïve; MinPercentReadToCover = 90; mode = BlastN.

#### 3.3.2 Single‐Target Genome Assembly

Single‐target genome assemblies were generated for *Branta* mitochondrial DNA and select plant chloroplast loci using an ancient‐DNA reference‐mapping workflow (Murchie et al. 2022, 2026; Murchie, Monteath, et al. 2021). Reads were trimmed, merged, competitively mapped to other plant/animal references, deduplicated, filtered for mapping quality and fragment length and manually curated to reduce the influence of non‐specific read stacking, spurious indels and low‐confidence polymorphisms. Consensus sequences meeting coverage and completeness thresholds were aligned with comparative references and analysed phylogenetically using maximum likelihood approaches. Full details of read processing, mapping parameters, consensus curation, masking criteria, reference datasets and phylogenetic analyses are provided in the Figures S3–S6, S45 and S46.

#### 3.3.3 Modern Human DNA Contamination

Enriched and shotgun sequenced libraries were evaluated for their modern human DNA contamination. Those methods are detailed in the Tables S1–S3.

#### 3.3.4 Evaluating Hybridization Enrichment With a ‘Near‐Target’ Panel

To further evaluate PalaeoChip Arctic v1.0 performance with a ‘near target’ application, we conducted a series of analyses assessing bait‐to‐reference divergence, shotgun‐versus‐capture enrichment and sequence similarity between enriched sedaDNA reads and their corresponding baits. These analyses show that enrichment substantially increased recovery of target organelle sedaDNA relative to shotgun sequencing, but not as much as has been observed with regionally on‐target applications. Full details of the reference datasets, alignment parameters, enrichment calculations, divergence metrics and additional summary statistics are provided in the Figures S8–S13 and Table S4.

### 3.4 Core Chronology

The varve chronology for Crawford Lake was first established by Ekdahl et al. (2004) and others (Boyko‐Diakonow 1979; Ekdahl et al. 2004; Finlayson 1998a; Finlayson and Byrne 1975; McAndrews and Boyko‐Diakonow 1989; McAndrews and Turton 2007; McCarthy, Patterson, et al. 2025; Waters et al. 2024b), and core CL‐19 (which was investigated here) was first investigated by Llew‐Williams et al. (2024). We developed a revised Bayesian age‐depth model for the CL‐19 core using OxCal v4.4.4 (Bronk Ramsey 2009a) and the IntCal 20 calibration curve (Reimer et al. 2020). Our P_Sequence depositional model included both radiocarbon (*n* = 7) and ^210^Pb (*n* = 22) dates previously published in Llew‐Williams et al. (2024) and McCarthy et al. (2023), respectively. Our ^210^Pb ages were incorporated as calendar‐year estimates with associated analytical uncertainties and we assigned the topmost ^210^Pb age a modern calendar date which is consistent with the year of collection. We ran the model with a variable k parameter to allow independent determination of sedimentation rate variability (Bronk Ramsey 2008; Bronk Ramsey and Lee 2013). We applied a General_Outlier with a 5% (0.05) probability of any individual date being a statistical outlier (Bronk Ramsey 2009b). All of our modelled ages are presented as either median ages or as two‐sigma uncertainties (95.4% highest posterior density intervals) (Figure S14, Tables S5 and S6). We also re‐analysed the original radiocarbon dates from Ekdahl et al. (2004) using the same methods to provide an update with the IntCal20 calibration curve (Figure S15).

### 3.5 Stratigraphically Constrained Cluster Analysis (CONISS)

To delimit ecological assemblage zones, we applied stratigraphically constrained incremental sum‐of‐squares cluster analysis (CONISS) (Grimm 1987) to the classified sedaDNA read counts in R v4.5.1 (R Core Team 2025), using the *rioja* (Juggins 2024) and *vegan* (Oksanen et al. 2026) packages. Taxa were retained if they reached ‘supported’ confidence or higher in the *MetaMerge* aDNA‐support classification and were resolved to species, genus, family, subfamily or tribe rank; higher‐rank nodes, taxa occurring in fewer than three depths and negative‐control (blank) libraries were excluded. Retained read counts were summed by depth across replicate libraries to yield a depth by taxon matrix, which was Hellinger‐transformed before computing Euclidean distances, and zones were delimited by constrained agglomerative clustering (chclust, method = ‘coniss’). The number of statistically supported zones was evaluated against a broken‐stick model (Bennett 1996), and a four‐zone partition was adopted as the most ecologically interpretable solution and cross‐checked qualitatively against the broken‐stick. CONISS was run separately on (i) the on‐target plant and animal assemblage, (ii) the off‐target assemblage with bacteria/archaea, fungi and algae pooled (algae defined as off‐target ‘plant’‐lineage assignments excluding land plants, i.e., Streptophyta/Embryophyta) and (iii) a combined dataset in which the on‐ and off‐target matrices were each Hellinger‐transformed and then scaled by 0.5 before concatenation, so that the higher off‐target read counts did not dominate the shared distance metric (i.e., the two data types were weighted equally). Accompanying stratigraphic diagrams display the 20 most abundant taxa per analysis, each expressed as a percentage of the total retained assemblage at that depth (Figures S34–S38, Table S7).

## 4 Results

### 4.1 Palaeoecology and Authenticity

**FIGURE 2:**
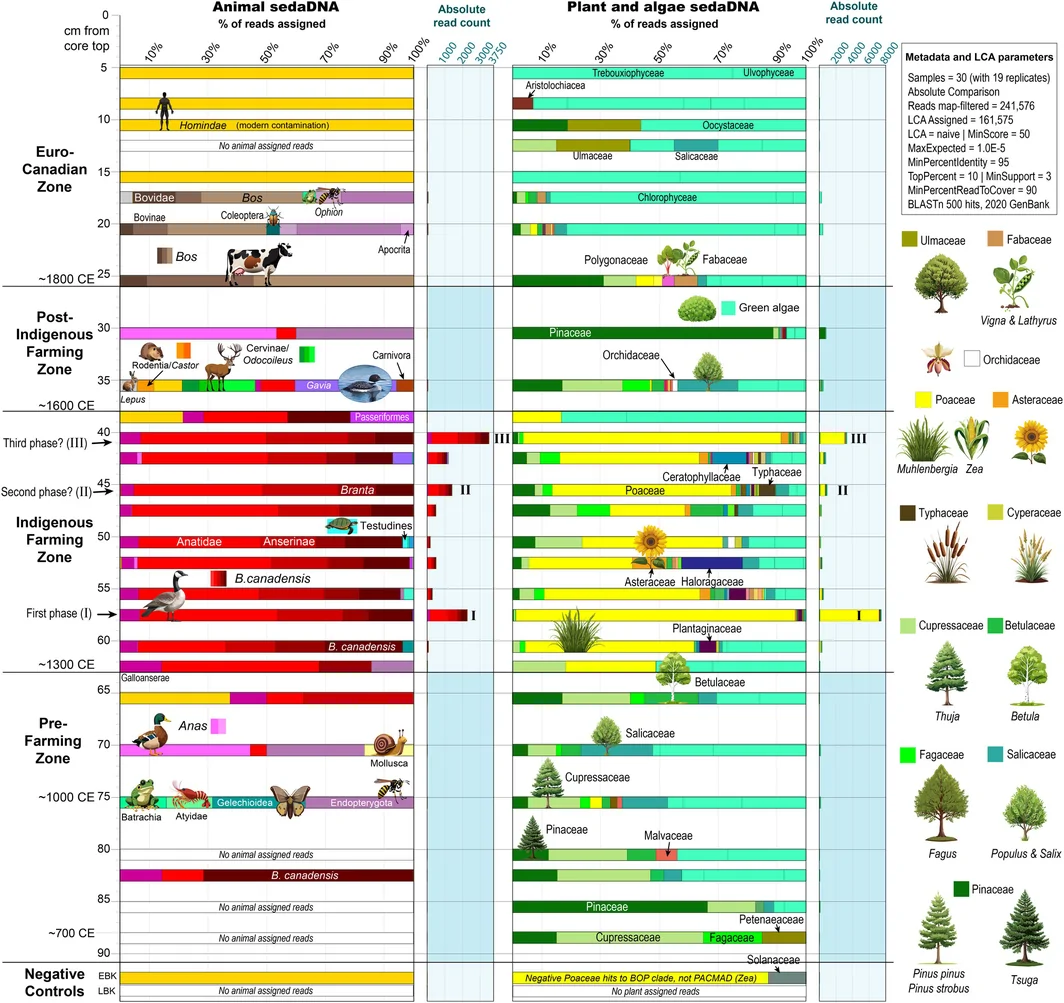
Phases of human ecology at Crawford Lake over the last ~1000 years. Assigned plant/algae and animal sedaDNA represented in stacked bar plots with proportional and absolute counts of classified reads. Cultural zones based on Ekdahl et al. (2004). Note: While > 24 bp was the pre‐filtering cut‐off, only reads > 30 bp are able to exceed the LCA parameters utilized here (Table S9, see the supplementary text for additional discussion and data and see Dataset S4 for *MetaMerge* authenticity support for on/off‐target taxonomic classifications). Phases of farming are inferred from their continuity with Ekdahl et al. (2004), although the precise dating of the second phase and post‐farming transition has an age incongruity necessitating further investigation. Ages here are estimations based on the CL‐19 age model (Figures S14 and S15).

**FIGURE 3:**
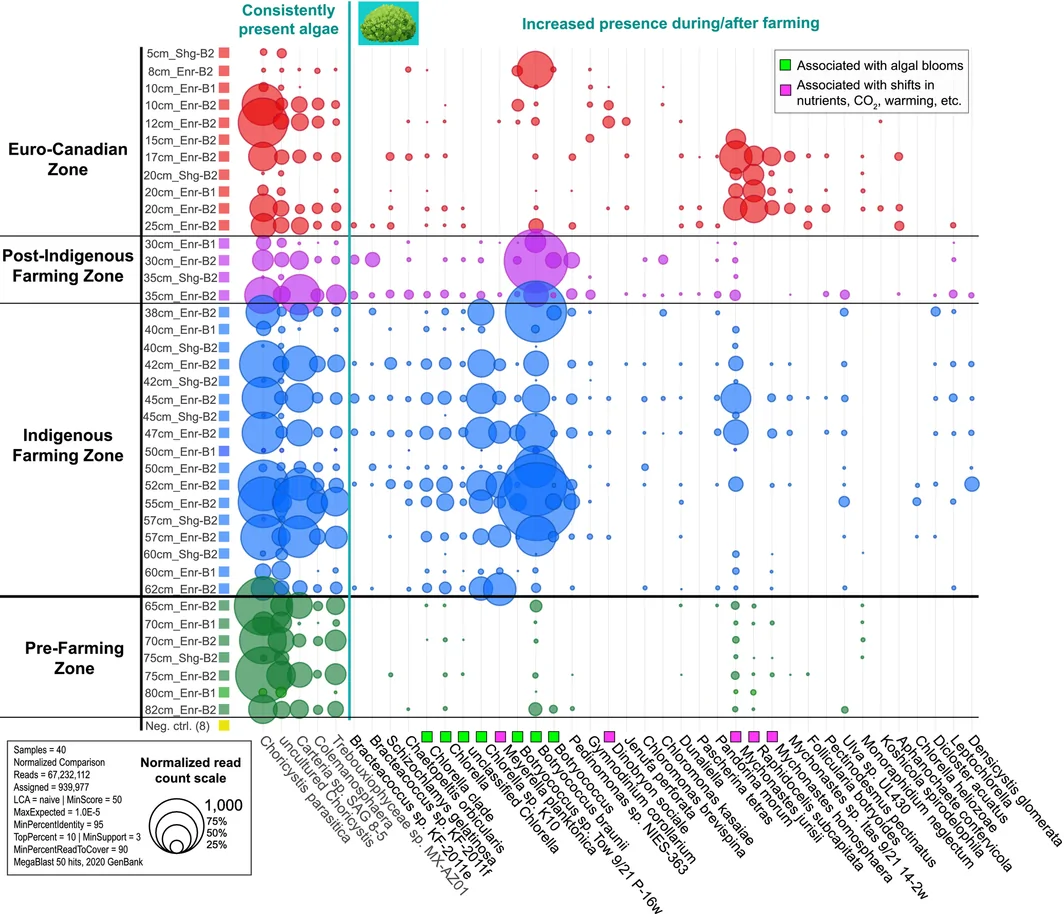
Bubble chart of select algae with clear indications of shifting abundance through the farming zones. Normalized read counts to the smallest library displayed with a square‐root scale. Note: Stratigraphic changes in sedaDNA read abundance can reflect variation in preservation, taphonomic processes, or library preparation efficiency alongside genuine biological changes, making it challenging to infer changes in biomass through sedaDNA. Consistently present algae are shown here to help support the interpretation that certain species known to be associated with algal blooms increase in abundance independent of the normalized background algal communities.

**FIGURE 4:**
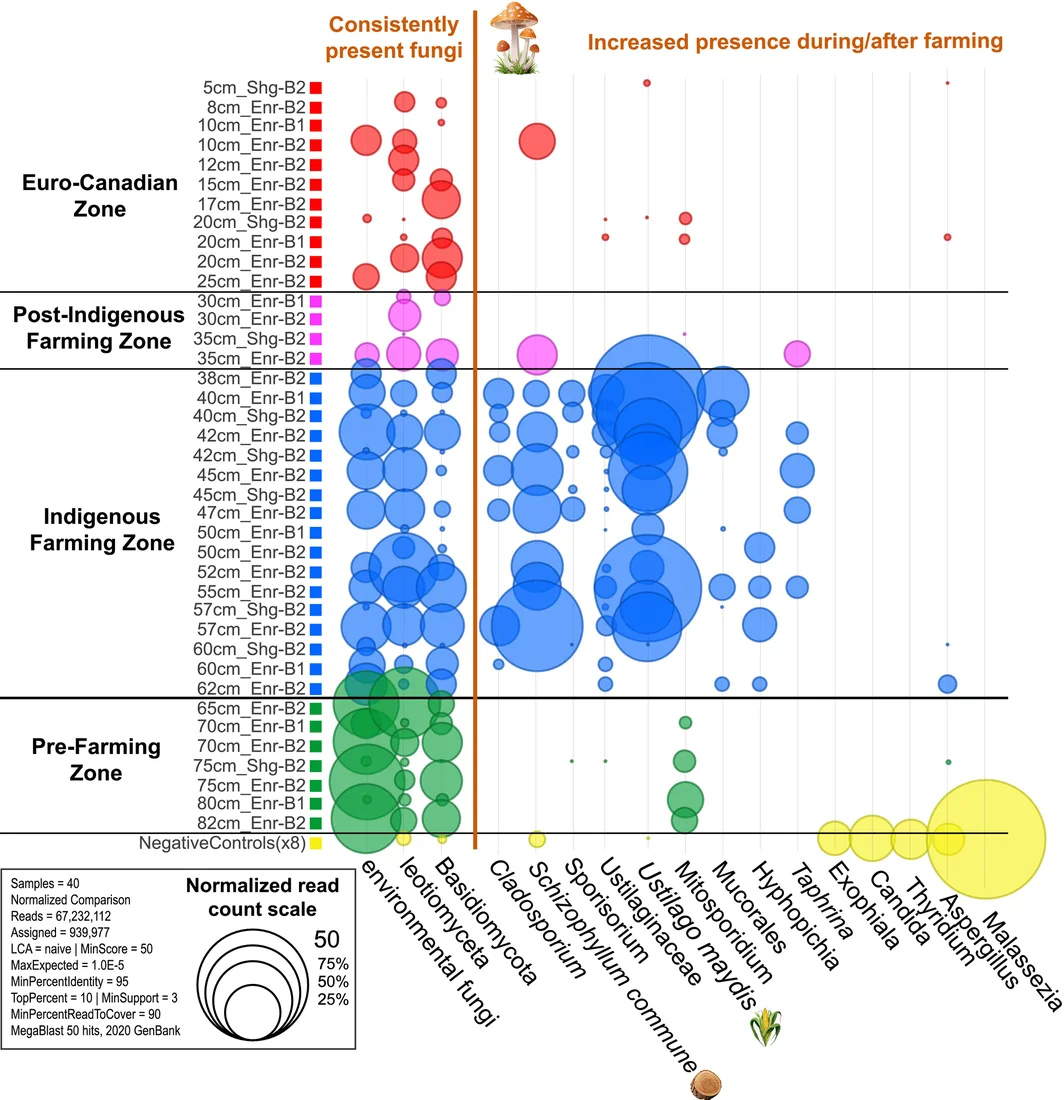
Bubble chart of select fungi with clear indications of shifting abundance through the farming zones. Normalized read counts to the smallest library displayed with a square‐root scale.

**FIGURE 5:**
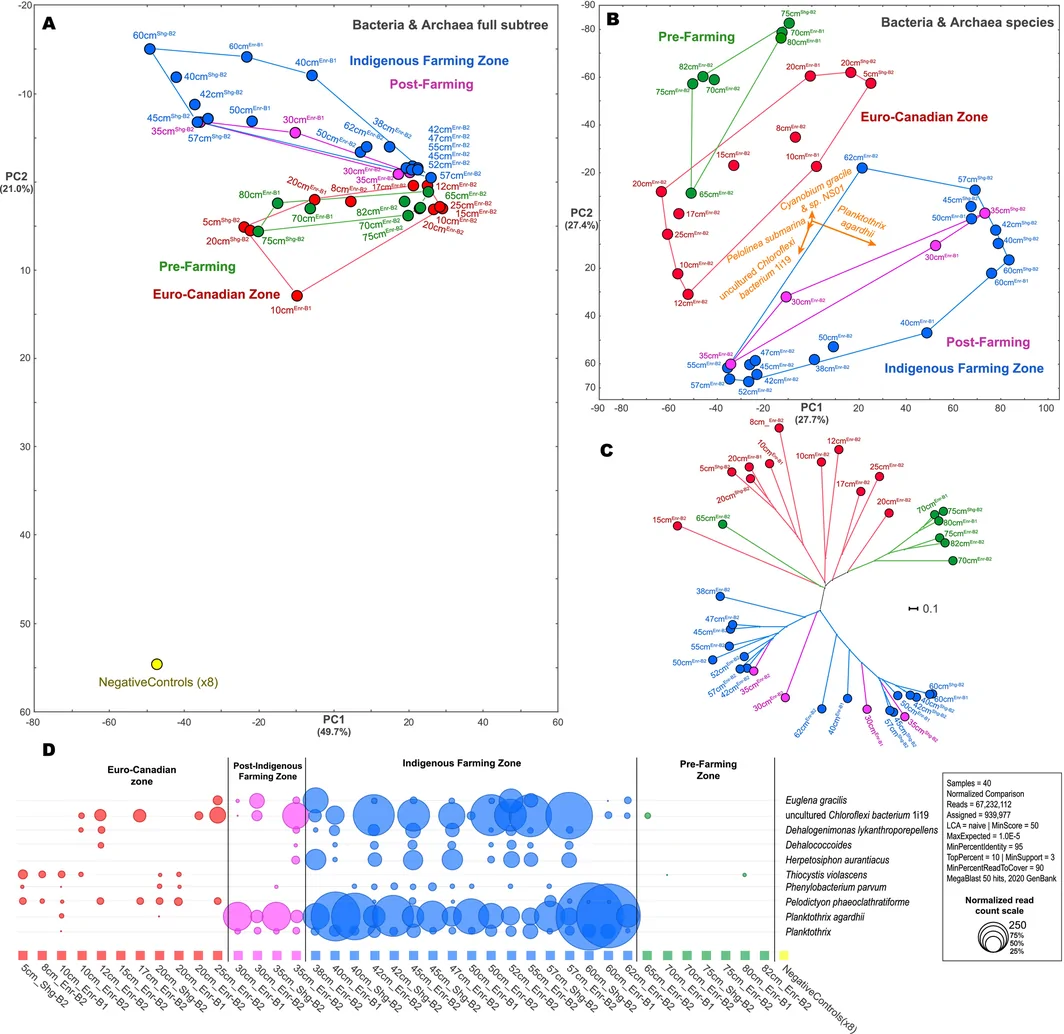
Cluster analyses of taxonomic hits to Prokaryotes (Bacteria and Archaea) using a χ^2^ ecological index. Counts normalized to the smallest library (156,805 post‐filtering reads, [150 K minimum]) using MEGAN7 compare. Depth‐based groupings associated with human ecological transitions are connected with colour‐coded convex hulls. Superscript indicates enriched (enr) or shotgun (shg) sequencing approaches and the extraction batch (batch 1 [B1] or batch 2 [B2]). Depth is centimetres from the top of the core (sediment–water interface). (A) PCoA of full Bacteria and Archaea subtrees (all ranks) including eight merged negative controls. (B) PCoA of species rank hits for prokaryotes. (C) Unrooted neighbour‐joining tree of metagenomic cluster analysis from B. (D) Bubble plot of select bacteria driving community shifts in B that show clear indications of shifting abundance through the farming zones. See Figure 3 caption regarding discussion of challenges inferring sedaDNA abundance shifts.

Our sedaDNA dataset clearly records ecological shifts associated with human activities at Crawford Lake over the last millennium with three to four clearly defined ecological zones: the pre‐farming zone, the Indigenous farming zone, a post‐Indigenous farming zone that has lingering impacts from farming but also clear evidence of ecological succession and a Euro‐Canadian zone with cattle farming and other impacts (Ekdahl et al. 2004; Figure 2). During the pre‐farming zone (base here of 88–89 cm, ~714–578 C.E.) we observe an overall low rate of sedaDNA preservation/input, with plant signals of Pinaceae (*Pinus* [pine] and *Tsuga* [hemlock], very high support), Fagaceae (*Fagus* [beech] and *Quercus* [oak], very high support), Salicaceae (*Populus* [poplar/aspen] and *Salix* [willow], supported), Cupressaceae (*Thuja* [cedar], very high support) and Betulaceae (*Betula* [birch], supported). Among animals we observe hits to Batrachia (frogs/salamanders, tentative to weak support), Atyidae (shrimp, weak support), Gelechioidae (moths, tentative support), Endopteryogta (metamorphosing insects, very high support), Mollusca (molluscs, weak support), *Anas* paltyrhynchos (mallard, supported) and *Branta canadensis* (Canada goose, very high support). Various green algae are identified including *Choricystis parasitica* (a mollusc algae parasite, very high support), *Carteria* sp. (supported), Trebouxiophyceae (including *Botryococcus braunii*, very high support) and *Mychonastes jurisii* (very high support, see Figure 3 and Dataset S4 for breakdown of 4000+ taxonomic identifications). Fungi identified include *Basidiomycota* (supported) and *Mitosporidium* (tentative support, see Figure 4), and bacteria/archaea communities form a distinct cluster, especially below 65 cm (Figure 5B).

During the onset of the Indigenous farming zone at ~62–65 cm (~1300 ce) we observe a significant increase in sedaDNA abundance with dominant signals of *B. canadensis* and Poaceae (very high support, with hits therein to *Zea mays* , supported) and an overall increase in plant diversity including Asteraceae (strong support, with hits to *Helianthus annuus* [sunflowers] supported), Plantaginaceae (plantains, supported to tentative), Haloragaceae (water milfoils, supported), Typhaceae (bulrushes, supported) and Ceratophyllaceae (supported, with *Portulaca* sp. [tentative support] known to be associated with the Indigenous farming zone). There appear to be two to three main pulses of sedaDNA: one around 57–58 cm, another around 45–46 cm and another around 40–41 cm. Ekdahl et al. (2004) had suggested that there were two phases of site occupation at the Crawford Village site occurred from 1325 to 1375 ce and 1410–1445 ce (Figures 1 and 2, Figure S1). Although the data in Ekdahl et al. (2004) also has evidence of a third pulse nearer to 1500 ce as discussed by Finlayson et al. (2026). If these pulses in sedaDNA that we observe here are associated with these occupation phases (Figure 2), our age‐depth model for core CL‐19 suggests these may have actually occurred somewhere between ~1320–1400 ce (Phase 1), ~1500 ce (Phase 2) and ~1600 ce (Phase 3) (Figure S14, Table S6). Our age‐depth model for core CL‐19 largely agrees with Ekdahl et al. (2004) except for the timing of the second, and potentially third, occupation phases and the transition to the post‐farming period. This period, including the village abandonment (~40 cm), is associated with large fluctuations in the radiocarbon calibration curve, which adds uncertainty to the precise aging of the later occupations and the post‐farming transition (Figures S14 and S15). Furthermore, Moraal (2024) found evidence for a massive flood ~1500 ce, which may contribute to the chronological discrepancy in this period. The age‐depth model for core CL‐19 has a much smaller error range than the original dates from Ekdahl et al. (2004) (Figures S14 and S15), suggesting that the farming to post‐farming transition may have occurred later than previously thought, although factors such as a lagging sedaDNA ecological response, shifted depositional patterns during the potential ~1500 ce flood, or a depositional hiatus in the CL‐19 core may be contributing factors. It is unclear if the second/third sedaDNA pulses do directly correlate with these proposed occupation phases or other unknown events. This discrepancy in the precise aging of the farming to post‐farming transition needs further investigation, although the overall trends observed here are sufficiently consistent for our millennia‐scaled investigation.

During the farming zone we see parallel increases in a range of green algae (e.g., *Chlorella* sp. (supported), *Meyerella planktonica* (weak support), *Botryococcus braunii* (very strong support) and *Mychonastes jurisii* (very strong support), and the dinoflagellate *Gymnodinium corollarium* (tentative support)), with several of these species known to be associated with algal blooms (Figure 5 and Dataset S4) (Aaronson et al. 1983; Fawley et al. 2004; Lu et al. 2023; Reñé et al. 2011; Smittenberg et al. 2005; Sundström et al. 2009; Sun et al. 2018; Teng et al. 2021; Zhang et al. 2022). Fungal reads also display an increase in the abundance of several taxa absent during the pre‐farming zone (Figure 3). Notably those include *Ustilago/Mycosarcoma maydis* (supported), a fungus that parasitizes maize causing the disease maize/corn smut (also an edible fungus called cuitlacochin in Mexico) and *Schizophyllum commune* (weak support), an opportunistic fungus that grows on rotting wood. Both are consistent with maize agriculture and land clearing for farming, although the *S. commune* signal lacks sufficient depth for authentication, has relatively low read identity, and is found in low abundance in the negative controls (see Dataset S4) and thus the *S. commune* signal is only a tentative identification that may be a form of background laboratory contamination. *Cladosporium* sp. (tentative support with low percentage identity) was also identified, a mould known to grow on sunflowers (Roberts et al. 1986; Sharfun‐Nahar and Hashmi 2005), as well as *Taphrina* (weak support), which is known to parasitize *Prunus* (plums/cherries) (Thangaraj et al. 2025). A small sedaDNA signal of *Prunus* sp. (tentative) was identified, which if accurate would have likely originated from the eastern woodland native species *Prunus nigra* (Canadian plum). Although these low support signals should be interpreted cautiously pending further investigation.

Bacteria and archaea follow the same trend with a distinct shift in overall community composition and a distinct increase in *Planktothrix agardhii* (very strong support), *Chloroflexi bacterium* (supported), *Herpetosiphon aurantiacus* (tentative support) and *Pelolinea submarina* (strong support; Figure 5 and Dataset S4).

We additionally recovered signals of *Ectopistes migratorius* (passenger pigeon, supported) at depth intervals of 42 and 45 cm (~1450–1600 ce) during the Indigenous farming zone. Passenger pigeons were once the most abundant bird in North America (estimated 3–5 billion individuals) and went extinct in 1914 (Hung et al. 2014). This detection represents a rare palaeoecological record from a lacustrine sediment archive during a period when passenger pigeons were historically common and often abundant in the Late Woodland archaeological record of southern Ontario (Orchard et al. 2023). This signal may be an important target for a new Eastern Woodlands bait‐panel for further investigating their sedaDNA record at Crawford Lake.

During the post‐Indigenous farming zone (above 40 cm) we see a rapid decline in sedaDNA abundance overall, along with the reappearance of wild animals including *Odocoileus* (white‐tailed deer, tentative support), Rodentia (with hits therein to *Castor* [beaver], tentative to weak support), *Lepus* (hares, weak support) and *Gavia* (loons, supported). Bacterial and algal communities that had increased during Indigenous farming persist intermittently in the post‐farming zone, whereas farming‐associated specific fungi disappear entirely. Loons nest in oligotrophic lakes because they need the water to be clear to forage, so it makes sense that the signal disappears during the onset of eutrophication and reappears in the post Indigenous Farming Zone when eutrophication of the lake diminished (Paruk et al. 2021).

During the Euro‐Canadian zone at 25–26 cm (~1800 ce) the predominant animal signal becomes *Bos taurus* (cattle, supported), which continues until 17–18 cm, along with beetles and *Ophion* sp. (Short‐tailed ichneumon wasps, tentative). All on‐target animal sedaDNA disappears above 17 cm, with only modern human DNA contamination detectable. We also observe the appearance of Polygonaceae (buckwheats, tentative to weak support) and Fabaceae (legumes, supported, with hits therein to *Vigna* [various beans and lentils, tentative] and *Lathyrus* [peavines, tentative]). Ulmaceae (elms, supported) appear at 12–13 and 10–11 cm, declining sharply above the proposed base of the ‘Anthropocene’ due to Dutch Elm Disease, although we do not observe sedaDNA from the causative fungal agents of Dutch Elm Disease (the ascomycete microfungi *Ophiostoma* spp.) (Karnosky 1979; Santini and Faccoli 2015). Most of the chloroplast signal at the lake during the Euro‐Canadian zone is chrysophycean algae with an increase in *Dinobryon sociale* (tentative) and *Ochromonas* sp. (tentative) at 10 and 12 cm at the base of the proposed ‘Anthropocene’ (Figure S2), along with *Mychonastes* spp. (very strong support), *Raphidocelis subcapitata* (very strong support) and *Cyanobium gracile* (tentative to supported) becoming abundant during Euro‐Canadian zone.

**FIGURE 6:**
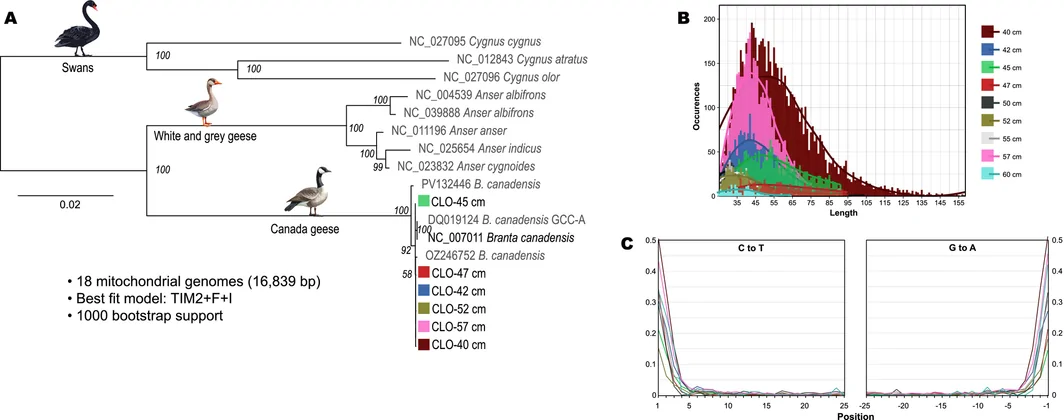
Mitogenomic assembly, phylogenetics and damage patterns of *B. canadensis* sedaDNA. (A) Maximum likelihood tree of publicly available goose mitochondrial genomes. (B) mapDamage fragment length distributions and (C) deamination rates of sedaDNA assemblies.

SedaDNA is sufficiently abundant during the Indigenous farming zone that we were able to reassemble six mitochondrial genomes of *Branta canadensis* (Figure 6). Only three mitogenomes exist for *B. canadensis* , so there is limited potential for phylogenetic investigation. Furthermore, there is little genetic diversity within the mitochondrial genomes of *B. canadensis* . The control region and cytochrome oxidase 1 (COX1) have very low levels of polymorphisms resulting in low genetic differentiation between individual geese samples analysed here, suggesting a very recent population expansion (Figure S3). Low haplotype diversity within a species can indicate that insufficient time has elapsed for new mutations to accumulate following a recent bottleneck or rapid expansion from a small founding population. When multiple individuals share identical or near‐identical haplotypes, the resulting shallow genetic differentiation is consistent with a recent common ancestry and limited accumulation of neutral variation, a pattern characteristic of recent demographic bottlenecks and expansions (Grant and Bowen 1998; Rogers and Harpending 1992; Slatkin and Hudson 1991). Mylecraine et al. (2008), found that mtDNA genes and microsatellites were insufficient to distinguish between populations of *Branta*, which corresponds to what we observe here using both modern and ancient samples.

The constrained cluster analyses of ensemble supported classified taxa resolves three broad ecological assemble zones (Table S7). The Pre‐Farming zone (below ~62–65 cm), represents the baseline forested catchment prior to Indigenous agriculture. Above it, an Indigenous Farming zone (~25–62 cm) is marked by turnover toward disturbance‐ and agriculture‐associated taxa, including maize (*Zea*) and *Branta canadensis* , consistent with the documented interval of maize horticulture by Longhouse People. The uppermost Euro‐Canadian/‘Anthropocene’ zone (above ~15–25 cm) records a further assemblage shift coincident with European settlement, catchment clearance and modern nutrient enrichment, including livestock‐associated taxa (e.g., *Bos*) and industrialization. Notably, our multi‐kingdom analyses resolved complementary boundaries: the on‐target (plant and animal) assemblage most strongly separated the Pre‐Farming and Indigenous Farming transition at ~62–65 cm, whereas the off‐target (microbial, fungal and algal) assemblage most strongly separated the Indigenous farming and post‐framing from the Euro‐Canadian transition at ~20–25 cm. Neither boundary is abrupt, which is as expected where plant/animal and microbial/algal turnover are not perfectly synchronous, but together the two independently‐supported breaks consistently delimit the same three‐zone structure, with an interpreted post‐farming zone interval being useful to help describe ecological succession prior to Euro‐Canadian impacts at the lake. The ‘Anthropocene’; boundary above 15 cm did not receive broken‐stick model support, however it did consistently separate in the CONISS analyses. Our wet‐lab and bioinformatic approaches here are tailored to highly degraded ancient DNA and are not optimal for recent (< 100 year) environmental DNA. We have found in other ongoing research efforts (not yet published) that the upper portion of our lake sediment cores tend to perform very poorly with our ancient DNA optimized methods (data not shown). Thus, a technique optimized for modern/historic environmental DNA is needed to properly explore the ‘Anthropocene’ boundary, which was not the objective of this investigation.

**FIGURE 7:**
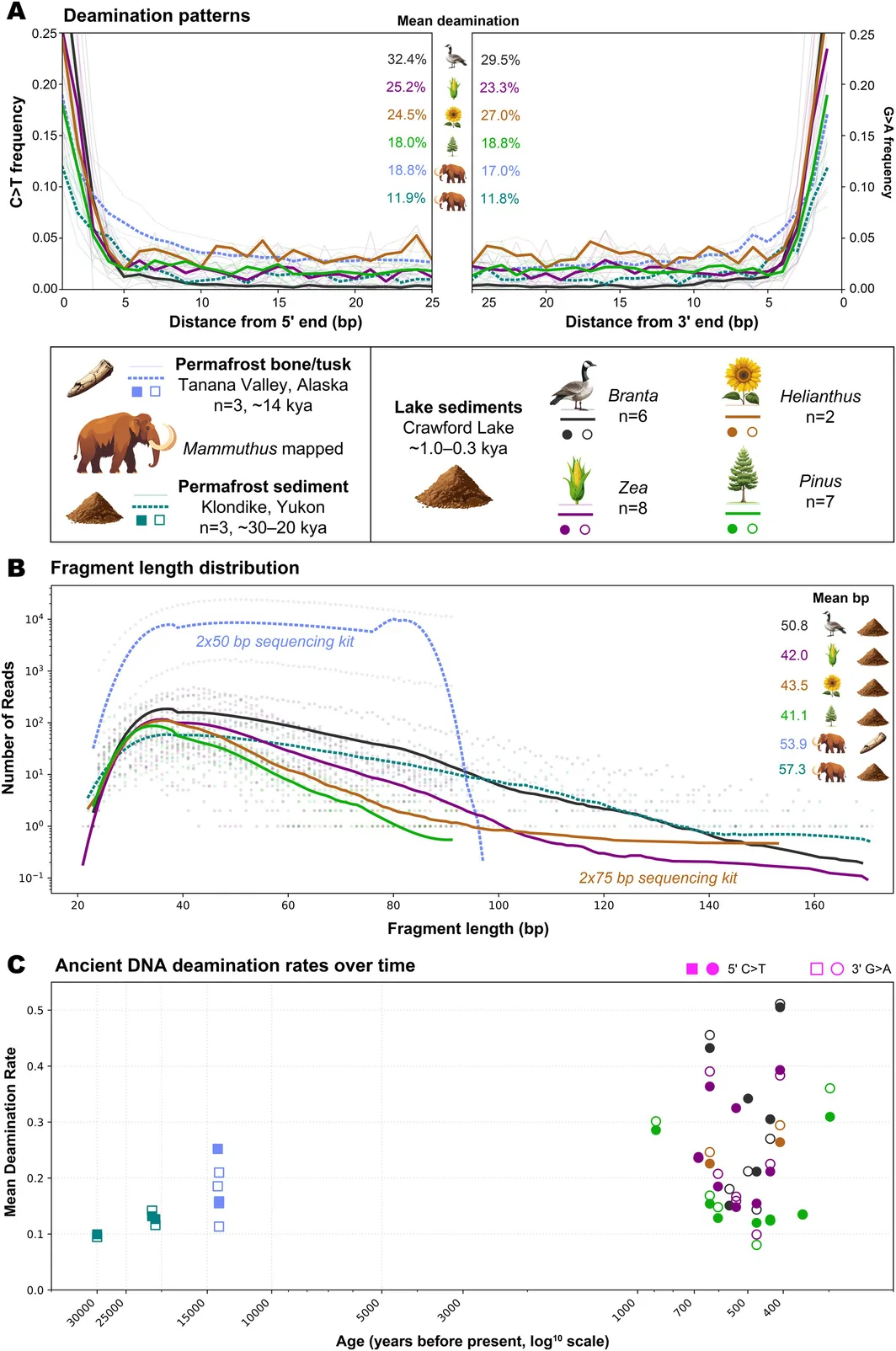
Rates of DNA damage comparing lake sediments and permafrost preserved sediments and bone. (A) misincorporation plot, (B) fragment length distributions and (C) deamination rates over time showing that age alone is a poor predictor of aDNA damage without also considering the burial environment.

Both extraction and library controls consistently separate out from our lake sediment samples in all metagenomic analyses, indicating that we are measuring the ecological communities of the sediments themselves, not the background signal of our plasticware, reagents and laboratory settings. The only signals in our on‐target negative controls are sporadic human, BOP clade grasses (not the maize clade), and Solanaceae (nightshade) (Figure 2). Furthermore, we observe surprisingly high rates of deamination among *Branta* mapped reads (~15%–50%), with mitogenome assemblies from the two peaks of Indigenous agricultural settlement (40 cm and 57 cm) having > 40% of reads deaminated (Figure 6). High rates of deamination are also observed with the barcoding gene assemblies (*rbc*L, *trn*L and *mat*K) for *Z. mays* (Figure S4), *Helianthus annuus* (Figure S5) and *Pinus* sp. (likely eastern white pine) (Figure S6). These targeted chloroplast genes are also highly conserved, and so few polymorphisms again exist for detailed phylogenetic investigations without an ancient comparative dataset. Several of the Crawford *Zea* assemblies sit basal to modern corn reference sequences (Figure S4), suggesting that with an improved dataset including ancient corn and larger genomic assemblies, investigating ancient agricultural trade may be possible. Our most abundant taxa have sufficient depth of coverage and signals of damage to exceed *metaDMG* thresholds for being ancient (Figure S7). The high rates of deamination and exceptionally short fragment lengths, paired with distinct metagenomic signals between samples and negative controls, support that the signals we observe in our dataset originate from the sediments themselves are not in any substantive way impacted by contamination. The rates of damage observed for the *Branta* mapped sedaDNA from Crawford (~0.5 thousand years old [kya]) exceed that of bones and sediment that are ~15 kya and ~20–30 kya, which clearly demonstrates that burial conditions are far more important in terms of DNA damage than age alone (Kistler et al. 2017; Figure 7).

### 4.2 Bait Performance

We evaluated the performance of the PalaeoChip Arctic v1.0 hybridization bait‐panel for this eastern woodland sedaDNA dataset by investigating three sets of analyses. (1) Comparing bait‐to‐reference sequence divergence (calculated as edit distances) among Arctic targeted organisms that the bait‐set was designed for compared with off‐targeted eastern woodland taxa we were hoping to enrich for here. (2) Evaluating the proportional fold enrichment of mapped and LCA assigned reads in our bait‐capture data relative to the shotgun data. And (3) comparing how well the captured sedaDNA reads matched the baits and original reference targets (sedaDNA‐to‐bait and sedaDNA‐to‐RefSeq). A full cataloguing of these analyses can be found in the Supporting Information.

**FIGURE 8:**
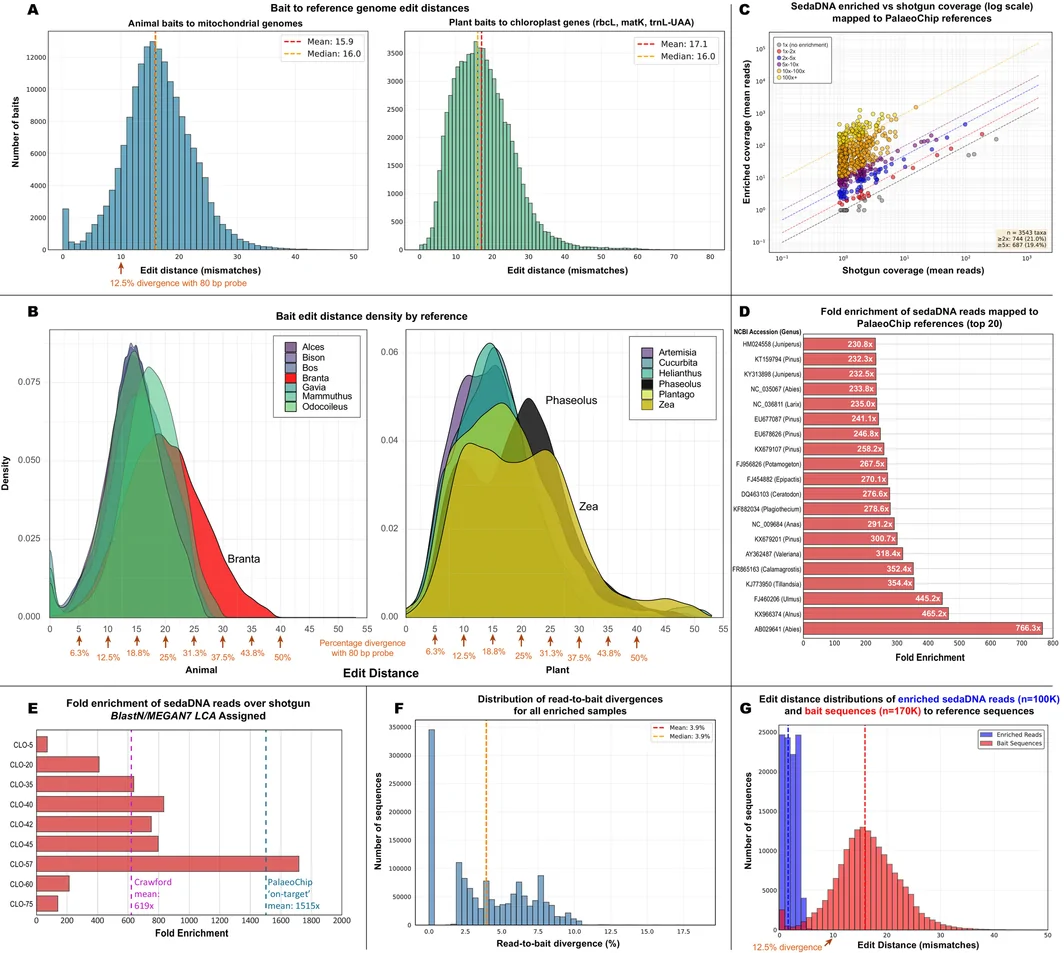
Bait capture using a regionally near‐target panel. (A) Edit distances (mismatches) between animal and plant PalaeoChip Arctic v1.0 baits and a representative subset of original reference sequences. (B) Edit distance in A by select taxon and density with partial emphasis on woodland taxa absent from original bait design. (C) Enriched vs. shotgun coverage with sedaDNA reads mapped to original bait reference sequences. (D) Fold enrichment of Crawford Lake sedaDNA reads mapped to PalaeoChip reference sequences and (E) fold enrichment of *BlastN*‐MEGAN7 LCA‐assigned reads. (F) Divergences of sedaDNA reads to bait sequences and (G) edit distances between enriched sedaDNA and PalaeoChip reference sequences plotted alongside edit distances between the baits and their original PalaeoChip reference sequences.

1. We observed bait‐to‐reference edit distances for animals that ranged from 14.5 mismatches (18.2% divergence) for *Alces* to 20.1 mismatches (25.1% divergence) for *Branta* (Figure 8A,B, Figure S8). The mean distance across 7 taxa was 15.9 ± 6.1 mismatches, corresponding to 19.9% ± 7.7% divergence. We mapped between 15,092 baits (*Gavia*) and 28,717 baits (*Bos*) per taxon, with most taxa represented by > 25,000 baits. For plants, BED‐restricted analysis targeting only *rbc*L, *mat*K and *trn*L‐UAA genes yielded per‐taxon mean divergence ranges from 15.0 mismatches (18.8% divergence) for *Artemisia* to 19.5 mismatches (24.3% divergence) for *Zea* (Figure 8B). The mean across all six plant taxa was 17.1 ± 8.3 mismatches, corresponding to 21.3% ± 10.3% divergence. Plant taxa showed comparable variability to animals in both mean divergence and standard deviation (see supplementary datafile: Dataset S1). In this case, woodland taxa were within the same overall range of divergence, but there were more mismatches between the baits and references for woodland organisms compared with their arctic counterparts for which this panel was designed.
2. We observed a fold enrichment of ~200×–700× among the top 20 of 3543 mapped taxa in the reference sequences the baits were designed from (Figure 8C–E, Figures S9 and S10, see supplementary datafile: Dataset S2). A total of 2751 (77.6%) of mapped taxa in the enriched dataset had no shotgun mapped reads. When analysing the *BlastN*‐to‐*MEGAN7* LCA assigned reads, there was an average fold enrichment of 619× over equivalent shotgun libraries (Figure 8E). Murchie, Kuch, et al. (2021) and Murchie, Monteath, et al. (2021) observed an average 1515× fold enrichment comparing replicate shotgun and capture enriched libraries. In that case, those data were from permafrost preserved sediments containing the taxa from which this bait‐panel was initially designed. Therein, while enrichment was clearly successful here, it underperformed when used for near‐target taxa in this application. While it is not possible to account for the many factors impacting eDNA input and preservation between this study and Murchie, Kuch, et al. (2021) and Murchie, Monteath, et al. (2021), we expect an improved fold enrichment with the development of a more spatial‐temporally specific bait panel.
3. We observed 4,218,307 reads mapping to the reference sequences with a mean edit distance of 1.1 ± 1.2 mismatches and mean divergence of 2.7% ± 3.0% (Figure 8G, Figures S8–S11). The proportion of reads with divergence ≤ 10% was 99.6% (4,200,230/4,218,307 reads). The mean read length for the FullRefs analysis was 38.1 ± 7.0 bp (see Dataset S3). This highlights that while baits may have on average 15 mismatches (~19% divergence) relative to the original reference sequences, those baits will still successfully hybridize and enrich for reads that are near perfect matches to the original references (allowing for genomic assemblies). Given the 2.4× reduced fold enrichment observed here (619×) relative to Murchie, Kuch, et al. (2021) and Murchie, Monteath, et al. (2021) (1515×) (Figure 8E), we can observe that exceeding a divergence of ~25% (Figure 8B) does result in reduced hybridization enrichment and the need for a more locally specific bait panel, although factors such as especially high levels of deamination also likely impacted the hybridization efficiency here.

## 5 Discussion

### 5.1 Palaeoecology and Human Settlement

Crawford Lake closely records the anthropogenic impacts on a whole ecosystem over the past ~1000 years through the preservation of sedaDNA from terrestrial/aquatic plants, animals, algae, fungi and bacteria. Our data correspond closely with previous investigations of the lake's sediments and excavations of the nearby village sites (Boyko‐Diakonow 1979; Boyko 1973; Ekdahl et al. 2004, 2007; Finlayson and Byrne 1975; Gushulak et al. 2021; McAndrews and Boyko‐Diakonow 1989; McAndrews and Turton 2007, 2010; McCarthy et al. 2023; McCarthy, Patterson, et al. 2025; Turton and McAndrews 2006). Late Woodland Longhouse People were farming maize and sunflowers (among many other cultigens) near Crawford Lake between the 13th and 16th centuries CE. As was previously identified by McAndrews and Turton (2007), wild Canada goose foraged in those cultivated fields, roosting on Crawford Lake and depositing large numbers of dung pellets with cultigen DNA and pollen into the lake basin over the entire farming zone period (McAndrews and Turton 2007). This goose‐driven nutrient influx likely led to the repeated eutrophication of the lake, which we observe here in the algal and bacterial community shifts during periods of farming (Ekdahl et al. 2004, 2007). These shifts in lake ecology persisted even after abandonment in the 16th century with DNA signals of pine trees, rabbits, deer, beavers and loons, eventually returning while agricultural grasses (maize) remained absent. A second eutrophication is observed in strata recording Euro‐Canadian farming and logging (19th century) including cattle and legumes, and a similar (but shifted) community of algae, bacteria and fungi. The dominance of green algae and chrysophytes (golden algae) in the upper part of the Euro‐Canadian Zone is characteristic of the high CO_2_ environment that characterizes the Great Acceleration (McCarthy et al. 2026).

Notably, bean and squash DNA were absent from our sedaDNA dataset during the Indigenous farming zone aside from a small (< 5 read) signal of *Phaseolus* (weak support, 57 cm) in the off‐target classifications. Macrofossil and palynological work at the village found the full array of Three/Four Sisters Agriculture (maize, beans, squash and sunflowers), with the Crawford Lake site being among the first with clear evidence of bean cultivation in the region (Finlayson and Byrne 1975). This lack of squash and bean sedaDNA may be a result of less DNA input from those species into the lake (such as those species being a less desirable food for Canada goose), beans and squash having less eDNA shedding, or a lack of chloroplast enrichment of those taxa due to their absence from our bait‐set. The closest baits that could have caught the DNA of beans (*Phaseolus*) have a divergence mode of ~30% (Figures 8, S8–S10), which would result in poorer bait‐to‐target hybridization and contribute to a lack of reads matching these references. *Zea*, *Branta* and *Phaseolus* references all have greater divergence with the bait panel compared to organisms that were the primary target of the initial panel design such as *Mammuthus*, *Artemisia*, *Plantago* and *Bison* (Figure S8). This highlights the utility and necessity of developing an Eastern Woodland panel for future research that would have higher binding affinity for regionally and culturally important organisms. A regionally specific bait‐set designed with Indigenous informed plant and animal species based on oral narratives, palynology and archaeological remains from this site and others nearby would be of benefit for further study of this lake, with the potential for recovering a greater diversity of species beyond those identified here. McAndrews and Turton (2007) found that more crop pollen was transferred to the lake from geese than wild dispersal, indicating that Canada goose is likely a primary mechanism in the abundance of lake sedaDNA observed here and the resultant eutrophication. While a regionally specific bait panel would likely improve the capture of informative sedaDNA from the village, the release, dispersal and preservation of environmental DNA has been found to be sufficiently heterogeneous that some key agricultural taxa may have large sedaDNA signals in the lake, while others cultivated with similar intensities may have weak or absent signals as a result of the complex and insufficiently understood taphonomy of environmental biomolecules (Dabney, Meyer, and Pääbo 2013; Garcés‐Pastor et al. 2023; Giguet‐Covex et al. 2023; Lorenz and Wackernagel 1987; Massilani et al. 2022; Mitchell et al. 2005; Murchie, Giguet‐Covex, et al. 2023; Parducci et al. 2017). The very high levels of deamination observed here suggest significant DNA degradation, which would also impact sedaDNA recovery regardless of bait specificity.

Oral‐ and ethno‐historic accounts of the eastern woodlands describe the use of fire to open land for agriculture (Onion 1964). Consistent with this, previous palaeoecological research found evidence of forest clearance and land management using fire around Crawford Lake during the Indigenous farming zone (Clark and Royall 1995), and in lake sediment cores, an increase in charcoal particles coincides with the rise of cultigen pollen during the 14th–15th century settlement, signalling that the inhabitants used controlled burning to clear fields and improve soil fertility (Clark and Royall 1995; McAndrews and Turton 2007). Lake sediment DNA shows a coincidental decrease in tree sedaDNA during this zone (Figure 4). Reads mapping to *Schizophyllum commune* (an opportunistic fungus that grows on rotting wood) are present, but only have tentative authenticity support. *S. commune* was also detected in the negative controls (normalized count = 1.05), indicating some level of laboratory or airborne background contamination. The blank signal is low relative to the sediment (maximum sediment count is 30.7 normalized reads in CLO‐57 versus 1.05 in the negative control), and *S. commune* is a cosmopolitan wood‐decay basidiomycete whose concentration in the Indigenous farming zone is ecologically plausible (due to forest clearance). However, the *S. commune* signal lacks sufficient depth for authentication and thus we cannot interpret these reads as direct evidence of land clearance. The decrease in tree sedaDNA paired with the overall ecological signal is consistent however with other palaeoecological lines of evidence for forest clearance and land management.

Archaeological surveys have identified a sequence of village sites in the region, reflecting movement of communities roughly every ~15–40 years (Finlayson 1998a). Finlayson and Byrne (1975) hypothesized village relocations from an earlier site (5 km to the southwest) to the larger Crawford Lake village, and possibly several additional sites that continued farming in the vicinity until sometime in the 16th century CE. The Crawford Lake sediment record supports these settlement and abandonment cycles, with two to three distinct pulses of site occupation suggested by increased goose and grass sedaDNA. Dating discrepancies between core CL‐19 and Ekdahl et al. (2004) indicate that further work is needed to resolve the timings of the settlement phases and site abandonment, although accurate radiocarbon dating is hindered by high sunspot activity in the latter part of the Indigenous farming interval (Finlayson et al. 2026; Llew‐Williams et al. 2024). Overall these patterns align with Late Woodland agricultural practices where fields were typically left to fallow and villages moved as local resources and soil fertility declined, allowing secondary forest succession in former clearings (Finlayson 1998a). By ~1600 ce (~39 cm), maize sedaDNA and pollen disappear from the Crawford Lake varves, implying that Indigenous agriculture had ceased (or at least significantly slowed) in the immediate watershed (McAndrews and Turton 2007). This corresponds with broader regional trends as local Indigenous populations moved to new areas and the Crawford Lake ecosystem went through secondary succession prior to European arrival.

After three centuries of forest regeneration, Crawford Lake's watershed again experienced significant human impacts with Euro‐Canadian colonization in the 19th century. Previous palynological data and our sedaDNA records indicate that significant land‐use changes began around the mid‐1800s with a rise in *Ambrosia* (ragweed) pollen, a decline in tree pollen and erosional indicators reflecting forest clearance for agriculture (Ekdahl et al. 2007; McAndrews and Boyko‐Diakonow 1989). By the 1860s much of southern Ontario, including the Niagara Escarpment area, had been logged or cleared for timber and farmland. The palaeoecological imprint of 19th century logging is evident in Crawford Lake's varves: in addition to the pollen changes, there is a modest rise in charcoal concentration during the Euro‐Canadian period, suggesting that slash‐burning or accidental fires accompanied deforestation, along with lumber milling (Clark and Royall 1995). We observe the appearance of cattle sedaDNA during the 19th century, along with tentative hits to Polygonaceae (buckwheat family) and supported tentative hits within Fabaceae (legume family). Previous analyses of fungal spores from the site found no indication of a rise in grazing herbivores (McAndrews and Turton 2010), most notably the dung fungus *Sporormiella* (Davis and Shafer 2006; Raper and Bush 2009). The absence of these spores contradicts the presence of distinct cattle sedaDNA during this zone, suggesting that cattle grazing intensity was either further afield from the lake or their density may have been relatively low overall near the lake. This highlights the utility of sedaDNA paired with palynology for complementary ecological insights.

We observe an increase in multiple algal and cyanobacterial taxa during both the Indigenous farming and Euro‐Canadian zones relative to the pre‐farming interval, consistent with previous Crawford Lake proxy records and with the expected ecological response to increased nutrient delivery into the lake basin following land‐use change (Ekdahl et al. 2004, 2007). Importantly, we do not interpret the presence of individual bloom‐associated taxa alone as evidence of eutrophication, as many of these organisms can also occur in non‐eutrophic freshwater systems. Rather, we interpret eutrophication here cautiously as a stratigraphic and community‐level signal, supported by the coordinated increase of multiple algal, cyanobacterial and freshwater bacterioplankton taxa, their increased relative abundance after the onset of farming and their temporal correspondence with independently documented phases of forest clearance, agriculture, sedentism and catchment disturbance. This is in addition to the previously identified association of abundant Canada goose dung in the lake and its contextual association with periods of cultural eutrophication (Ekdahl et al. 2004, 2007; McAndrews and Turton 2007).

Within the Indigenous farming zone, *Botryococcus braunii* , *Chlorella* sp. and *Meyerella planktonica* show notable increases in abundance, while *Mychonastes* spp. and *Raphidocelis subcapitata* become more prominent during the Euro‐Canadian interval. *Chlorella* sp. and *Botryococcus braunii* include taxa commonly associated with bloom‐prone or nutrient‐enriched freshwater conditions (Aaronson et al. 1983; Lu et al. 2023; Smittenberg et al. 2005; Teng et al. 2021; Zhang et al. 2022) with sedaDNA abundance increasing after farming begins, peaking between 57 and 50 cm and at 38 cm and increasing again at 30 cm during the post‐farming phase. While *Botryococcus braunii* remains present in the Euro‐Canadian zone, the algal signal during the most recent farming interval appears more strongly associated with *Mychonastes jurisii* and *Raphidocelis subcapitata*. *R. subcapitata* is widely used as a freshwater ecotoxicology model organism for assessing pollutant effects (Machado and Soares 2024), although its occurrence here is interpreted cautiously as part of the broader community shift rather than as an independent pollution indicator.

At the base of the proposed ‘Anthropocene’ (~10–12 cm) we observe an increase in *Dinobryon sociale* and *Ochromonas* sp., two ‘golden algae’ (chrysophyte) taxa that have been observed to increase with the rise of mid‐20th century atmospheric CO_2_ (Figure S2; McCarthy et al. 2026). *Planktothrix agardhii* , a cyanobacterium frequently associated with harmful algal blooms in freshwater ecosystems, is also a major contributor to microbial community clustering among the Indigenious farming zone (Figure 5) (Churro et al. 2017; Lenard and Poniewozik 2022; Yéprémian et al. 2007; Zhang et al. 2020). Additional freshwater bacterioplankton, including *Polynucleobacter* (characteristic of eutrophic and bloom affected lake environments), recovered across 24 libraries with supported damage signatures (7.6%, significance = 3.1), and *Pelolinea submarina* (Chloroflexi, high confidence), recovered across 31 libraries with 18.7% C‐to‐T damage (significance = 2.1), further support a deaminated sedimentary microbial signal as Chloroflexi‐affiliated lineages are associated with anoxic or oxygen‐depleted conditions that develop during cyanobacterial bloom events (Tang et al. 2022; Xiao et al. 2017). The upper sediments also contain *Cyanobium gracile*, a picocyanobacterium that can also be associated with algal blooms (Genuário et al. 2016; Kim et al. 2025). Together, these results suggest that farming‐associated nutrient loading and catchment disturbance contributed to repeated shifts toward enriched freshwater conditions, while recognizing that eutrophication in this case is inferred from the combined ecological, stratigraphic and archaeological context rather than from the presence of any single organism (Ekdahl et al. 2004, 2007).

### 5.2 Ancient DNA Authenticity

There are substantial rates of deamination (nucleotide base misincorporations) in this metagenomic dataset, which are interesting given the relatively recent age of the sediments (Figure 7). By comparison, in 20–30 kya permafrost‐preserved loessal silts from the Yukon, Murchie, Kuch, et al. (2021) and Murchie, Monteath, et al. (2021) observed deamination rates of ~10%–30%, and bones or even coprolites aged to 700,000 years ago have deamination rates of ~40%–45% (Murchie et al. 2026; Orlando et al. 2013). Here by contrast, several of the *B. canadensis* assemblies have deamination rates over 30% and as high as 52% in as little as 500 years. High deamination rates are also observed in the corn, sunflower and pine tree assemblies (Figures S4–S6). This underscores the non‐linear rate of DNA damage accumulation and that burial conditions, most of which we still do not understand, are more important than age when assessing ancient DNA degradation for purposes of authenticity (Allan et al. 2021; Dabney, Meyer, and Pääbo 2013; Jia et al. 2022; Kistler et al. 2017; Schwarz et al. 2009; Smith et al. 2003). In this case with lake sediments, deamination and depurination are most likely hydrolytically induced, and as such lake sedaDNA is expected to be more damaged than, for example, permafrost terrestrial deposits. The pH of the Crawford Lake water column was observed to range between 5.8 and 8.5 in 2020–2021 (Llew‐Williams et al. 2024). The more acidic end of that range likely contributes to increased damage, but the pH is not low enough on its own to explain the very high rates of deamination observed here, which is likely contributed to by other factors such as water content, temperature, mineral absorption and microbial activity, along with the gut to dung pellet Canada goose depositional pathway. It is likely that the increased sedaDNA abundance we note during the farming phase is due to higher DNA inputs into the lake because of land clearance, erosion and high rates of dung deposition from large goose populations feeding on fields of maize and sunflowers. It is unclear what other geochemical or biological factors may have shifted rates of sedaDNA degradation.

### 5.3 Bait Performance and Phylogenetics

Our initial hypothesis was correct that a regionally adjacent bait‐set would likely be similar enough to successfully enrich for palaeoecologically informative sedaDNA. We observe an average 619× fold enrichment of LCA assigned reads compared with replicate shotgun libraries, even for agricultural plants that were intentionally excluded from the initial bait‐design. RNA baits will hybridize to near‐target sedaDNA, with > 1000‐fold enrichment, even among 18%–25% average bait‐to‐reference divergence for organisms such as woolly mammoths. This helps validate the use of broad bait‐panels such as PalaeoChip to recover the sedaDNA of organisms not represented in the original bait design. As expected, however, there is a limit to this off‐target capture as exceeding ~25% mean bait divergence does result in reduced fold enrichment (619× vs. 1515×) (Murchie, Kuch, et al. 2021). This is also likely compounded by the high levels of DNA degradation observed here as exceptionally high deamination rates (up to ~50% C‐to‐T deamination) and short fragment lengths could independently reduce hybridization capture efficiency since deaminated fragments form additional mismatches with intact bait sequences. Hybridization temperature could also be experimented with to improve recovery in near‐target scenarios, but this can have the negative effect of increasing far off‐target hybridization and reducing the fold enrichment. We found that our libraries enriched with a single round of capture performed well, but that two rounds of capture resulted in significant PCR duplication which reduced yield (Figure S45). Although this was not intended as a methods comparison and as such, confounding variables were not controlled for to test methodological outcomes between variable hybridization rounds. Further research is needed to fully optimize capture enrichment conditions for broad ecological sedaDNA applications, including factors such as optimal bait identity composition, hybridization temperature and duration and the number of hybridization rounds when using large multi‐organism panels. Overall though, our investigation suggests a more regionally specific panel for the eastern woodlands would potentially be able to recover twice as much palaeoecologically informative sedaDNA from plants and animals compared with the arctic panel used here, let alone the fold enrichment expected for entirely non‐target organisms such as fungi, insects and algae. There is likely significantly more ecologically interesting sedaDNA preserved in the sediments deposited in Crawford Lake than we report here, which would be accessible with either very deep shotgun sequencing or the development of a Late Woodland Period bait‐panel.

Despite the near‐target panel, we were able to recover genomic scale data for multiple organisms. However, few polymorphisms exist within the organisms' genomic regions we have targeted here to allow for meaningful phylogenetic investigations of population history. A refined plant bait‐set for the region would be best designed with a larger genomic scope (ideally full chloroplast genomes or nuclear DNA) as the *trn*L gene here tended to have high levels of bait divergence (> 30%) when targeting a highly diverse set of species, resulting in low coverage overall (Figures S8–S10), whereas *rbc*L and *mat*K generally lack the genetic variability needed for species‐level identification or population genetic investigation. Given the abundance of maize DNA, if an aDNA comparative collection of maize chloroplast genomes were assembled from archaeological sites further south into Mesoamerica, one could potentially phylogenetically place the maize grown at Crawford Lake to help inform our understanding of maize horticultural trade networks in the Americas during the Late Woodland Period. The barcoding genes *rbc*L, *trn*L and *mat*K are insufficiently variable to allow for phylogenetic investigations among the cultivated plants investigated here (and others that remain undetected given our arctic bait‐panel cross‐capture limitations), and as such, a larger genomic dataset of sedaDNA and cultigen reference genomes would be required to more deeply investigate ancient cultigen populations. There is also likely a greater abundance of bird sedaDNA (among many other potential organisms) than we recovered here that may have more population genomic potential from other targeted species or by also potentially enriching for nuclear DNA (Tables S10 and S11).

### 5.4 Caveats and Limitations

There are several limitations of this study that should be noted. First, the use of a regionally near‐target panel (PalaeoChip Arctic v1.0) is expected to reduce the recovery of key Eastern Woodland species (e.g., *Phaseolus*, *Zea*, *Cucurbita*) and creates unequal enrichment across the taxon pool. A regionally targeted bait‐set incorporating Eastern Woodland flora and the known archaeobotanical and archaeozoological record of southern Ontario would likely improve taxonomic coverage in future studies. Second, BLAST searches were performed against a July 2020 local database of NCBI‐NT; sequences deposited since that date—including many freshwater bacterial and algal genomes—are absent from this analysis, and some taxonomic assignments may be revised as reference databases grow. Our analysis suggests that most key organisms were sufficiently represented in our original dataset for the broad ecological analysis intended here, but that some such as *Pelolinea submarina* have much better genetic reference data post January 2021 (Table S8). Third, aDNA authentication in a massively mixed‐community metagenomic context is inherently less precise than single‐taxon genome analyses from a known fossil: damage statistics are aggregated across reads from multiple potential sources at each LCA node, and authentic signals can be diluted due to a number of biological, algorithmic and database biases. Several taxa interpreted here (e.g., *Schizophyllum*, *Phaseolus*) approach rather than comfortably exceed authentication thresholds, and their ecological interpretations should therefore be weighted accordingly. There is yet to exist an ancient environmental DNA classification pipeline that can address all of the many forms of bias inherent in classifying damaged DNA, although our ensemble approach here was selected based on successful prior experimentation to minimize false positive classifications (Murchie et al. 2026; Murchie, Kuch, et al. 2021; Murchie, Long, et al. 2023). The off‐target metagenomic classifications here should be interpreted with additional caution relative to the on‐target set as the former lacks pre‐mapping to a curated reference database designed to minimize database bias. Furthermore, the off‐target classifications had their MegaBlast alignments capped at 50 hits (MegaBlast) rather than 500 hits (BlastN) to be computationally reasonable, but which could introduce reference bias in the LCA classification. We recommend using the authenticity support to gauge confidence in ecological inferences. Off‐target taxonomic classifications may also be improved through the use of dedicated databases such as GTDB and UNITE, although our NCBI‐NT database is sufficiently comprehensive for our purposes here.

Fourth, sedaDNA taphonomy—eDNA transport by water or animals, bioturbation and differential preservation—introduces uncertainty in attributing sedaDNA signals to watershed sources. The Canada goose dung pathway documented by McAndrews and Turton (2007) is likely a dominant mechanism for plant sedaDNA input to the lake basin, but other transport vectors cannot be excluded (Moraal 2024). These unknowns in taphonomy complicate assessments of shifting abundances as well, although, as noted, we only interpret shifting abundances where there is sufficient normalization showing consistency among some organisms insofar that the shifts in others appeared ecologically meaningful given the total ecological context. Fifth, the core analysed here spans ~90 cm (approximately 1300 years) which is a fraction of the time that people lived in the area. It is likely that much longer sedaDNA records from Crawford Lake would better contextualize shifting ecosystems prior to farming in relation to deeper time human and environmental impacts on the lake ecosystem. Sixth, there is a dating ambiguity in core CL‐19 at around 40–50 cm that corresponds roughly to the second (and potentially third) occupation phase and the cessation of maize farming. As already discussed, it is unclear if this is due to a dating issue with core CL‐19 at this transition phase (Finlayson et al. 2026; Llew‐Williams et al. 2024; Moraal 2024), dating imprecision in previous chronologies at this transition (Ekdahl et al. 2004), or a delayed sedaDNA depositional signal. Our age‐depth chronology otherwise corresponds well with Ekdahl et al. (2004).

## 6 Conclusion

The ability of sedaDNA to reconstruct a diverse, high‐resolution dataset of an entire ecosystem highlights the power and utility of sedaDNA methods for improving our understanding of environmental change through time (Capo et al. 2021, 2023; Garcés‐Pastor et al. 2023; Giguet‐Covex et al. 2014, 2023; Murchie, Giguet‐Covex, et al. 2023; Orlando et al. 2021; Pedersen et al. 2015). The abundance of work carried out on this lake previously helps to validate the sedaDNA results observed here where we find a remarkable continuity in the patterns of ecological change at Crawford Lake among a diverse array of palaeoenvironmental proxy methods. However, sedaDNA not only finds the same dominant species of plants, algae and animals shifting in response to human activities over the past millennium (such as Canada goose, maize and sunflowers), but sedaDNA also complements the other proxies by identifying additional organisms whose presence was previously unknown or only assumed—such as cattle, deer, loons, beavers and many of the microbial organisms that can be investigated holistically here through their disseminated biomolecules. As ancient DNA methodologies continue to improve at all stages of processing, it is becoming increasingly clear that sedaDNA is a transformative approach for examining human–environment interactions through time. At Crawford Lake, where humans have repeatedly reshaped the landscape and ecosystems over the last millennium, this sedaDNA record helps us establish an ecological baseline to aid in further investigations for the shifts associated with the ‘Anthropocene’ concept. Crawford Lake preserves an archive of biomolecular evidence that human‐environment entanglement is ancient, cumulative, and legible across centuries of ecological succession, eutrophication, and recovery. In addition to recording an Earth System shift associated with the mid‐20th century Great Acceleration, Crawford Lake demonstrates that signals of human impact unfold across millennia and are visible in all kingdoms of life. Crawford Lake is a site of enduring scientific and cultural significance where long‐term disproportionate human impacts have become entangled with our conceptions of geologic time.

## Author Contributions

Conceptualization: T.J.M., M.K., F.M., M.F.J.P., H.N.P. Data Curation: T.J.M., M.V.E. Formal Analysis: T.J.M., M.V.E., S.L.C., L.N. Funding acquisition: L.Y.R., F.M., M.F.J.P., H.N.P. Methodology: T.J.M., M.V.E., M.K., S.L.C., L.N., M.F.J.P. Investigation: T.J.M., M.V.E., S.L.C., L.N. Visualization: T.J.M., M.V.E., S.L.C., L.N. Supervision: L.Y.R., F.M., M.F.J.P., H.N.P. Writing – original draft: T.J.M., M.V.E. Writing – review and editing: all.

## Funding

This work was funded by NSERC Alliance, Tula Foundation, CANA Foundation, Belmont Forum and BiodivERsA.

## Ethics Statement

This study analysed sedimentary ancient DNA from lake sediments and did not involve living human participants or experimental animals; therefore, formal institutional ethics approval was not required. The research was conducted in consultation with the Crawford Lake Indigenous Council, which was informed of and supported the planned sedaDNA analyses. It was agreed that any endogenous human DNA attributable to past Indigenous inhabitants would be excluded from analysis. Human DNA was not targeted in this study, and the human DNA detected was determined to represent modern contamination and was not interpreted or analysed as DNA from past Indigenous inhabitants.

## Conflicts of Interest

The authors declare no conflicts of interest.

## Supporting information


**Dataset: S1.** Characterization of the hybridization‑capture bait set. Edit distance and percent sequence divergence for the 169,972 80‑bp capture baits used in this study, relative to their aligned references, across seven animal taxa (i.e., *Alces*, *Bison*, *Bos*, *Branta*, *Gavia*, *Mammuthus*, *Odocoileus*). The workbook contains an overall summary and per‑taxon summary of bait counts and divergence statistics, the full per‑bait table (i.e., edit distance, bait length, divergence, taxon), edit distance and divergence distributions, pairwise comparisons quantifying baits shared between taxa and their relative divergence and individual bait tables for representative taxa.

**Dataset: S2.** Capture enrichment performance and bait conservation. Comparison of shotgun versus hybridization‑enriched sequencing to quantify target enrichment. Includes overall enrichment statistics, such as per‑taxon (baitset; *n* = 756) and per‑reference (FullRefs; *n* = 3543) shotgun coverage, enriched coverage and fold enrichment; enrichment breakdowns by category (mutually exclusive and cumulative), shotgun‑coverage distribution, sequence‑conservation analyses of baits shared among taxon pairs (shared vs. unique divergence) and sample‑level read enrichment summaries (total and on‑target reads before vs. after capture).

**Dataset: S3.** Read‑level mapping and divergence of enriched sequences. Edit distance, percent divergence and read length for enriched reads mapped both to the bait set (Baitset; 1,402,036 reads across 757 taxa) and to the full reference database (FullRefs; 4,218,307 reads across 3530 references). The workbook provides overall and per‑taxon/per‑reference summaries, edit‑distance, divergence and cumulative distributions, the Figure 4 comparison of read versus bait divergence and 100,000‑read subsamples of the underlying per‑read data. Roughly 99% of reads fall below 10% divergence.

**Dataset: S4.** Ensemble sedaDNA support using combined *BLASTn*/*MEGAN* and *Holi* classifications. Both on‐ and off‐target classifications are reported with full associated classification metadata from both approaches, as well as summary sheets showing condensed important metadata and classification support metrics.

**Data S1:** mec70529‐sup‐0005‐DataS1.docx.

**Figure S1:** Shifting sedaDNA abundance through time. Top 10 most abundant plant/animal taxonomic assignments. Settlement phases assumed based on two/three main occupation periods and their close spatial‐temporal proximity, although their direct correlation is unconfirmed.
**Figure S2:** Stacked bar chart of ‘golden’ algae. Normalized read counts to the smallest library.
**Figure S3:** MtDNA phylogenetics of *B. canadensis* sedaDNA. (A) Maximum likelihood tree of publicly available *B. canadensis* COX1 and (B) control region sequences.
**Figure S4:** Chloroplast assemblies, phylogenetics and damage patterns of *Zea mays* (maize/corn) sedaDNA. (A) Maximum likelihood tree of publicly available rbcL genes. (B) *mapDamage* fragment length distributions and (C) deamination rates of sedaDNA assemblies.
**Figure S5:** Chloroplast assemblies, phylogenetics and damage patterns of *Helianthus annuus* (common sunflower) sedaDNA. (A) Maximum likelihood tree of publicly available *rbc*L *Helianthus* sequences. (B) *mapDamage* fragment length distributions and (C) deamination rates of sedaDNA assemblies. The bimodal fragment lengths in the FLD are primarily driven by off‐target mapping of related taxa in homologous regions. The rbcL assemblies were competitively mapped to remove this off‐target signal and polymorphisms in regions of read stacking were N‐masked.
**Figure S6:** Chloroplast assemblies, phylogenetics and damage patterns of Pinaceae sedaDNA. (A) Maximum likelihood tree of publicly available rbcL genes. (B) *mapDamage* fragment length distributions and (C) deamination rates of sedaDNA assemblies.
**Figure S7:** metaDMG plots of damage versus significance for top abundance taxonomic families. (A) All libraries merged and (B) Libraries merged by ecological zone.
**Figure S8:** Bait‐to‐reference edit distances. (A) Mean edit distance by taxon, (B) distribution of plants and animals and (C) distribution by taxon.
**Figure S9:** SedaDNA divergences. (A) Read‐to‐bait and (B) read‐to‐reference with cumulative distributions.
**Figure S10:** Edit distance, divergence and read length with reads and baits to references. (A) Edit distance and divergence of sedaDNA reads and baits relative to the references. (B) Read length versus divergence.
**Figure S11:** Reference similarity for evaluating bait cross‐capture. (A) Conservation and divergence among animal taxa and (B) similarity and divergence with the 11 tested plant/animal references.
**Figure S12:** Animal bait mapping to select reference genomes.
**Figure S13:** Plant bait mapping to select reference genomes.
**Figure S14:** Age‐depth model for core CL‐19.
**Figure S15:** Age‐depth model re‐analysing the data from Ekdahl et al. (2004) with IntCal20.
**Figure S16:** MetaMerge heatmap of plant and algal sedaDNA from the on‐target analysis with conservative MEGAN LCA hits merged with Holi/metaDMG damage support. Page 1 of 5.
**Figure S17:** As Figure S16, but cell colour indicates the metaDMG maximum‐likelihood C→T damage estimate (δs) per taxon–library combination. Higher values (darker) reflect greater deamination‐associated aDNA damage. Page 1 of 5.
**Figure S18:** Stacked bar chart of plant, algal and animal sedaDNA read‐count proportions across Crawford Lake depth intervals from the on‐target analysis.
**Figure S19:** MetaMerge heatmap of animal sedaDNA detected in Crawford Lake sediment core, on‐target analysis.
**Figure S20:** As Figure S19, but cell colour indicates metaDMG C→T damage estimate (δs).
**Figure S21:** Stacked bar chart of animal sedaDNA read‐count proportions across Crawford Lake depth intervals (on‐target analysis).
**Figure S22:** MetaMerge heatmap of plant and algal sedaDNA detected using off‐target MegaBLAST analysis (40 individual enriched and shotgun libraries merged by biosample). Read counts are normalized; cells displaying 0 represent 0–1 normalized reads.
**Figure S23:** As Figure S22, but cell colour indicates metaDMG C→T damage estimate (δs).
**Figure S24:** Stacked bar chart of plant, algal and animal sedaDNA read‐count proportions across Crawford Lake libraries (off‐target analysis).
**Figure S25:** MetaMerge heatmap of animal sedaDNA detected using off‐target MegaBLAST analysis. Read counts are normalized; cells displaying 0 represent 0–1 normalized reads.
**Figure S26:** As Figure S25, but cell colour indicates metaDMG C→T damage estimate (δs).
**Figure S27:** Stacked bar chart of animal sedaDNA read‐count proportions across Crawford Lake libraries (off‐target analysis).
**Figure S28:** MetaMerge heatmap of fungal sedaDNA detected in Crawford Lake sediment core (off‐target MegaBLAST analysis). Read counts are normalized; cells displaying 0 represent 0–1 normalized reads.
**Figure S29:** As Figure S28, but cell colour indicates metaDMG C→T damage estimate (δs).
**Figure S30:** Stacked bar chart of fungal sedaDNA read‐count proportions across Crawford Lake libraries (off‐target analysis).
**Figure S31:** MetaMerge heatmap of bacterial and archaeal sedaDNA detected in Crawford Lake sediment core (off‐target MegaBLAST analysis). Read counts are normalized; cells displaying 0 represent 0–1 normalized reads.
**Figure S32:** As Figure S31, but cell colour indicates metaDMG C→T damage estimate (δs).
**Figure S33:** Stacked bar chart of bacterial and archaeal sedaDNA read‐count proportions across Crawford Lake libraries (off‐target analysis).
**Figure S34:** CONISS cluster dendrogram for Crawford Lake plant, algal, and animal sedaDNA assemblages across 28 depth intervals.
**Figure S35:** Stratigraphic diagram of top 20 damage supported ecologically informative plant, algal and animal sedaDNA read‐count proportions across depth intervals (on‐target analysis), with CONISS zones.
**Figure S36:** Stratigraphic diagram of top 20 damage supported ecologically informative bacterial, archaeal, algal (off‐target) and fungal sedaDNA read‐count proportions across depth intervals (off‐target analysis), with CONISS zones.
**Figure S37:** Stratigraphic diagram of top 20 damage supported ecologically informative taxa from the on/off‐target analyses combined (equal weighting between the two) with CONISS zones.
**Figure S38:** metaDMG damage versus significance plots for prokaryotic taxa across Crawford Lake sediment libraries (sample‐faceted). Each panel represents one sequencing library. *x*‐axis: maximum‐likelihood C→T damage estimate (δs); *y*‐axis: statistical significance.
**Figure S39:** As Figure S38, but libraries grouped and colour‐coded by stratigraphic era.
**Figure S40:** Top prokaryotic genera detected across Crawford Lake sediment libraries by metaDMG‐authenticated read proportions.
**Figure S41:** As Figure S40, but libraries grouped and colour‐coded by stratigraphic era.
**Figure S42:** Estimated internal alignment identity and C to T damage for ecologically interpreted microbial, algal and fungal taxa. Left panel: estimated internal alignment identity (= 1 − background C to T rate − non‐CT/GA mismatch rate), derived from the metaDMG substitution frequency model. This metric quantifies per‐position read‐to‐reference identity at internal read positions. Vertical dashed lines indicate the 95% identity threshold applied during MEGAN LCA classification (red) and the 97% threshold raised by reviewer 1 (orange). Right panel: 5′‐terminal C to T damage amplitude (δs); stars indicate damage significance ≥ 2.0. Taxa are grouped by broad category (coloured left stripe) and coloured by aDNA confidence tier. Taxa with Tentative classification (orange) are included because they were ecologically discussed in the main text; their confidence reflects incomplete damage authentication rather than low alignment identity. All values are from the best‐matched Holi/metaDMG library for each taxon.
**Figure S43:** Internal alignment identity for ecologically interpreted microbial, algal and fungal taxa. Left panel: internal alignment identity derived from the metaDMG substitution frequency model. This metric quantifies per‐position read‐to‐reference identity at internal read positions. Vertical dashed lines indicate the 95% identity threshold applied during MEGAN LCA classification (red) and the 97% threshold raised by reviewer 1 (orange). Right panel: 5′‐terminal C to T damage amplitude (δs); stars indicate damage significance ≥ 2.0. Taxa are grouped by broad category (coloured left stripe) and coloured by aDNA confidence tier. Taxa with Tentative classification (orange) are included because they were ecologically discussed in the main text; their confidence reflects incomplete damage authentication rather than low alignment identity. All values are from the best‐matched Holi/metaDMG library for each taxon.
**Figure S44:** Fragment length distribution example of reads from MEGAN7 assigned taxonomic nodes. Plots demonstrate that reads < 30 bp are not assigned in this dataset despite the min 24 bp floor during initial processing. Only reads of a sufficient length accumulate the necessary metrics for LCA assignment. Part 1 of 2.
**Figure S45:** PCR duplication between rounds of hybridization capture.
**Figure S46:** Challenges of nuclear lake sedaDNA, initial explorations.
**Table S1:** EAGER summary statistics for human mapped (rCRS) shotgun sequenced libraries.
**Table S2:** EAGER summary statistics for human mapped enriched libraries batch 1.
**Table S3:** EAGER summary statistics for human mapped enriched libraries batch 2.
**Table S4:** Chloroplast gene coordinates for 6 plant taxa.
**Table S5:** Age‐depth model data for Figure S14.
**Table S6:** Age estimate by depth (*z*) for core CL‐19.
**Table S7:** CONISS zonation summary for Crawford Lake sedaDNA assemblage.
**Table S8:** NCBI‐NT reference database coverage for key interpreted taxa at time of analysis (2020) versus current (June 2026). Pre‐2021 sequences: count of sequences with accession date before 2021‐01‐01 in NCBI‐NT. Post‐2020 sequences: new sequences added after 2020.
**Table S9:** Calculated BLAST floor. Minimum read length at which BLAST reports any alignment at all and that alignment's bit score reaches MEGAN's *MinScore* = 50 threshold, by BLAST task, mismatch placement scenario and target percent identity:
**Table S10:** Branta canadensis mtDNA vs nuclear, shotgun vs capture. Unique reads, MQ ≥ 30. Includes 0‐round (shotgun), 1‐round (C#/CL#) and 2‐round (CLO‐#) capture. Nuclear reads are diverse (low duplication), short and low‐identity.
**Table S11:** Nuclear vs mtDNA Branta competitive test. Reads mapping to the *Branta* nuclear genome vs a distant‐bird decoy (chicken, Gallus gallus GCF_016699485.2).

## Data Availability

Raw sequence data have been uploaded to the NCBI SRA (BioProject PRJNA1294983). All data needed to evaluate the results presented here are included as supplementary text, figures, tables and datafiles Processed files derived from the raw sequence data will be stored on servers at the Hakai Institute and be available upon request to Tyler Murchie (tyler.murchie@hakai.org). The bait sequences used for capture enrichment are located here: https://doi.org/10.5281/zenodo.5643845.
