# Supplementary material for: Sedimentary Ancient DNA Tracks Multi‐Kingdom Ecosystem Reorganizations Following Sequential Human Land Use at Crawford Lake
Source: Mol Ecol. 2026 Sep 8;35(17):e70529. doi: 10.1111/mec.70529 (PMC13551257; doi:10.1111/mec.70529)
Supplement: Supplementary file 1 [file MEC-35-e70529-s003.docx]

**Supplementary Dataset Captions**

**Dataset-S1.xlsx**

**Characterization of the hybridization‑capture bait set.** Edit distance and percent sequence divergence for the 169,972 80‑bp capture baits used in this study, relative to their aligned references, across seven animal taxa (i.e., *Alces*, *Bison*, *Bos*, *Branta*, *Gavia*, *Mammuthus*, *Odocoileus*). The workbook contains an overall summary and per‑taxon summary of bait counts and divergence statistics, the full per‑bait table (i.e., edit distance, bait length, divergence, taxon), edit distance and divergence distributions, pairwise comparisons quantifying baits shared between taxa and their relative divergence, and individual bait tables for representative taxa.

**Dataset-S2.xlsx**

**Capture enrichment performance and bait conservation.** Comparison of shotgun versus hybridization‑enriched sequencing to quantify target enrichment. Includes overall enrichment statistics, such as per‑taxon (baitset; n = 756) and per‑reference (FullRefs; n = 3,543) shotgun coverage, enriched coverage, and fold enrichment; enrichment breakdowns by category (mutually exclusive and cumulative), shotgun‑coverage distribution, sequence‑conservation analyses of baits shared among taxon pairs (shared vs. unique divergence), and sample‑level read enrichment summaries (total and on‑target reads before vs. after capture).

**Dataset-S3.xlsx**

**Read‑level mapping and divergence of enriched sequences.** Edit distance, percent divergence, and read length for enriched reads mapped both to the bait set (Baitset; 1,402,036 reads across 757 taxa) and to the full reference database (FullRefs; 4,218,307 reads across 3,530 references). The workbook provides overall and per‑taxon/per‑reference summaries, edit‑distance, divergence, and cumulative distributions, the Figure 4 comparison of read versus bait divergence, and 100,000‑read subsamples of the underlying per‑read data. Roughly 99% of reads fall below 10% divergence.

**Dataset-S4.xlsx**

**Ensemble sedaDNA support using combined *BLASTn*/*MEGAN* and *Holi* classifications.** Both on- and off-target classifications are reported with full associated classification metadata from both approaches, as well as summary sheets showing condensed important metadata and classification support metrics.
