# Supplementary material for: Sedimentary Ancient DNA Tracks Multi‐Kingdom Ecosystem Reorganizations Following Sequential Human Land Use at Crawford Lake
Source: Mol Ecol. 2026 Sep 8;35(17):e70529. doi: 10.1111/mec.70529 (PMC13551257; doi:10.1111/mec.70529)
Supplement: Supplementary file 2 [file MEC-35-e70529-s002.docx]

Supplementary Materials for

**Sedimentary ancient DNA tracks multi-kingdom ecosystem reorganizations following sequential human land use at Crawford Lake**

Murchie and Emery *et al.*

*Corresponding authors:

Tyler J. Murchie: tyler.murchie@hakai.org,

Matthew V. Emery: memery@binghamton.edu,

Francine McCarthy: fmccarthy@brocku.ca,

Michael F.J. Pisaric: mpisaric@brocku.ca,

Hendrik N. Poinar: poinarh@mcmaster.ca

**This PDF file includes:**

Supplementary text

Figs. S1 to S46

Tables S1 to S11

**Table of Contents**

**Supplementary Text**	**1**

Extended Methods	4

Modern human DNA methods and results	11

Additional bait benchmarking results	11

Comparative Fold-Enrichment	11

Shotgun Coverage Distribution and Rare Taxa	12

Sequence Conservation	12

Ancient DNA Read-to-Bait Divergence Results	13

Ancient DNA Read-to-Bait Divergence Discussion	13

BLAST length/score floor calculations	14

Nuclear DNA	15

**Supplementary Figures**	**17**

Fig. S1 Shifting sedaDNA abundance through time.	17

Fig. S2 Stacked bar chart of “golden” algae	18

Fig. S3 MtDNA phylogenetics of B. canadensis sedaDNA	19

Fig. S4 Chloroplast assemblies, phylogenetics, and damage patterns of Zea mays (maize/corn) sedaDNA	20

Fig. S5 Chloroplast assemblies, phylogenetics, and damage patterns of Helianthus annuus (common sunflower) sedaDNA	21

Fig. S6 Chloroplast assemblies, phylogenetics, and damage patterns of Pinaceae sedaDNA	22

Fig. S7 metaDMG plots of damage versus significance for top abundance families	23

Fig. S8 Bait-to-reference edit distances	24

Fig. S9 SedaDNA divergences	25

Fig. S10 Edit distance, divergence, and read length with reads and baits to references	26

Fig. S11 Reference similarity for evaluating bait cross-capture.	27

Fig. S12 Animal bait mapping to select reference genomes.	28

Fig. S13 Plant bait mapping to select reference genomes	29

Fig. S14 Age-depth model for core CL-19	30

Fig. S15 Age-depth model re-analyzing the data from Ekdahl et al. (2004) with IntCal20	31

Fig. S16 MetaMerge heatmap of plant and algal sedaDNA from the on-target analysis with conservative MEGAN LCA hits merged with Holi/metaDMG damage support.	32

Fig. S17 As Fig. S16, but cell colour indicates the metaDMG maximum-likelihood C→T damage estimate (δs) per taxon–library combination.	37

Fig. S18 Stacked bar chart of plant, algal, and animal sedaDNA read-count proportions across Crawford Lake depth intervals from the on-target analysis.	42

Fig. S19 MetaMerge heatmap of animal sedaDNA detected in Crawford Lake sediment core, on-target analysis.	43

Fig. S20 As Fig. S19, but cell colour indicates metaDMG C→T damage estimate (δs).	44

Fig. S21 Stacked bar chart of animal sedaDNA read-count proportions across Crawford Lake depth intervals (on-target analysis).	45

Fig. S22 MetaMerge heatmap of plant and algal sedaDNA detected using off-target MegaBLAST analysis	46

Fig. S23 As Fig. S22, but cell colour indicates metaDMG C→T damage estimate (δs).	99

Fig. S24 Stacked bar chart of plant, algal, and animal sedaDNA read-count proportions across Crawford Lake libraries (off-target analysis).	152

Fig. S25 MetaMerge heatmap of animal sedaDNA detected using off-target MegaBLAST analysis.	153

Fig. S26 As Fig. S25, but cell colour indicates metaDMG C→T damage estimate (δs).	165

Fig. S27 Stacked bar chart of animal sedaDNA read-count proportions across Crawford Lake libraries (off-target analysis).	177

Fig. S28 MetaMerge heatmap of fungal sedaDNA detected in Crawford Lake sediment core (off-target MegaBLAST analysis).	178

Fig. S29 As Fig. S28, but cell colour indicates metaDMG C→T damage estimate (δs).	180

Fig. S30 Stacked bar chart of fungal sedaDNA read-count proportions across Crawford Lake libraries (off-target analysis)	182

Fig. S31 MetaMerge heatmap of bacterial and archaeal sedaDNA detected in Crawford Lake sediment core (off-target MegaBLAST analysis).	183

Fig. S32 As Fig. S31, but cell colour indicates metaDMG C→T damage estimate (δs).	220

Fig. S33 Stacked bar chart of bacterial and archaeal sedaDNA read-count proportions across Crawford Lake libraries (off-target analysis).	257

Fig. S34 CONISS cluster dendrogram for Crawford Lake plant, algal, and animal sedaDNA assemblages across 28 depth intervals	258

Fig. S35 Stratigraphic diagram of top 20 damage supported ecologically informative plant, algal, and animal sedaDNA read-count proportions across depth intervals (on-target analysis), with CONISS zones.	259

Fig. S36 Stratigraphic diagram of top 20 damage supported ecologically informative bacterial, archaeal, algal (off-target), and fungal sedaDNA read-count proportions across depth intervals (off-target analysis), with CONISS zones.	260

Fig. S37 Stratigraphic diagram of top 20 damage supported ecologically informative taxa from the on/off-target analyses combined with CONISS zones	261

Fig. S38 metaDMG damage versus significance plots for prokaryotic taxa across Crawford Lake sediment libraries (sample-faceted).	262

Fig. S39 As Fig. S36, but libraries grouped and colour-coded by stratigraphic era.	263

Fig. S40 Top prokaryotic genera detected across Crawford Lake sediment libraries by metaDMG-authenticated read proportions	264

Fig. S41 As Fig. S38, but libraries grouped and colour-coded by stratigraphic era	265

Fig. S42 Estimated internal alignment identity and C to T damage for ecologically interpreted microbial, algal, and fungal taxa	266

Fig. S43 Internal alignment identity for ecologically interpreted microbial, algal, and fungal taxa.	267

Fig. S44 Fragment length distribution example of reads from MEGAN7 assigned taxonomic nodes	268

Fig. S45 PCR duplication between rounds of hybridization capture.	270

Fig. S46 Challenges of nuclear lake sedaDNA, initial explorations.	270

**Supplementary Tables**	**271**

Table S1 EAGER summary statistics for human mapped (rCRS) shotgun libraries.	271

Table S2 EAGER summary statistics for human mapped (rCRS) enriched libraries batch 1	272

Table S3 EAGER summary statistics for human mapped (rCRS) enriched libraries batch 2	273

Table S4 Chloroplast gene coordinates for 6 plant taxa.	274

Table S5 Age-depth model data for Fig. S14.	275

Table S6 Age estimate by depth (z) for core CL-19.	276

Table S7 CONISS zonation summary for Crawford Lake sedaDNA assemblage	279

Table S8 NCBI-NT reference database coverage for key interpreted taxa, 2020/2026	280

Table S9 Calculated BLAST floor.	281

Table S10 Branta canadensis mtDNA vs nuclear, shotgun vs capture.	282

Table S11 Nuclear vs mtDNA Branta competitive test.	283

**Supplementary Dataset Captions**	**283**

**Supplementary References**	**284**

**Supplementary Text**

Extended Methods

*Extraction*

Lysis followed the extraction procedures described in Murchie et al. (Murchie et al., 2022, 2023, 2026; Murchie, Kuch, et al., 2021; Murchie, Monteath, et al., 2021) utilizing an overnight proteinase K digestion buffer (Tris-Cl, SDS, Proteinase K, CaCl_2_, DTT, PTB, PVP) and PowerBead Pro bead beating with a TissueLyser (Qiagen), and with sediment inputs of ~0.15–0.17 grams (wet weight). Samples were digested overnight at 35ºC then centrifuged the following morning (~18 hours later) so that the lysis supernatant could be removed (~1 mL). Purifications followed the Murchie et al. (2021) sedaDNA modified purification protocol using high volume silica columns (Roche High-Pure Viral columns) with a high concentration guanidine binding buffer and the cold spin inhibitor removal approach (Dabney et al., 2013; Karpinski et al., 2016; Murchie, Kuch, et al., 2021). The cold spin inhibitor removal used a speed of 2500 rcf (at 4ºC) for ~20 hours. Extracts were eluted in 50 μL of EBT (25 μL twice).

*Library Preparation*

Double stranded libraries were prepared as described in Meyer and Kircher (2010) with modifications from Kircher et al. (2012) following stages for blunt end repair (without UDG treatment to retain aDNA characteristic deamination patterns), adapter ligation, and adapter fill-in. Following blunt end repair, the QiaQuick Nucleotide Removal Kit (Qiagen) was used to maximize the recovery of small fragments (recovers fragments as low as ~40 bp), performing 2 PE washes and spinning at 3800 rcf for all steps prior to the dry spin and elution (carried out at maximum speed). After overnight adapter ligation for >16 hours, the MinElute PCR Purification kit (Qiagen) was used as fragments with ligated adapters now exceeded the minimum fragment recovery (~70 bp). Enzymes were heat-inactivated following adapter fill-in to reduce the need for another purification (silica purifications have been generally observed to result in ~50% loss of input DNA [data not shown]).

Dual-unique 8-bp indexes were used for quantitative PCR (qPCR) fluorescence guided amplification (Murchie, Kuch, et al., 2021) where libraries are removed just prior to amplification plateau to minimize the creation of heteroduplexes (DNA with a mix of single- and double-stranded regions) (Gansauge et al., 2020). Following indexing, libraries were purified with the MinElute PCR Purification kit following manufacturer specifications and eluted in 15 uL of EBT.

*Enrichment*

Indexed libraries were processed with targeted capture enrichment via RNA hybridization with the PalaeoChip Arctic v1.0 bait-set (Murchie, Kuch, et al., 2021). This panel is designed to target whole mitochondrial genomes (~16,000 base pairs [bp]) of ~180 extinct and extant Holarctic fauna, along with the chloroplast genes *trn*L, *rbc*L, and *mat*K (total ~2000 bp per species) from ~2100 species of Holarctic plants. It was designed using available reference genomes on NCBI in 2017 with an emphasis on late Quaternary Arctic/Subarctic animals and plants. Its use here for capturing sedaDNA from a late Holocene archaeological site in the Eastern Woodlands is within the overall scope of the expected ecological diversity targeted with the panel. However, this enrichment panel was not designed with this regional context in mind, and as such, several important agricultural taxa specific to the Great Lakes region (and further south) were not directly targeted here.

Targeted capture was used for both extraction batches, but with several differences. Batch 1 was processed with the myBaits v4.1 protocol using a single round of enrichment (200 ng of baits per reaction), hybridizing at 60ºC for 48 hours. The second batch of extracts was processed with the myBaits v5 protocol with two rounds of enrichment (275 ng and 150 ng of baits per reaction, respectively) using a single overnight hybridization per round at 58ºC.

Capture conditions differed between the two processing batches because the samples were enriched at different times as the laboratory protocol evolved. These differences are reported as methodological metadata and should not be interpreted as a controlled comparison of hybridization temperature or number of enrichment rounds. In particular, the one-round and two-round enrichment strategies were not applied to matched aliquots of the same extracts under otherwise identical conditions. As a result, differences in read recovery may reflect batch, extract, library, sequencing-depth, bait-target divergence, or sample-preservation effects rather than enrichment-round number alone. We did find here (and have found in other parallel efforts) that two rounds of enrichment does not improve outcomes for large ecological bait-sets sufficiently to justify the large additional cost, and results in a large number of PCR duplicates (Fig. S45).

*Sequencing*

Libraries were pooled to roughly equimolar concentrations using qPCR and size-selected with gel excision following electrophoresis for molecules ranging between 150–500 bp. Libraries in batch 1 were sequenced on an Illumina HiSeq 1500 with a 2x75 paired-end protocol while libraries from batch 2 were sequenced on an Illumina NextSeq 2000 with a 2x50 paired end kit. Both were sequenced at the Farncombe Metagenomics Facility (McMaster University, ON). Sequenced reads were demultiplexed with Illumina *DRAGEN* secondary analysis software (BCL-Convert v4.3.6).

*Metagenomic classification methods*

Stage 1, demultiplexed *FASTQ* files were trimmed and merged with *leeHom* (Renaud et al., 2014) using ancient DNA parameters (--ancientdna) then mapped (map-filtered) to the PalaeoChip Arctic 1.0 reference *FASTAs* with *network-aware-BWA* (<https://github.com/mpieva/network-aware-bwa>, maximum edit distance of 0.01 [-n 0.01], allowing for a maximum of two gap openings [-o 2], and with seeding effectively disabled [-l 16500]). All reads that mapped (merged or unmerged but properly paired) were then extracted with *libbam* (<https://github.com/grenaud/libbam>), deduplicated based on 5’ and 3’ mapping positions with *biohazard* (<https://bitbucket.org/ustenzel/biohazard>), and converted to *FASTA* files and size restricted to a minimum length of 24 bp. These map-filtered *FASTA* read files were filtered to remove any lingering sequence similarity to the Illumina adapter sequences (sequences with an edit distance of 1 to the common adapter sequence AGATCGGAA and its reverse complement), and were then string deduplicated using the *NGSXRemoveDuplicates* module of *NGSeXplore* (<https://github.com/ktmeaton/NGSeXplore>).

Stage 2, map and quality filtered *FASTAs* were used as the input for *BLASTn* (Altschul et al., 1990), which were aligned against a 2020 local copy of *NCBI-NT* set to return the top 500 alignments (unique accession hits) per read with e-values less than 1.0E-5 (flags: -num_alignments 500 -max_hsps 1 -evalue 0.00001).

Stage 3, *BLASTn* output files were then passed to *MEGAN7* (v7.0.9-beta) where the *BLASTn* results were filtered through a lowest common ancestor (LCA) algorithm using the following parameters: Min-score = 50, Max expected (e-value) = 1.0E-5, Minimum percent identity = 95%, Top percent consideration of taxonomic hits based on bit-score = 10%, Minimum read support = 3, naive LCA at 100% coverage, Minimum percent read to cover = 90%. Replicate depth libraries were merged in *MEGAN* using the absolute comparison tool. All libraries (merged replicates and single libraries processed without replicates) were compared using the absolute comparison tool.

The *MEGAN7* counts were extracted and merged with the *Holi*/*metaDMG* classifications (as described in the main text) using *MetaMerge* to generate heat maps of counts with authenticity support, deamination percentages, and stacked bar plots of top taxa identified (<https://github.com/tjmurchie/TackleBox/tree/main/MetaMerge>).

*Effective minimum read length after taxonomic classification*

Reads were retained down to a permissive 24 bp minimum length prior to alignment to avoid discarding genuinely short ancient DNA fragments *a priori*. However, the LCA parameters used for *MEGAN7* taxonomic binning (MinScore = 50.0, MinPercentIdentity = 95.0; off-target: MegaBLAST/-task megablast; on-target: BlastN/-task blastn) impose a considerably higher effective minimum length on reads that actually receive a taxonomic assignment. We quantified this directly, both theoretically and empirically. Using real BLAST+ (v2.17.0) on synthetic reads of controlled length and percent identity, the minimum read length able to reach a bit score of 50 is 27 bp (blastn defaults, 100% identity) to 30 bp (blastn defaults, 95% identity), and 28 bp (megablast defaults, 100% identity, additionally seed-limited by MegaBLAST's default 28 bp word size) to 34 bp (megablast defaults, 95% identity). Consistent with this, we found that across every sample and taxon in both datasets, the shortest read receiving a taxonomic assignment was 31 bp (on-target, n = 159,633 assigned reads) or 30 bp (off-target, n = 1,786,252 assigned reads). Zero reads in the nominal 24–29 bp retention window were ever taxonomically assigned.

Example fragment length distributions for the taxa discussed in the main text are shown in Fig. S44, and per-taxon length-bin statistics are provided in supplementary Dataset-4. Code to reproduce this analysis (extract_rma6_flds.py, build_key_taxa_report.py, verify_length_score_relationship.py) is provided as supplementary code.

*Single-target genome assembly*

Mitochondrial genomes for *Branta* and chloroplast genes for plants were assembled with the following reference mapping approach. Demultiplexed *FASTQ* files were converted to *BAM* files with *fastq2bam* (<https://github.com/grenaud/BCL2BAM2FASTQ>), then trimmed and merged with *leeHom* (Renaud et al., 2014) using ancient DNA specific parameters (--ancientdna). Reads were mapped to a reference with network-aware-BWA (<https://github.com/mpieva/network-aware-bwa>) (Li & Durbin, 2009) with a maximum edit distance of 0.01 (-n 0.01), allowing for a maximum two gap opening (-o 2), and with seeding effectively disabled (-l 16500). For *Branta*, reads were directly mapped to the *B. canadensis* NCBI RefSeq (NC_007011). For the plant taxa, reads were mapped to several species simultaneously using a competitive mapping approach to improve on-target mapping quality due to conservation of the targeted genes. Reads were mapped to *Helianthus annuus* (NC_007977, common sunflower), *Phaseolus vulgaris* (NC_009259, common bean), *Cucurbita pepo* (NC_038229, squash), *Zea mays* (NC_001666, maize/corn), and *Pinus contorta* (NC_011153, eastern white pine). For damage comparisons, *Branta* mapped reads were compared against permafrost bone and sediment libraries (Murchie et al., 2022; Murchie, Monteath, et al., 2021; Rowe et al., 2024) mapped to *Mammuthus primigenius* (NC_007596.2) to assess the relative rates of deamination and depurination between lake sediments and permafrost-preserved terrestrial sediment and bone.

Mapped reads that were merged or unmerged but properly paired were extracted with *libbam* (<https://github.com/grenaud/libbam>), collapsed based on unique 5’ and 3’ positions with *biohazard* (<https://bitbucket.org/ustenzel/biohazard>) (for PCR deduplication), and filtered to only retain reads with a >30 map quality and minimum fragment length of 24 bp using *samtools* (<https://github.com/samtools/samtools>). These mapped bam files were then imported into Geneious Prime 2025.0a (<https://www.geneious.com/>) for visualization where the contigs were manually curated using pre-assembled alignments and NCBI references as guides to reduce the influence of stacked, non-specific reads (common in both aDNA and eDNA libraries) from impacting consensus calls of false positive single nucleotide polymorphisms and indels. Spurious-indels were removed, nucleotides with a read coverage less than 3 were ‘N’ masked, and regions with high coverage (≥3 standard deviations from the coverage mean) were carefully inspected for non-specific mapping, and all polymorphisms relative to the reference in and adjacent to these high coverage regions were N-masked. Consensus sequences were called with an 75% nucleotide identity threshold and degenerate bases called in ambiguous positions. Mitogenome and chloroplast alignments were curated for sequences with ≳ 80% completeness and >3X coverage. The new consensus sequences were added and aligned with *muscle* (v3.8.425) (Edgar, 2004). The alignments were inspected, and end gaps were replaced with Ns. Model testing and maximum likelihood analyses were conducted using *IQ-Tree* (v1.6.1283) with outgroup rooting and bootstrap replicates (indicated on associated trees).

*Evaluating hybridization enrichment with a ‘near-target’ panel*

*1) Bait-to-Reference Divergence.* For animal taxa, we used seven complete mitochondrial genome references representing species either targeted in the PalaeoChip Arctic v1.0 baits (Murchie, Kuch, et al., 2021) or Crawford Lake sediments. These included *Alces alces* (moose), *Bison bison* (American bison), *Bos taurus* (cattle), *Branta canadensis* (Canada goose), *Gavia immer* (common loon), *Mammuthus primigenius* (woolly mammoth), and *Odocoileus virginianus* (white-tailed deer). All mitochondrial genomes were obtained as complete *FASTA* files, with one file per taxon. For plant taxa, we used six complete chloroplast genome references representing *Artemisia*, *Cucurbita*, *Helianthus*, *Plantago*, *Phaseolus*, and *Zea*. Because PalaeoChip Arctic 1.0 plant baits specifically target three genes (*rbc*L, *mat*K, and *trn*L) rather than entire chloroplast genomes, we restricted our analysis to these gene regions using a *BED* file with the start and end position flanking the target genes.

To restrict plant bait-to-reference divergence analyses to target gene regions, we extracted species-specific coordinates for *rbc*L, *mat*K, and *trn*L-UAA genes from NCBI GenBank chloroplast genome annotations (Table S4). Complete chloroplast genome sequences were accessed via the NCBI E-utilities API using the following accessions: NC_001666 (*Zea mays,* corn/maize), NC_020607 (*Artemisia frigida, Prairie Sagebrush*), NC_038239 (*Cucurbita pepo,* squash/pumpkin), NC_007977 (*Helianthus annuus,* common sunflower), NC_084063 (*Plantago major,* Broadleaf plantain), and NC_009259 (*Phaseolus vulgaris,* common bean). Gene feature annotations were parsed from GenBank format files to identify exact genomic coordinates for each target gene. We specifically selected the trnL-UAA gene (the large ~500-600 bp intron-containing tRNA-Leu variant) rather than smaller trnL variants, as the PalaeoChip Arctic 1.0 bait sequences target the intron-containing form. GenBank coordinates (1-based, inclusive) were converted to *BED* format (0-based start, 1-based end) for use with samtools view -L to restrict analysis to these three chloroplast gene regions.

Baits representing all 13 taxa were aligned to their respective reference genomes using *BWA-aln*. Following alignment, unmapped baits were removed, and the remaining mapped baits were sorted and indexed using *SAMtools*. We used the *SAMtools calmd* function to compute per-base mismatch by comparing the aligned sequence to the reference (*NM* tag to each aligned bait). We then extracted these *NM* tags using *AWK* scripting to parse the *BAM* files.

For animal taxa, we processed whole mitochondrial genomes, and for plants, we extracted the *rbc*L, *mat*K, and *trn*L gene regions via *BED* file with *SAMtools*, which created BAM files containing only bait alignments within these three target regions. We calculated divergence percentage for each aligned bait using the following formula: Divergence (%) = (Edit Distance / Bait Length) x 100, where Edit Distance is the *NM* tag value and Bait Length is 80 bp for all baits.

For each taxon, we aggregated all individual bait divergence measurements and calculated summary statistics using Python’s *pandas* library. We computed the number of baits successfully mapped to each reference, mean edit distance, mean divergence and median divergence percentages per taxon.

*2) Shotgun versus capture fold enrichment*. We quantified enrichment efficiency by comparing taxonomic read abundance in shotgun metagenomics libraries versus PalaeoChip Arctic-enriched libraries with two approaches: direct mapping of fold enrichment by taxon and *BLASTn*/*MEGAN7* *LCA* assignment of organelle sedaDNA.

*Mapped enrichment by taxon:* we analyzed paired-end Illumina sequencing data from both shotgun libraries and PalaeoChip Arctic-enriched libraries. Adapters were trimmed and merged using AdapterRemoval v2.3.2 with the following aDNA-appropriate parameters: --trimns and --trimqualities to remove terminal low-confidence calls, --minquality 30 (Phred score of ≥30; ≤0.1% error rate), and removed reads less than 24 bp in length (--minlength 24). After trimming and collapsing, we retained >97% of reads per library with mean post-trimming fragment lengths of approximately 40 bp, consistent with expected aeDNA fragment lengths. We aligned the reads to two different reference sets: the bait-set analysis used 80 bp bait sequences as the reference, while the "FullRefs" analysis used the original whole mtDNAs and chloroplast genes (*mat*K, *rbc*L, and *trn*L). Taxonomic identities were assigned to reads based on which bait sequence they aligned to. Bait IDs follow a standardized format: ID-Accession-Species-details, where ID was a numeric identifier, Accession was the GenBank accession number, and Species was the taxonomic name. We used a combination of *AWK* and *sort* to extract, count reads per taxon, and rank by abundance. This produced per-sample count files showing how many reads mapped to each taxon.

One methodological choice we made was to not apply mapping quality (MAPQ) filtering. MAPQ scores indicate alignment confidence, with low MAPQ suggesting that a read maps equally well to multiple locations. We chose not to apply this filter because enrichment targets include many closely related taxa (e.g., Bison and Bos share ~98% mitochondrial similarity), and filtering these aDNA fragments would remove reads that legitimately align to shared conserved regions across taxa. This decision may explain why we detected many low-abundance taxa in Analysis 3 such that: some of these detections may represent reads mapping to multiple conserved regions, and therefore alignment “artifacts”. However, because our goal was to assess maximum enrichment sensitivity rather than to generate definitive taxonomic assignments, we opted for the more inclusive approach.

After alignment and taxonomic assignment, we calculated how many reads mapped to each taxon, per library. For each taxon in each sample, we recorded the sample ID, sample type (shotgun or enriched), taxon name, and read count. We then calculated mean read counts per taxon for shotgun and enriched samples separately. For shotgun samples, we computed the mean across all shotgun libraries: Mean Shotgun Reads = (1/*n_shotgun*) x Σ(*reads_i*), where n_shotgun is the number of shotgun samples and *reads_i* is the read count for that taxon in sample *i*. We used the same formula for enriched samples. Missing values were denoted with zero to enable fold enrichment calculations. This approach captured the full range of enrichment outcomes, including taxa that were undetectable in shotgun sequencing but recovered after enrichment.

For each taxon, we calculated fold enrichment as: Fold Enrichment = Mean Enriched Reads / Mean Shotgun Reads, but only when Mean Shotgun Reads > 0. When a taxon had zero shotgun reads, fold enrichment was mathematically undefined (i.e., division by zero), so we assigned a value of 0 to indicate that the taxon was only detected after enrichment. This conservative approach distinguishes between taxa that truly enrich (ratio > 1) and taxa that appear *de novo* after enrichment (shotgun count = 0). We binned taxa into six enrichment categories based on fold enrichment values: <1X (depleted or not enriched), 1-2X (minimal enrichment), 2-5X (moderate enrichment), 5-10X (good enrichment), 10-100X (strong enrichment), and >100X (exceptional enrichment) (Fig. 5), and computed global statistics summarizing enrichment performance across all taxa. These included total number of unique taxa detected, mean shotgun coverage across all taxa, mean enriched coverage across all taxa, number and percentage of taxa achieving ≥2X enrichment, and number and percentage of taxa achieving ≥5X enrichment. We also tabulated the number of taxa in each enrichment category to show the distribution of enrichment outcomes (see Dataset-S2.xlsx).

*LCA enrichment by sample:* we also calculated the fold-enrichment of *MEGAN7 LCA* assigned reads relative to their counterpart shotgun libraries. This analysis followed the same methodology as the primary plant/animal metagenomic assignment (Fig. 2). Enriched and shotgun libraries were proportionally counted for their *LCA* assigned reads (those with *BLASTn* alignments that passed all stages of filtering and could be assigned to a taxonomic node in *MEGAN7*) relative to the total sequencing depth of each library. Fold enrichment was calculated as the proportion enrich assigned / proportion shotgun assigned by sample.

*3) Divergence: SedaDNA read-to-bait and sedaDNA/bait-to-reference*. We measured sequence divergence between enriched sedaDNA sequencing reads and the RNA baits from which they were captured. This analysis directly assessed enrichment specificity: captured sedaDNA reads should closely match their corresponding baits, showing much lower divergence than the bait-to-reference divergence as measured above (i.e., Analysis 1).

Adapter removal, filtering, and taxonomic assignment was the same as analysis 2 (fold enrichment). For each aligned read, we extracted the edit distance from the *NM* tag added by BWA. The *NM* tag contains the number of mismatches between the aligned read to bait (as detailed above). We also recorded the read length (query length in *BAM* format), and calculated divergence percentage for each read as: Divergence (%) = (Edit Distance / Read Length) x 100.

We performed this analysis for two different reference sets: the *FASTA* formatted bait-set and the "FullRefs" (refseqs) analysis used whole mitochondrial genomes and chloroplast genes as the alignment references. The aggregated results across all samples and taxa were compiled using Python’s *pandas* library. For each taxon, the number of reads, mean edit distance, mean divergence percentage, median divergence, and mean read length was calculated.

To directly compare read-to-bait divergence with the bait-to-reference divergence measured in Analysis 1, we compiled statistics for the subset of baits that had corresponding read data (Dataset-S3.xlsx). The overall analysis used all 1,402,036 enriched reads showing mean divergence of 3.940%, while for visualization purposes, we randomly subsampled 100,000 reads (preserving the divergence distribution with mean 3.951%) (Fig. S10). Comparison with 169,972 bait sequences and enriched aDNA reads from the same seven animal taxa showed a mean edit distance of 1.60 mismatches and 3.95% divergence (100k subsample), while baits showed a mean edit distance of 15.928 mismatches and 19.910% divergence. This represents approximately 5-fold lower divergence for aDNA reads compared to baits.

Our analysis confirms that enrichment is highly selective. Despite baits showing ~20% divergence from reference genomes, the actual captured reads show only ~4% divergence. This selectivity occurs because enrichment via hybridization captures DNA fragments that are most complementary to the bait sequences. Fragments with too many mismatches do not hybridize efficiently and therefore remain in the off-target fraction of the library prior to in-solution amplification of on-target DNAs.

One important technical consideration is how read length affects divergence measurements. Because divergence is calculated as (Edit Distance / Read Length) × 100, shorter reads will show higher divergence percentages for the same absolute number of mismatches (Fig. S10). For example, 5 mismatches in a 24 bp read yields 20.8% divergence, while 5 mismatches in a 50 bp read yields only 10.0% divergence. This length dependency explains why our divergence distribution extends to 12-15% even though the edit distance distribution peaks at only 0-2 mismatches. The tail of the divergence distribution represents short reads with moderate numbers of mismatches, not reads with many mismatches. This variable length produces hundreds of possible divergence values, creating a smooth, continuous distribution when visualized as a histogram, rather than the gapped pattern observed for baits. When comparing read and bait divergence histograms, the smooth blue distribution (reads) versus gapped red distribution (baits) directly reflects this difference in sequence length variability (Fig. S10).

Modern human DNA methods

Enriched and shotgun sequenced libraries were evaluated for their modern human DNA contamination by analyzing human mtDNA using the nf-core EAGER (Efficient Ancient Genome Reconstruction) pipeline (version 2.5.3; <https://nf-co.re/eager> (Peltzer et al., 2016). Prior to bioinformatic analysis, two sets of paired R1/R2 (enrich1) *FASTQ* files were merged into a single paired-end library via a custom bash script (detailed in Supplementary Materials). Quality assessment of raw *FASTQ* reads was conducted using *FastQC* (<https://www.bioinformatics.babraham.ac.uk/projects/fastqc/>), with results aggregated into unified MultiQC reports (Ewels et al., 2016). The complete set of customized parameters employed during EAGER processing is provided below. The analytical workflow proceeded as follows: paired-end *FASTQ* data underwent adapter trimming and read merging (with singleton retention) via AdapterRemoval2 (<https://github.com/MikkelSchubert/adapterremoval>). Subsequently, processed *FastQ* files were aligned against the revised Cambridge Reference Sequence mitochondrial genome (rCRS; NC_012920) utilizing *CircularMapper* (<https://github.com/apeltzer/CircularMapper>). Post-alignment processing involved *SAM* to *BAM* file conversion with filtering criteria of minimum 24 bp read length and mapping quality ≥30, implemented through Samtools (<https://github.com/samtools/samtools>) (Schubert et al., 2016). Duplicate read identification and elimination was performed using *Picard's* *MarkDuplicates* tool (<https://gatk.broadinstitute.org/hc/en-us/articles/360037052812-MarkDuplicates-Picard->). Single nucleotide polymorphisms (SNP) were called using the *UniversalGenotyper* function from *GATK* v3.5 (Genome Analysis Toolkit). Consensus sequences were generated for filtered and mapped libraries with a minimum 5x depth of coverage, using *VCF2Genome* (<https://github.com/apeltzer/VCF2Genome>).

*Modern human DNA results*

Sequenced reads were analyzed for human mtDNA via the nf-core EAGER pipeline (Peltzer et al., 2016) to characterize our modern contamination. The negative controls produced spurious reads mapping to the rCRS (revised Cambridge Reference Sequence), likely due to minimal contamination from the lab environment or reagents. Similarly, two enriched pools revealed only sporadic mapped, filtered, and deduplicated reads (e.g., <0.3x depth of coverage across the rCRS) mapping to the human mitochondrial genome.

Additional results benchmarking PalaeoChip Arctic v1.0 with temperate woodland biota

*Comparative Fold-Enrichment Results*

Bait-set analysis detected 756 unique taxa across all samples. Mean shotgun coverage was 1.26 ± 2.24 reads per taxon, while mean enriched coverage was 60.62 ± 128.32 reads per taxon, an average 48x increase. Because the distribution of fold-enrichment values was highly skewed, we also report category counts: 378 taxa (50.0%) achieved ≥2x enrichment, 333 (44.0%) achieved ≥5x, and 358 (47.4%) had zero shotgun reads but were detected after enrichment.

By category (Bait-set): zero-shotgun 358 (47.4%); depleted <1x, 3 (0.4%); 1–2x, 17 (2.2%); 2–5, 45 (6.0%); 5–10x, 49 (6.5%); 10–100x, 238 (31.5%); >100x, 46 (6.1%). Among taxa with ≥2x enrichment, ~63% (238/378) fall in the 10–100x range. FullRefs detected 3,543 taxa with mean shotgun 0.677 ± 6.74 and enriched 42.22 ± 75.93 reads/taxon (overall fold 62.33×). ≥2x enrichment occurred in 744 (21.0%) taxa and ≥5x in 687 (19.4%). Zero-shotgun were measured for 2,751 (77.6%) taxa, underscoring the panel’s sensitivity to otherwise undetectable targets, and indicating strong, widespread enrichment across a broad taxonomic rank.

The lower enrichment rates but higher taxonomic diversity in the “FullRefs” analysis reflects a fundamental trade-off when bioinformatically analyzing capture data: sensitivity increases (more taxa detected), while specificity can be reduced by short, low-quality alignments. In contrast, bait-set analysis using 80 bp fragments is more stringent, detecting fewer taxa overall but with higher confidence that detected taxa are genuinely enriched.

*Shotgun Coverage Distribution and Rare Taxa*

The distribution of shotgun coverage values was highly skewed toward low counts. For bait-set analysis, 358 taxa (47.4%) had zero shotgun reads, 81 taxa (10.7%) had exactly one read, 112 taxa (14.8%) had exactly two reads, 105 taxa (13.9%) had 3-5 reads, and only 100 taxa (13.2%) had more than five reads (Dataset-S2.xlsx). This distribution demonstrates that the majority of taxa in sedaDNA libraries occur at extremely low abundance, and often below the detection threshold of shotgun sequencing, or detected at threshold levels of 1-2 reads.

Our enrichment analysis demonstrates that these rare taxa are preferentially amplified by PalaeoChip Arctic v1.0. Many taxa with only 1-2 shotgun reads achieved 10-100x enrichment, resulting in 10-200 enriched reads. Shotgun metagenomic sequencing of sedaDNA is dominated by abundant taxa (typically bacteria and fungi in sediment samples), with eukaryotic macroorganisms comprising <1% of total reads. By selectively amplifying target taxa with sequence-specific baits, PalaeoChip Arctic v1.0 enables detection and analysis of rare eukaryotic DNA that would be effectively undetectable in shotgun data.

*Sequence Conservation*

One potential confounding factor in divergence analysis is the presence of conserved sequences that map to multiple taxa. For example, highly conserved mitochondrial genes might produce baits that align well to both *Bison* and *Mammuthus* references. If these shared sequences have unusually high divergence (perhaps due to the bait originating from a third species), they could inflate overall divergence estimates when analyzed as single-taxon alignments.

We tested this hypothesis by identifying baits that mapped to multiple taxa and comparing their divergence to taxon-specific baits. Using *Bison* and *Mammuthus* as an example, we extracted all bait IDs that appeared in both *BAM* files, representing shared sequences. We then calculated mean divergence for shared versus unique (taxon-specific) baits in each genome. For *Bison*, shared baits showed 16.57% divergence while unique baits showed 18.39% divergence, a difference of -1.82 percentage points. For *Mammuthus*, shared baits showed 21.31% divergence while unique baits showed 21.59% divergence, a difference of -0.28 percentage points.

We extended this analysis to all 21 possible pairwise comparisons among the seven animal taxa from the bait-to-reference divergence and fold enrichment analysis. Of these 21 comparisons, 17 (81%) showed that shared sequences had lower divergence than unique sequences (Fig. S11). The remaining four comparisons showed slightly higher divergence for shared sequences, but the differences were minimal (<1 percentage point). This pattern demonstrates that conserved sequences are generally well-preserved across taxa and do not artificially inflate divergence estimates. In fact, they slightly reduce mean divergence because conserved regions are, by definition, more similar across species than rapidly evolving, hypervariable regions.

*Ancient DNA Read-to-Bait Divergence Results*

Bait-set analysis of 1,402,036 enriched reads from 757 unique taxa revealed excellent enrichment specificity. The mean edit distance was 1.593 ± 1.22 mismatches, and mean divergence was 3.940% ± 3.01%. The median divergence of 3.922% was nearly identical to the mean, indicating a symmetric distribution with most reads showing low divergence. For the bait-set, 99.38% of reads (1,393,388/1,402,036) had ≤10% divergence; for FullRefs, 99.57% (4,200,230/4,218,307). The mean read length was 40.41 ± 7.30 bp, consistent with the overall quality-filtered read length distribution, demonstrating that reads of all lengths were successfully enriched (see Dataset-S3.xlsx).

We observed lower divergence for the “FullRefs” analysis of 4,218,307 reads, with a mean edit distance of only 1.05 ± 1.17 mismatches and mean divergence of 2.713% ± 3.01%. The median divergence was 2.13%, substantially lower than for the bait-set analysis. The proportion of reads with divergence ≤10% was 99.57% (4,200,230/4,218,307 reads). The mean read length for FullRefs analysis was 38.12 ± 6.95 bp, slightly shorter than for bait-set analysis. The much larger number of reads in FullRefs analysis (4,218,307 vs 1,402,036) reflects the fact that full-length mtDNA and chloroplast references capture more alignments across diverse taxa, including reads that may align to reference regions outside the 80 bp bait targets.

The lower divergence observed in FullRefs analysis (2.71% vs 3.94%) likely reflects two factors. First, full-length references provide more context for accurate alignment, potentially reducing spurious mismatches. Second, the “FullRefs” dataset included more diverse taxonomic coverage and therefore captured a higher proportion of well-preserved, low-divergence reads across a broader range of species.

*Ancient DNA Read-to-Bait Divergence Discussion*

The read-to-bait divergence results demonstrate that PalaeoChip Arctic v1.0 effectively captures divergent molecules up to ~10%. Mean divergence of 3.94% for reads versus 19.91% for baits confirms that hybridization-based enrichment successfully captures sequences that are more similar to baits than the baits themselves are to reference genomes. This finding validates the enrichment approach: even when baits are designed from species different from the target organisms (resulting in 18-25% bait-to-reference divergence), the physical process of hybridization selects for the most complementary sequences available in the sample.

Both analyses show very high proportions of low-divergence sedaDNA reads (bait-set 99.38%; FullRefs 99.57%), and likely reflects the more stringent alignment requirements when mapping to full-length genes rather than 80 bp bait fragments, combined with the inclusion of more divergent taxa that were not well-represented in the bait design. Nevertheless, the “FullRefs” analysis successfully detected 3,530 unique taxa across all samples, demonstrating the broad taxonomic coverage achieved by PalaeoChip Arctic v1.0.

*Bait panel summary*

PalaeoChip Arctic v1.0 successfully enriched eukaryotic biota from temperate woodland sedimentary cores from Crawford Lake. Despite broad bait-to-reference divergence (~18–25% for animal mtDNA; ~19–24% for plant chloroplast targets *rbcL*, *matK*, *trnL*-UAA), captured molecules are much closer to their targets (≈4% read-to-bait; ≈2.7% read-to-full-reference), showing that hybridization retains the most complementary fragments during enrichment. Additionally, enrichment amplifies ecological signal: relative to shotgun, mean per-taxon reads increased by ~48x against the bait *FASTA* and ~62x against full genomes/genes, with nearly half of bait-set taxa and over three-quarters of full-reference taxa undetectable by shotgun alone. These results show the panel reliably recovers rare eukaryotic signals in sedimentary DNA from different ecological/climactic zones despite the panel’s focus on Arctic-based plants and animals. Overall, PalaeoChip Arctic v1.0 robustly enriches low-abundance fragments and expands taxonomic recovery above shotgun baselines.

*BLAST length/score floor calculations*

**Reference**. We measured the length/identity/score relationship directly with BLAST+ (v2.17.0). We generated one fixed 2,000 bp random nucleotide sequence as a synthetic reference. For every read length *L* from 18 to 120 bp and every target percent identity in {100, 99, 98, 97, 96, 95}%, we built one synthetic query as a substring of the reference with round((100 − identity) / 100 × L) positions substituted to a different base. This gives a query of exactly known length and (very close to) the intended percent identity to the reference at every point on a length × identity grid: 103 lengths × 6 identities × 2 mismatch-placement strategies × 2 BLAST tasks = 2,472 synthetic alignments in total. The code for this analysis is included as a supplementary code file.

**Mismatch placement**. Where mismatches fall along a read, not just how many there are, determines whether a seed-based aligner like *MegaBLAST* can find the alignment at all. We therefore tested two deterministic (non-random, so the results are smooth and reproducible rather than noisy from one random placement draw to the next) placement strategies: - `even`: mismatches spread evenly through the read interior (kept off the outermost 2 bp on each end). This is a conservative/adversarial case for seed-and-extend aligners, since spreading mismatches through the middle of a short read is the most effective way to prevent any long exact-match window from existing. - `ends`: mismatches concentrated at the two termini, alternating 5′/3′. This mimics the terminal-biased cytosine-deamination misincorporation pattern characteristic of genuinely ancient, damaged DNA (as opposed to mismatches from general sequencing error or reference-database divergence, which are less positionally biased). This leaves the read's interior intact, so a clean seed window is much more likely to survive.

**Alignment**. Each synthetic query was searched against the reference with blastn -task blastn or -task megablast (matching the two classification pipelines' defaults) using each task's default scoring/gap-cost/word-size parameters, and a deliberately permissive -evalue 1000. This ensures BLAST reports a hit whenever its seed-and-extend algorithm finds any alignment at all, rather than silently discarding a genuine but low-scoring alignment via the e-value cutoff. A "no hit" result therefore reflects a structural seeding failure, not an e-value reporting threshold artifact.

**Recording**. For every (task, placement, length, target identity) combination we recorded whether BLAST reported a hit, that hit's measured percent identity/raw score/bit score, and whether the bit score met *MEGAN's* MinScore = 50 threshold.

**Interpretation**. Under the biologically realistic terminal ("ends") scenario, the pattern expected for genuinely ancient, deaminated DNA, the floor is low and essentially flat across identity levels: 27–28 bp for *BLASTn*, 28–29 bp for *MegaBLAST*. This closely matches what we observe empirically in the real dataset (31 bp / 30 bp minimum assigned lengths). Under the adversarial interior ("even") scenario, the floor rises sharply once identity drops below ~98%, because scattering mismatches through the middle of a short read is highly effective at destroying every possible *MegaBLAST* seed window (*BLASTn’s* smaller 11 bp word size makes it far less sensitive to this effect). We report both so the dependency on mismatch position is transparent, but consider the "ends" scenario the operative one for this study's authentically ancient molecules. Also note that for *BLASTn*, the "any hit" and "passes MinScore" columns diverge (e.g. even/97%: 22 bp vs 30 bp), the bit-score threshold is doing independent work there. For *MegaBLAST*, the two columns are identical in every row tested, once *MegaBLAST's* seed requirement is satisfied at all, the resulting alignment already clears MinScore = 50, meaning the *seed word size*, not the bit score, is the binding constraint for the off-target pipeline.

*Nuclear DNA*

We briefly evaluated the nuclear vs organellar component of our dataset by mapping the reads from our nine shotgun libraries (mean 6.2 million reads) and 26 enriched libraries (mean 2.3 million reads) to the *Branta* *canadensis* mitogenome (NC_007011) and to a chromosome-level *Branta* *canadensis* nuclear assembly (GCF_965231335.1) using the same methods as described in the main text and counted unique reads at mapping quality ≥30. Mitochondrial recovery behaves as expected for a genuine capture target: unique *Branta* mtDNA molecules rose from 226 in the shotgun data (4.0 unique reads per million reads sequenced) to 27,738 with capture. Our capture data was highly duplicated due to the two rounds of enrichment (Fig S45). We recommend one round of enrichment for complex ecological enrichments such as this in the future.

Many reads mapped to the nuclear assembly (~1.04 million unique shotgun reads, ~18,600 per million), but these seem to be non-specific background rather than authentic *Branta* nuclear DNA, based on 4 lines of evidence: (i) they exceed the mitochondrial signal ~4,600-fold, which is unexpected for a rare taxon with a high-copy mitogenome; (ii) they are short (mean 26 bp) and low-identity (only ~2% map at ≤1 mismatch, versus 69–87% for the mtDNA reads); (iii) they anti-correlate with the genuine mitochondrial signal, meaning the library with the most nuclear hits (CLO-5, 279,150 unique) has the fewest mtDNA reads (7), while the most mtDNA-rich library (CLO-40) has among the fewest nuclear hits; (iv) in a competitive test against a distant-bird decoy (chicken, *Gallus gallus*), ~90% as many unique reads map to chicken as to *Branta*, and the residual goose-preferential fraction (reads matching *Branta* with fewer mismatches than chicken) is still ~3,000× the mitochondrial signal and itself anti-correlates with it (r = −0.36). Meaning, it reflects the waterfowl/avian background of a lake catchment, phylogenetically nearer goose than chicken, not authentic *B. canadensis*. In this example, the 1.3-Gb nuclear assembly simply acts as a magnet for the conserved fraction of any vertebrate-bearing metagenome, and that background cannot be easily separated from the small genuine nuclear contribution of a single species. For this reason, we did not explore the nuclear genetic potential of this dataset in this manuscript as this would constitute a much larger secondary analysis, potentially involving nuclear bait enrichment.

**Fig. S1 Shifting sedaDNA abundance through time.** Top 10 most abundant plant/animal taxonomic assignments. Settlement phases assumed based on two/three main occupation periods and their close spatial-temporal proximity, although their direct correlation is unconfirmed.

**Fig. S2 Stacked bar chart of “golden” algae.** Normalized read counts to the smallest library.

**Fig. S3 MtDNA phylogenetics of *B. canadensis* sedaDNA.** (**A**) Maximum likelihood tree of publicly available *B. canadensis* COX1 and (**B**) control region sequences.

**Fig. S4 Chloroplast assemblies, phylogenetics, and damage patterns of *Zea mays* (maize/corn) sedaDNA.** (**A**) Maximum likelihood tree of publicly available rbcL genes. (**B**) *mapDamage* fragment length distributions and (**C**) deamination rates of sedaDNA assemblies.

**Fig. S5 Chloroplast assemblies, phylogenetics, and damage patterns of *Helianthus annuus* (common sunflower) sedaDNA.** (**A**) Maximum likelihood tree of publicly available *rbc*L *Helianthus* sequences. (**B**) *mapDamage* fragment length distributions and (**C**) deamination rates of sedaDNA assemblies. The bimodal fragment lengths in the FLD are primarily driven by off-target mapping of related taxa in homologous regions. The rbcL assemblies were competitively mapped to remove this off-target signal and polymorphisms in regions of read stacking were N-masked.

**Fig. S6 Chloroplast assemblies, phylogenetics, and damage patterns of Pinaceae sedaDNA.** (**A**) Maximum likelihood tree of publicly available rbcL genes. (**B**) *mapDamage* fragment length distributions and (**C**) deamination rates of sedaDNA assemblies.

**Fig. S7 metaDMG plots of damage versus significance for top abundance taxonomic families.** (**A**) All libraries merged and (**B**) Libraries merged by ecological zone.

**Fig. S8 Bait-to-reference edit distances.** (**A**) Mean edit distance by taxon, (**B**) distribution of plants and animals, and (**C**) distribution by taxon.

**Fig. S9 SedaDNA divergences.** (**A**) Read-to-bait and (**B**) read-to-reference with cumulative distributions.

**Fig. S10 Edit distance, divergence, and read length with reads and baits to references.** (**A**) Edit distance and divergence of sedaDNA reads and baits relative to the references. (**B**) Read length versus divergence.

**Fig. S11 Reference similarity for evaluating bait cross-capture.** (**A**) Conservation and divergence amongst animal taxa and (**B**) similarity and divergence with the 11 tested plant/animal references.

**Fig. S12 Animal bait mapping to select reference genomes.**

**Fig. S13 Plant bait mapping to select reference genomes.**

**Fig. S14 Age-depth model for core CL-19.**

**Fig. S15 Age-depth model re-analyzing the data from Ekdahl et al. (2004) with IntCal20.**

**Fig. S16 MetaMerge heatmap of plant and algal sedaDNA from the on-target analysis with conservative MEGAN LCA hits merged with Holi/metaDMG damage support.** Page 1 of 5.

**Fig. S16 (continued, page 2 of 5)**

**Fig. S16 (continued, page 3 of 5)**

**Fig. S16 (continued, page 4 of 5)**

**Fig. S16 (continued, page 5 of 5)**

**Fig. S17 As Fig. S16, but cell colour indicates the metaDMG maximum-likelihood C→T damage estimate (δs) per taxon–library combination.** Higher values (darker) reflect greater deamination-associated aDNA damage. Page 1 of 5.

**Fig. S17 (continued, page 2 of 5)**

**Fig. S17 (continued, page 3 of 5)**

**Fig. S17 (continued, page 4 of 5)**

**Fig. S17 (continued, page 5 of 5)**

**Fig. S18 Stacked bar chart of plant, algal, and animal sedaDNA read-count proportions across Crawford Lake depth intervals from the on-target analysis.**

**Fig. S19 MetaMerge heatmap of animal sedaDNA detected in Crawford Lake sediment core, on-target analysis.**

**Fig. S20 As Fig. S19, but cell colour indicates metaDMG C→T damage estimate (δs).**

**Fig. S21 Stacked bar chart of animal sedaDNA read-count proportions across Crawford Lake depth intervals (on-target analysis).**

**Fig. S22 MetaMerge heatmap of plant and algal sedaDNA detected using off-target MegaBLAST analysis (40 individual enriched and shotgun libraries merged by biosample.** Read counts are normalized; cells displaying 0 represent 0–1 normalized reads.

**Fig. S22 (continued, page 2 of 53)**

**Fig. S22 (continued, page 3 of 53)**

**Fig. S22 (continued, page 4 of 53)**

**Fig. S22 (continued, page 5 of 53)**

**Fig. S22 (continued, page 6 of 53)**

**Fig. S22 (continued, page 7 of 53)**

**Fig. S22 (continued, page 8 of 53)**

**Fig. S22 (continued, page 9 of 53)**

**Fig. S22 (continued, page 10 of 53)**

**Fig. S22 (continued, page 11 of 53)**

**Fig. S22 (continued, page 12 of 53)**

**Fig. S22 (continued, page 13 of 53)**

**Fig. S22 (continued, page 14 of 53)**

**Fig. S22 (continued, page 15 of 53)**

**Fig. S22 (continued, page 16 of 53)**

**Fig. S22 (continued, page 17 of 53)**

**Fig. S22 (continued, page 18 of 53)**

**Fig. S22 (continued, page 19 of 53)**

**Fig. S22 (continued, page 20 of 53)**

**Fig. S22 (continued, page 21 of 53)**

**Fig. S22 (continued, page 22 of 53)**

**Fig. S22 (continued, page 23 of 53)**

**Fig. S22 (continued, page 24 of 53)**

**Fig. S22 (continued, page 25 of 53)**

**Fig. S22 (continued, page 26 of 53)**

**Fig. S22 (continued, page 27 of 53)**

**Fig. S22 (continued, page 28 of 53)**

**Fig. S22 (continued, page 29 of 53)**

**Fig. S22 (continued, page 30 of 53)**

**Fig. S22 (continued, page 31 of 53)**

**Fig. S22 (continued, page 32 of 53)**

**Fig. S22 (continued, page 33 of 53)**

**Fig. S22 (continued, page 34 of 53)**

**Fig. S22 (continued, page 35 of 53)**

**Fig. S22 (continued, page 36 of 53)**

**Fig. S22 (continued, page 37 of 53)**

**Fig. S22 (continued, page 38 of 53)**

**Fig. S22 (continued, page 39 of 53)**

**Fig. S22 (continued, page 40 of 53)**

**Fig. S22 (continued, page 41 of 53)**

**Fig. S22 (continued, page 42 of 53)**

**Fig. S22 (continued, page 43 of 53)**

**Fig. S22 (continued, page 44 of 53)**

**Fig. S22 (continued, page 45 of 53)**

**Fig. S22 (continued, page 46 of 53)**

**Fig. S22 (continued, page 47 of 53)**

**Fig. S22 (continued, page 48 of 53)**

**Fig. S22 (continued, page 49 of 53)**

**Fig. S22 (continued, page 50 of 53)**

**Fig. S22 (continued, page 51 of 53)**

**Fig. S22 (continued, page 52 of 53)**

**Fig. S22 (continued, page 53 of 53)**

**Fig. S23 As Fig. S22, but cell colour indicates metaDMG C→T damage estimate (δs).**

**Fig. S23 (continued, page 2 of 53)**

**Fig. S23 (continued, page 3 of 53)**

**Fig. S23 (continued, page 4 of 53)**

**Fig. S23 (continued, page 5 of 53)**

**Fig. S23 (continued, page 6 of 53)**

**Fig. S23 (continued, page 7 of 53)**

**Fig. S23 (continued, page 8 of 53)**

**Fig. S23 (continued, page 9 of 53)**

**Fig. S23 (continued, page 10 of 53)**

**Fig. S23 (continued, page 11 of 53)**

**Fig. S23 (continued, page 12 of 53)**

**Fig. S23 (continued, page 13 of 53)**

**Fig. S23 (continued, page 14 of 53)**

**Fig. S23 (continued, page 15 of 53)**

**Fig. S23 (continued, page 16 of 53)**

**Fig. S23 (continued, page 17 of 53)**

**Fig. S23 (continued, page 18 of 53)**

**Fig. S23 (continued, page 19 of 53)**

**Fig. S23 (continued, page 20 of 53)**

**Fig. S23 (continued, page 21 of 53)**

**Fig. S23 (continued, page 22 of 53)**

**Fig. S23 (continued, page 23 of 53)**

**Fig. S23 (continued, page 24 of 53)**

**Fig. S23 (continued, page 25 of 53)**

**Fig. S23 (continued, page 26 of 53)**

**Fig. S23 (continued, page 27 of 53)**

**Fig. S23 (continued, page 28 of 53)**

**Fig. S23 (continued, page 29 of 53)**

**Fig. S23 (continued, page 30 of 53)**

**Fig. S23 (continued, page 31 of 53)**

**Fig. S23 (continued, page 32 of 53)**

**Fig. S23 (continued, page 33 of 53)**

**Fig. S23 (continued, page 34 of 53)**

**Fig. S23 (continued, page 35 of 53)**

**Fig. S23 (continued, page 36 of 53)**

**Fig. S23 (continued, page 37 of 53)**

**Fig. S23 (continued, page 38 of 53)**

**Fig. S23 (continued, page 39 of 53)**

**Fig. S23 (continued, page 40 of 53)**

**Fig. S23 (continued, page 41 of 53)**

**Fig. S23 (continued, page 42 of 53)**

**Fig. S23 (continued, page 43 of 53)**

**Fig. S23 (continued, page 44 of 53)**

**Fig. S23 (continued, page 45 of 53)**

**Fig. S23 (continued, page 46 of 53)**

**Fig. S23 (continued, page 47 of 53)**

**Fig. S23 (continued, page 48 of 53)**

**Fig. S23 (continued, page 49 of 53)**

**Fig. S23 (continued, page 50 of 53)**

**Fig. S23 (continued, page 51 of 53)**

**Fig. S23 (continued, page 52 of 53)**

**Fig. S23 (continued, page 53 of 53)**

**Fig. S24 Stacked bar chart of plant, algal, and animal sedaDNA read-count proportions across Crawford Lake libraries (off-target analysis).**

**Fig. S25 MetaMerge heatmap of animal sedaDNA detected using off-target MegaBLAST analysis.** Read counts are normalized; cells displaying 0 represent 0–1 normalized reads.

**Fig. S25 (continued, page 2 of 12)**

**Fig. S25 (continued, page 3 of 12)**

**Fig. S25 (continued, page 4 of 12)**

**Fig. S25 (continued, page 5 of 12)**

**Fig. S25 (continued, page 6 of 12)**

**Fig. S25 (continued, page 7 of 12)**

**Fig. S25 (continued, page 8 of 12)**

**Fig. S25 (continued, page 9 of 12)**

**Fig. S25 (continued, page 10 of 12)**

**Fig. S25 (continued, page 11 of 12)**

**Fig. S25 (continued, page 12 of 12)**

**Fig. S26 As Fig. S25, but cell colour indicates metaDMG C→T damage estimate (δs).**

**Fig. S26 (continued, page 2 of 12)**

**Fig. S26 (continued, page 3 of 12)**

**Fig. S26 (continued, page 4 of 12)**

**Fig. S26 (continued, page 5 of 12)**

**Fig. S26 (continued, page 6 of 12)**

**Fig. S26 (continued, page 7 of 12)**

**Fig. S26 (continued, page 8 of 12)**

**Fig. S26 (continued, page 9 of 12)**

**Fig. S26 (continued, page 10 of 12)**

**Fig. S26 (continued, page 11 of 12)**

**Fig. S26 (continued, page 12 of 12)**

**Fig. S27 Stacked bar chart of animal sedaDNA read-count proportions across Crawford Lake libraries (off-target analysis).**

**Fig. S28 MetaMerge heatmap of fungal sedaDNA detected in Crawford Lake sediment core (off-target MegaBLAST analysis).** Read counts are normalized; cells displaying 0 represent 0–1 normalized reads.

**Fig. S28 (continued, page 2 of 2)**

**Fig. S29 As Fig. S28, but cell colour indicates metaDMG C→T damage estimate (δs).**

**Fig. S29 (continued, page 2 of 2)**

**Fig. S30 Stacked bar chart of fungal sedaDNA read-count proportions across Crawford Lake libraries (off-target analysis).**

**Fig. S31 MetaMerge heatmap of bacterial and archaeal sedaDNA detected in Crawford Lake sediment core (off-target MegaBLAST analysis).** Read counts are normalized; cells displaying 0 represent 0–1 normalized reads.

**Fig. S31 (continued, page 2 of 37)**

**Fig. S31 (continued, page 3 of 37)**

**Fig. S31 (continued, page 4 of 37)**

**Fig. S31 (continued, page 5 of 37)**

**Fig. S31 (continued, page 6 of 37)**

**Fig. S31 (continued, page 7 of 37)**

**Fig. S31 (continued, page 8 of 37)**

**Fig. S31 (continued, page 9 of 37)**

**Fig. S31 (continued, page 10 of 37)**

**Fig. S31 (continued, page 11 of 37)**

**Fig. S31 (continued, page 12 of 37)**

**Fig. S31 (continued, page 13 of 37)**

**Fig. S31 (continued, page 14 of 37)**

**Fig. S31 (continued, page 15 of 37)**

**Fig. S31 (continued, page 16 of 37)**

**Fig. S31 (continued, page 17 of 37)**

**Fig. S31 (continued, page 18 of 37)**

**Fig. S31 (continued, page 19 of 37)**

**Fig. S31 (continued, page 20 of 37)**

**Fig. S31 (continued, page 21 of 37)**

**Fig. S31 (continued, page 22 of 37)**

**Fig. S31 (continued, page 23 of 37)**

**Fig. S31 (continued, page 24 of 37)**

**Fig. S31 (continued, page 25 of 37)**

**Fig. S31 (continued, page 26 of 37)**

**Fig. S31 (continued, page 27 of 37)**

**Fig. S31 (continued, page 28 of 37)**

**Fig. S31 (continued, page 29 of 37)**

**Fig. S31 (continued, page 30 of 37)**

**Fig. S31 (continued, page 31 of 37)**

**Fig. S31 (continued, page 32 of 37)**

**Fig. S31 (continued, page 33 of 37)**

**Fig. S31 (continued, page 34 of 37)**

**Fig. S31 (continued, page 35 of 37)**

**Fig. S31 (continued, page 36 of 37)**

**Fig. S31 (continued, page 37 of 37)**

**Fig. S32 As Fig. S31, but cell colour indicates metaDMG C→T damage estimate (δs).**

**Fig. S32 (continued, page 2 of 37)**

**Fig. S32 (continued, page 3 of 37)**

**Fig. S32 (continued, page 4 of 37)**

**Fig. S32 (continued, page 5 of 37)**

**Fig. S32 (continued, page 6 of 37)**

**Fig. S32 (continued, page 7 of 37)**

**Fig. S32 (continued, page 8 of 37)**

**Fig. S32 (continued, page 9 of 37)**

**Fig. S32 (continued, page 10 of 37)**

**Fig. S32 (continued, page 11 of 37)**

**Fig. S32 (continued, page 12 of 37)**

**Fig. S32 (continued, page 13 of 37)**

**Fig. S32 (continued, page 14 of 37)**

**Fig. S32 (continued, page 15 of 37)**

**Fig. S32 (continued, page 16 of 37)**

**Fig. S32 (continued, page 17 of 37)**

**Fig. S32 (continued, page 18 of 37)**

**Fig. S32 (continued, page 19 of 37)**

**Fig. S32 (continued, page 20 of 37)**

**Fig. S32 (continued, page 21 of 37)**

**Fig. S32 (continued, page 22 of 37)**

**Fig. S32 (continued, page 23 of 37)**

**Fig. S32 (continued, page 24 of 37)**

**Fig. S32 (continued, page 25 of 37)**

**Fig. S32 (continued, page 26 of 37)**

**Fig. S32 (continued, page 27 of 37)**

**Fig. S32 (continued, page 28 of 37)**

**Fig. S32 (continued, page 29 of 37)**

**Fig. S32 (continued, page 30 of 37)**

**Fig. S32 (continued, page 31 of 37)**

**Fig. S32 (continued, page 32 of 37)**

**Fig. S32 (continued, page 33 of 37)**

**Fig. S32 (continued, page 34 of 37)**

**Fig. S32 (continued, page 35 of 37)**

**Fig. S32 (continued, page 36 of 37)**

**Fig. S32 (continued, page 37 of 37)**

**Fig. S33 Stacked bar chart of bacterial and archaeal sedaDNA read-count proportions across Crawford Lake libraries (off-target analysis).**

**Fig. S34 CONISS (constrained incremental sum of squares) cluster dendrogram for Crawford Lake plant, algal, and animal sedaDNA assemblages across 28 depth intervals.** Split on-target/off-target, and combined analyses. Only MetaMerge supported taxa included. Ward's minimum-variance criterion with stratigraphic constraint (only adjacent depth levels may merge). The broken-stick null model (Bennett 1996) supports two statistically significant zones (dashed line) that falls either between pre-farming and farming (on-target) or farming/post-farming and Euro-Canadaian (off-target).

**Fig. S35 Stratigraphic diagram of top 20 damage supported ecologically informative plant, algal, and animal sedaDNA read-count proportions across depth intervals (on-target analysis), with CONISS zones.**

**Fig. S36 Stratigraphic diagram of top 20 damage supported ecologically informative bacterial, archaeal, algal (off-target), and fungal sedaDNA read-count proportions across depth intervals (off-target analysis), with CONISS zones.**

**Fig. S37 Stratigraphic diagram of top 20 damage supported ecologically informative taxa from the on/off-target analyses combined (equal weighting between the two) with CONISS zones.**

**Fig. S38 metaDMG damage versus significance plots for prokaryotic taxa across Crawford Lake sediment libraries (sample-faceted).** Each panel represents one sequencing library. x-axis: maximum-likelihood C→T damage estimate (δs); y-axis: statistical significance.

**Fig. S39 As Fig. S38, but libraries grouped and colour-coded by stratigraphic era.**

**Fig. S40 Top prokaryotic genera detected across Crawford Lake sediment libraries by metaDMG-authenticated read proportions.**

**Fig. S41 As Fig. S40, but libraries grouped and colour-coded by stratigraphic era.**

**Fig. S42 Estimated internal alignment identity and C to T damage for ecologically interpreted microbial, algal, and fungal taxa.** Left panel: estimated internal alignment identity (= 1 − background C to T rate − non-CT/GA mismatch rate), derived from the metaDMG substitution frequency model. This metric quantifies per-position read-to-reference identity at internal read positions. Vertical dashed lines indicate the 95% identity threshold applied during MEGAN LCA classification (red) and the 97% threshold raised by reviewer 1 (orange). Right panel: 5′-terminal C to T damage amplitude (δs); stars indicate damage significance ≥ 2.0. Taxa are grouped by broad category (coloured left stripe) and coloured by aDNA confidence tier. Taxa with Tentative classification (orange) are included because they were ecologically discussed in the main text; their confidence reflects incomplete damage authentication rather than low alignment identity. All values are from the best-matched Holi/metaDMG library for each taxon.

**Fig. S43 Internal alignment identity for ecologically interpreted microbial, algal, and fungal taxa.** Left panel: internal alignment identity derived from the metaDMG substitution frequency model. This metric quantifies per-position read-to-reference identity at internal read positions. Vertical dashed lines indicate the 95% identity threshold applied during MEGAN LCA classification (red) and the 97% threshold raised by reviewer 1 (orange). Right panel: 5′-terminal C to T damage amplitude (δs); stars indicate damage significance ≥ 2.0. Taxa are grouped by broad category (coloured left stripe) and coloured by aDNA confidence tier. Taxa with Tentative classification (orange) are included because they were ecologically discussed in the main text; their confidence reflects incomplete damage authentication rather than low alignment identity. All values are from the best-matched Holi/metaDMG library for each taxon.

**Fig. S44 Fragment length distribution example of reads from MEGAN7 assigned taxonomic nodes.** Plots demonstrate that reads <30 bp are not assigned in this dataset despite the min 24 bp floor during initial processing. Only reads of a sufficient length accumulate the necessary metrics for LCA assignment. Part 1 of 2.

**Fig. S44 continuted.** Part 2 of 2.

**Fig. S45 PCR duplication between rounds of hybridization capture**.

**Fig. S46 Challenges of nuclear lake sedaDNA, initial explorations.**

**Supplementary Tables**

**Table S1 EAGER summary statistics for human mapped (rCRS) shotgun sequenced libraries.**

| **Sample Name** | **Nr. Reads Into Mapping** | **Nr. Mapped Reads (rCRS)** | **Nr. Mapped Reads Passed Post-Filter** | **5 Prime C>T 1st base** | **5 Prime C>T 2nd base** | **Mean Length Mapped Reads** | **Nr. Dedup. Mapped Reads** | **Mean cov** | **% GC Dedup. Mapped Reads** | **Endogenous DNA (%)** | **Endogenous DNA Post (%)** |
| --- | --- | --- | --- | --- | --- | --- | --- | --- | --- | --- | --- |
| **CLO-20-Shg_S33_L001** | 4563949 | 0 | 0 | n/a | n/a | n/a | n/a | n/a | n/a | 0 | 0 |
| **CLO-35-Shg_S27_L001** | 6088782 | 0 | 0 | n/a | n/a | n/a | n/a | n/a | n/a | 0 | 0 |
| **CLO-40-Shg_S34_L001** | 4892501 | 0 | 0 | n/a | n/a | n/a | n/a | n/a | n/a | 0 | 0 |
| **CLO-42-Shg_S28_L001** | 5743581 | 2 | 2 | 0.0% | 100.0% | 51.00bp | 2 | 0.0X | 46% | 0 | 0 |
| **CLO-45-Shg_S29_L001** | 4422111 | 0 | 0 | n/a | n/a | n/a | n/a | n/a | n/a | 0 | 0 |
| **CLO-5-Shg_S26_L001** | 2880547 | 0 | 0 | n/a | n/a | n/a | n/a | n/a | n/a | 0 | 0 |
| **CLO-57-Shg_S30_L001** | 4366324 | 0 | 0 | n/a | n/a | n/a | n/a | n/a | n/a | 0 | 0 |
| **CLO-60-Shg_S35_L001** | 7446169 | 1 | 1 | 0.0% | 0.0% | 35.00bp | 1 | 0.0X | 52% | 0 | 0 |
| **CLO-75-Shg_S31_L001** | 4449670 | 0 | 0 | n/a | n/a | n/a | n/a | n/a | n/a | 0 | 0 |
| **CLO-BK1-Shg_S32_L001** | 1538190 | 85 | 82 | 0.0% | 0.0% | 57.87bp | 42 | 0.1X | 46% | 0.01 | 0.01 |
| **LiB-BK-Shg_S25_L001** | 92 | 0 | 0 | n/a | n/a | n/a | n/a | n/a | n/a | 0 | 0 |
| ****n/a = no data available**** |  |  |  |  |  |  |  |  |  |  |  |

**Table S2 EAGER summary statistics for human mapped enriched libraries batch 1.**

| **Sample Name** | **Nr. Reads Into Mapping** | **Nr. Mapped Reads (rCRS)** | **Nr. Mapped Reads Passed Post-Filter** | **5 Prime C>T 1st base** | **5 Prime C>T 2nd base** | **Mean Length Mapped Reads** | **Nr. Dedup. Mapped Reads** | **Mean cov** | **% GC Dedup. Mapped Reads** | **Endogenous DNA (%)** | **Endogenous DNA Post (%)** |
| --- | --- | --- | --- | --- | --- | --- | --- | --- | --- | --- | --- |
| **C10_S149** | 1064187 | 36 | 35 | 0.0% | 0.0% | 76.45bp | 6 | 0.0X | 46% | 0 | 0 |
| **C20_S150** | 975752 | 38 | 4 | 100.0% | 0.0% | 36.00bp | 1 | 0.0X | 48% | 0 | 0 |
| **C30_S151** | 703394 | 32 | 26 | 0.0% | 0.0% | 74.00bp | 2 | 0.0X | 52% | 0 | 0 |
| **C40_S152** | 1352551 | 537 | 277 | 30.4% | 18.2% | 54.84bp | 95 | 0.3X | 46% | 0.04 | 0.02 |
| **C50_S153** | 1108096 | 38 | 9 | 0.0% | 0.0% | 98.33bp | 3 | 0.0X | 46% | 0 | 0 |
| **C60_S154** | 1029023 | 86 | 19 | 50.0% | 0.0% | 46.00bp | 3 | 0.0X | 43% | 0.01 | 0 |
| **C70_S155** | 1114854 | 29 | 2 | 0.0% | 0.0% | 44.00bp | 1 | 0.0X | 57% | 0 | 0 |
| **CL80_S156** | 908294 | 0 | 0 | n/a | n/a | n/a | n/a | n/a | n/a | 0 | 0 |
| **CL90_S157** | 18736 | 1 | 1 | 0.0% | 0.0% | 66.00bp | 1 | 0.0X | 47% | 0.01 | 0.01 |
| **CLBK1_S158** | 8276 | 111 | 111 | 0.0% | 0.0% | 82.92bp | 13 | 0.1X | 44% | 1.34 | 1.34 |
| ****n/a = no data available**** |  |  |  |  |  |  |  |  |  |  |  |

**Table S3 EAGER summary statistics for human mapped enriched libraries batch 2.**

| **Sample Name** | **Nr. Reads Into Mapping** | **Nr. Mapped Reads (rCRS)** | **Nr. Mapped Reads Passed Post-Filter** | **5 Prime C>T 1st base** | **5 Prime C>T 2nd base** | **Mean Length Mapped Reads** | **Nr. Dedup. Mapped Reads** | **Mean cov** | **% GC Dedup. Mapped Reads** | **Endogenous DNA (%)** | **Endogenous DNA Post (%)** |
| --- | --- | --- | --- | --- | --- | --- | --- | --- | --- | --- | --- |
| **CLO-10-EAP_S124_L001** | 1880411 | 9155 | 9139 | 0.0% | 10.0% | 51.37bp | 39 | 0.1X | 48% | 0.49 | 0.49 |
| **CLO-12-EAP_S102_L001** | 2243754 | 6589 | 5799 | 0.0% | 0.0% | 55.12bp | 13 | 0.0X | 46% | 0.29 | 0.26 |
| **CLO-15-EAP_S103_L001** | 1132514 | 22008 | 21176 | 0.0% | 0.0% | 50.31bp | 24 | 0.1X | 48% | 1.94 | 1.87 |
| **CLO-17-EAP_S104_L001** | 2107333 | 859 | 692 | 0.0% | 0.0% | 59.31bp | 13 | 0.0X | 48% | 0.04 | 0.03 |
| **CLO-20-EAP_S125_L001** | 1508867 | 1135 | 443 | 0.0% | 0.0% | 51.45bp | 12 | 0.0X | 53% | 0.08 | 0.03 |
| **CLO-25-EAP_S105_L001** | 2095996 | 123 | 2 | 0.0% | 0.0% | 42.00bp | 1 | 0.0X | 48% | 0.01 | 0 |
| **CLO-30-EAP_S126_L001** | 2715291 | 7 | 0 | n/a | n/a | n/a | n/a | n/a | n/a | 0 | 0 |
| **CLO-35-EAP_S107_L001** | 2590460 | 1437 | 762 | 0.0% | 0.0% | 54.84bp | 27 | 0.1X | 47% | 0.06 | 0.03 |
| **CLO-38-EAP_S108_L001** | 1939778 | 8526 | 8265 | 0.0% | 0.0% | 52.84bp | 37 | 0.1X | 44% | 0.44 | 0.43 |
| **CLO-42-EAP_S109_L001** | 2506952 | 8395 | 5346 | 36.4% | 16.7% | 48.27bp | 50 | 0.1X | 49% | 0.33 | 0.21 |
| **CLO-45-EAP_S110_L001** | 2736282 | 5018 | 3749 | 7.7% | 14.3% | 51.91bp | 55 | 0.2X | 49% | 0.18 | 0.14 |
| **CLO-47-EAP_S111_L001** | 2897163 | 1307 | 556 | 25.0% | 33.3% | 50.85bp | 12 | 0.0X | 52% | 0.05 | 0.02 |
| **CLO-5-EAP_S100_L001** | 2260541 | 3779 | 3775 | 0.0% | 0.0% | 44.86bp | 7 | 0.0X | 52% | 0.17 | 0.17 |
| **CLO-50-EAP_S127_L001** | 1911109 | 10340 | 7880 | 0.0% | 0.0% | 46.64bp | 11 | 0.0X | 50% | 0.54 | 0.41 |
| **CLO-52-EAP_S112_L001** | 2783712 | 8240 | 5155 | 0.0% | 0.0% | 56.46bp | 18 | 0.1X | 47% | 0.3 | 0.19 |
| **CLO-55-EAP_S113_L001** | 2717626 | 4972 | 1742 | 0.0% | 0.0% | 56.72bp | 22 | 0.1X | 49% | 0.18 | 0.06 |
| **CLO-57-EAP_S114_L001** | 1844545 | 7144 | 3934 | 38.1% | 20.0% | 48.26bp | 95 | 0.3X | 47% | 0.39 | 0.21 |
| **CLO-62-EAP_S115_L001** | 2765993 | 190 | 132 | 100.0% | 0.0% | 41.00bp | 1 | 0.0X | 52% | 0.01 | 0 |
| **CLO-65-EAP_S116_L001** | 3222064 | 3739 | 1461 | 0.0% | 0.0% | 57.10bp | 17 | 0.1X | 45% | 0.12 | 0.05 |
| **CLO-70-EAP_S128_L001** | 2163960 | 316 | 272 | 0.0% | 0.0% | 56.00bp | 8 | 0.0X | 48% | 0.01 | 0.01 |
| **CLO-75-EAP_S117_L001** | 2491596 | 763 | 522 | 0.0% | 0.0% | 61.45bp | 12 | 0.0X | 49% | 0.03 | 0.02 |
| **CLO-8-EAP_S101_L001** | 1674423 | 23774 | 23681 | 0.0% | 0.0% | 63.86bp | 24 | 0.1X | 42% | 1.42 | 1.41 |
| **CLO-80-EAP_S118_L001** | 1400720 | 0 | 0 | n/a | n/a | n/a | n/a | n/a | n/a | 0 | 0 |
| **CLO-82-EAP_S119_L001** | 1785279 | 210 | 5 | 0.0% | 0.0% | 61.00bp | 1 | 0.0X | 58% | 0.01 | 0 |
| **CLO-85-EAP_S120_L001** | 2595466 | 1 | 0 | n/a | n/a | n/a | n/a | n/a | n/a | 0 | 0 |
| **CLO-90-EAP_S121_L001** | 2424078 | 0 | 0 | n/a | n/a | n/a | n/a | n/a | n/a | 0 | 0 |
| **CLO-BK1-EAP_S122_L001** | 197608 | 56267 | 56119 | 0.0% | 13.3% | 58.75bp | 87 | 0.3X | 46% | 28.47 | 28.4 |
| **CLO-BK2-EAP_S106_L001** | 308182 | 18342 | 18337 | 0.0% | 0.0% | 53.44bp | 9 | 0.0X | 41% | 5.95 | 5.95 |
| **CLO-BK3-EAP_S123_L001** | 27185 | 14759 | 14703 | 0.0% | 0.0% | 55.59bp | 33 | 0.1X | 47% | 54.29 | 54.08 |
| **LiB-BK2-EAP_S129_L001** | 15423 | 10660 | 10653 | 0.0% | 0.0% | 48.88bp | 9 | 0.0X | 52% | 69.12 | 69.07 |
| ****n/a = no data available**** |  |  |  |  |  |  |  |  |  |  |  |

**Table S4 Chloroplast gene coordinates for 6 plant taxa.**

| **Species** | **rbcL** | **matK** | **trnL-UAA** |
| --- | --- | --- | --- |
| Zea | 56873-58304 | 1673-3215 | 48965-49508 |
| Artemisia | 54743-56201 | 2073-3591 | 46578-47087 |
| Cucurbita | 58695-60144 | 2007-3519 | 50071-50624 |
| Helianthus | 54552-56010 | 2064-3567 | 47024-47546 |
| Plantago | 53554-54988 | 1917-3426 | 45864-46457 |
| Phaseolus | 8092-9523 | 4963-6505 | 16308-16917 |

**Table S5** Age-depth model data for Fig. S14.

| **^14^C-Llew-Williams et al. 2024** | | | |  |
| --- | --- | --- | --- | --- |
| Lab ID | Age | Uncertainty | Depth (cm) | Corresponding Age-depth model ID |
| UOC-19148 | 245 | 14 | 37 | *R_Date 1* |
| UOC-19149 | 294 | 14 | 38 | *R_Date 2* |
| UOC-19151 | 400 | 14 | 52 | *R_Date 3* |
| UOC-19152 | 707 | 15 | 64 | *R_Date 4* |
| UOC-19153 | 1110 | 15 | 78 | *R_Date 5* |
| UOC-19154 | 1282 | 15 | 83 | *R_Date 6* |
| UOC-19155 | 1327 | 15 | 84 | *R_Date 7* |
|  |  |  |  |  |
| **^210^Pb-McCarthy et al. 2023** | | | | |
| Cumul.\|Drymass | Midpoint\|Depth | Year\|(CRS) | Age\|(CRS) | Error\|(CRS) |
| [g/cm^2^] | [cm] | [y] | [y] | [y] |
| (cumuldrymass) | (midpointdepth) | (crsyear) | (crsage) | (crserr) |
| 0 | 0 | 2019.14 | 0 | 0 |
| 0.0826 | 0.5 | 2013.94 | 5.2 | 0.45 |
| 0.1964 | 1.5 | 2005.77 | 13.36 | 1.05 |
| 0.2587 | 3.5 | 2000.65 | 18.49 | 1.29 |
| 0.3264 | 5.5 | 1995.46 | 23.68 | 1.57 |
| 0.4163 | 6.5 | 1989.82 | 29.31 | 1.92 |
| 0.5172 | 7.5 | 1983.75 | 35.38 | 2.34 |
| 0.6125 | 8.5 | 1975.31 | 43.82 | 3.05 |
| 0.7078 | 9.5 | 1966.97 | 52.17 | 3.97 |
| 0.803 | 10.5 | 1958 | 61.14 | 5.14 |
| 0.9097 | 11.5 | 1945.66 | 73.48 |  |
|  |  |  |  | 7.55 |
| 1.0163 | 12.5 | 1932.57 | 86.57 | 11.23 |
| 1.1005 | 14.5 | 1922.69 | 96.45 | 14.68 |
| 1.1904 | 15.5 | 1914.46 | 104.68 | 18.52 |
| 1.2913 | 16.5 | 1902.48 | 116.66 | 25.09 |
| 1.3866 | 17.5 | 1894.36 | 124.78 | 30.93 |
| 1.4818 | 18.5 | 1893.12 | 126.02 | 29.24 |
| 1.5943 | 19.5 | 1891.47 | 127.67 | 27.81 |
| 1.7011 | 20.5 | 1886.18 | 132.96 | 25.42 |
| 1.7853 | 21.5 | 1875.72 | 143.41 | 27.43 |
| 1.875 | 22.5 | 1845.64 | 173.5 | 53.07 |
| 1.9816 | 23.5 | 1798.31 | 220.83 | 114.56 |

**Table S6** Age estimate by depth (z) for core CL-19.

| **intcal20_t** | **intcal20_dt** | **z (cm)** | **median** | **sed** | **From (2σ)** | **To (2σ)** |
| --- | --- | --- | --- | --- | --- | --- |
| 2019.1 | 0 | 0 | 2019 | 0.0962 | 2018 | 2019 |
| 2009.9 | 0 | 1 | 2009 | 0.147 | 2009 | 2010 |
| 2004.5 | 0 | 2 | 2004 | 0.237 | 2004 | 2005 |
| 2001.9 | 0 | 3 | 2001 | 0.338 | 2001 | 2002 |
| 1999.4 | 0 | 4 | 1999 | 0.352 | 1999 | 2000 |
| 1996.8 | 0 | 5 | 1996 | 0.279 | 1996 | 1997 |
| 1992.6 | 0 | 6 | 1992 | 0.207 | 1992 | 1993 |
| 1986.8 | 0 | 7 | 1986 | 0.154 | 1986 | 1987 |
| 1979.5 | 0 | 8 | 1979 | 0.129 | 1979 | 1980 |
| 1971.1 | 0 | 9 | 1971 | 0.117 | 1970 | 1971 |
| 1962.5 | 0 | 10 | 1962 | 0.105 | 1962 | 1963 |
| 1951.8 | 0 | 11 | 1951 | 0.0862 | 1951 | 1952 |
| 1939.1 | 0 | 12 | 1939 | 0.0948 | 1939 | 1940 |
| 1930.1 | 0 | 13 | 1930 | 0.121 | 1930 | 1931 |
| 1925.2 | 0 | 14 | 1925 | 0.142 | 1925 | 1926 |
| 1918.6 | 0 | 15 | 1918 | 0.125 | 1918 | 1919 |
| 1908.5 | 0 | 16 | 1908 | 0.0992 | 1907 | 1908 |
| 1898.4 | 0 | 17 | 1898 | 0.157 | 1897 | 1898 |
| 1893.7 | 0 | 18 | 1893 | 0.453 | 1893 | 1894 |
| 1892.3 | 0 | 19 | 1892 | 0.49 | 1891 | 1892 |
| 1888.8 | 0 | 20 | 1888 | 0.208 | 1888 | 1889 |
| 1881 | 0 | 21 | 1880 | 0.0882 | 1880 | 1881 |
| 1860.7 | 0 | 22 | 1860 | 0.0376 | 1860 | 1861 |
| 1822 | 0 | 23 | 1821 | 0.0301 | 1821 | 1822 |
| 1792.9 | 4.1 | 24 | 1793 | 0.0514 | 1783 | 1799 |
| 1782 | 12.4 | 25 | 1784 | 0.0855 | 1754 | 1800 |
| 1771.1 | 16.1 | 26 | 1773 | 0.0919 | 1736 | 1797 |
| 1760.2 | 18.5 | 27 | 1762 | 0.0919 | 1721 | 1793 |
| 1749.4 | 19.8 | 28 | 1751 | 0.0919 | 1709 | 1786 |
| 1738.5 | 20.7 | 29 | 1739 | 0.0919 | 1697 | 1778 |
| 1727.6 | 21.2 | 30 | 1727 | 0.0919 | 1685 | 1769 |
| 1716.7 | 21 | 31 | 1716 | 0.0919 | 1675 | 1759 |
| 1705.9 | 20.7 | 32 | 1704 | 0.092 | 1666 | 1749 |
| 1695 | 19.5 | 33 | 1693 | 0.0919 | 1658 | 1735 |
| 1684.1 | 17.9 | 34 | 1682 | 0.0919 | 1652 | 1720 |
| 1673.2 | 15.5 | 35 | 1671 | 0.0919 | 1647 | 1705 |
| 1662.3 | 11 | 36 | 1660 | 0.0897 | 1643 | 1685 |
| 1651.5 | 5.6 | 37 | 1651 | 0.0864 | 1640 | 1664 |
| 1639.2 | 6.7 | 38 | 1639 | 0.0811 | 1626 | 1652 |
| 1626.8 | 12.8 | 39 | 1628 | 0.0808 | 1599 | 1648 |
| 1614.4 | 18 | 40 | 1617 | 0.0805 | 1575 | 1644 |
| 1602 | 20.7 | 41 | 1604 | 0.0806 | 1558 | 1638 |
| 1589.5 | 22.7 | 42 | 1591 | 0.0806 | 1543 | 1631 |
| 1577.1 | 24.1 | 43 | 1578 | 0.0806 | 1529 | 1622 |
| 1564.7 | 24.8 | 44 | 1565 | 0.0806 | 1515 | 1612 |
| 1552.3 | 25.3 | 45 | 1552 | 0.0805 | 1502 | 1602 |
| 1539.9 | 25 | 46 | 1539 | 0.0805 | 1491 | 1590 |
| 1527.5 | 24.4 | 47 | 1526 | 0.0805 | 1480 | 1577 |
| 1515 | 23.2 | 48 | 1513 | 0.0805 | 1471 | 1563 |
| 1502.6 | 21.4 | 49 | 1500 | 0.0806 | 1463 | 1547 |
| 1490.2 | 19.1 | 50 | 1487 | 0.0806 | 1457 | 1529 |
| 1477.8 | 14.8 | 51 | 1475 | 0.0772 | 1451 | 1509 |
| 1465.4 | 10 | 52 | 1464 | 0.0727 | 1446 | 1488 |
| 1450.3 | 16.7 | 53 | 1451 | 0.0685 | 1413 | 1483 |
| 1435.3 | 21.7 | 54 | 1437 | 0.0664 | 1387 | 1475 |
| 1420.2 | 24.9 | 55 | 1422 | 0.0664 | 1368 | 1466 |
| 1405.1 | 26.5 | 56 | 1406 | 0.0664 | 1350 | 1455 |
| 1390 | 27.6 | 57 | 1390 | 0.0664 | 1334 | 1443 |
| 1375 | 28.1 | 58 | 1375 | 0.0664 | 1320 | 1430 |
| 1359.9 | 27.4 | 59 | 1359 | 0.0664 | 1307 | 1415 |
| 1344.9 | 26.2 | 60 | 1343 | 0.0664 | 1295 | 1398 |
| 1329.8 | 24.4 | 61 | 1327 | 0.0664 | 1286 | 1381 |
| 1314.8 | 20.8 | 62 | 1311 | 0.0665 | 1280 | 1360 |
| 1299.7 | 14.9 | 63 | 1297 | 0.0608 | 1276 | 1332 |
| 1284.7 | 6.7 | 64 | 1284 | 0.0496 | 1273 | 1298 |
| 1259.4 | 22.2 | 65 | 1263 | 0.0438 | 1214 | 1293 |
| 1234.1 | 35.3 | 66 | 1240 | 0.0395 | 1160 | 1288 |
| 1208.7 | 41.3 | 67 | 1214 | 0.0395 | 1123 | 1279 |
| 1183.4 | 45.9 | 68 | 1188 | 0.0395 | 1089 | 1266 |
| 1158 | 49.1 | 69 | 1161 | 0.0394 | 1059 | 1250 |
| 1132.7 | 50.9 | 70 | 1134 | 0.0394 | 1030 | 1231 |
| 1107.3 | 52.2 | 71 | 1108 | 0.0394 | 1002 | 1210 |
| 1081.9 | 52 | 72 | 1081 | 0.0394 | 979 | 1185 |
| 1056.5 | 51.2 | 73 | 1054 | 0.0394 | 956 | 1159 |
| 1031.2 | 49.3 | 74 | 1028 | 0.0394 | 936 | 1130 |
| 1005.8 | 46.3 | 75 | 1002 | 0.0395 | 919 | 1098 |
| 980.5 | 42.3 | 76 | 977 | 0.0395 | 905 | 1065 |
| 955.1 | 35.7 | 77 | 949 | 0.0366 | 896 | 1026 |
| 929.8 | 28.3 | 78 | 921 | 0.0329 | 890 | 985 |
| 894.3 | 38.8 | 79 | 893 | 0.0291 | 820 | 973 |
| 858.7 | 43.6 | 80 | 859 | 0.0281 | 774 | 951 |
| 823.1 | 43.4 | 81 | 821 | 0.0281 | 732 | 916 |
| 787.5 | 38.2 | 82 | 786 | 0.0305 | 695 | 861 |
| 751.9 | 26.8 | 83 | 761 | 0.0402 | 673 | 779 |
| 737.8 | 33.6 | 84 | 752 | 0.0708 | 659 | 773 |
| 722.6 | 33.6 | 85 | 737 | 0.0656 | 643 | 758 |
| 707.3 | 33.6 | 86 | 722 | 0.0656 | 627 | 743 |
| 692.1 | 33.6 | 87 | 707 | 0.0656 | 610 | 729 |
| 676.8 | 33.7 | 88 | 692 | 0.0656 | 594 | 714 |
| 661.6 | 33.7 | 89 | 677 | 0.0656 | 578 | 699 |
| 646.3 | 33.7 | 90 | 662 | 0.0656 | 562 | 684 |

**Table S7 CONISS zonation summary for Crawford Lake sedaDNA assemblage.**

|  | **CONISS Zonation (4-zone solution)** | | | | **Broken-stick (statistically significant) zonation** | |  |
| --- | --- | --- | --- | --- | --- | --- | --- |
| **Depth (cm)** | **On-target (plants & animals)** | **Off-target (microbes, fungi & algae)** | **Combined (½-weighted: on + off)** | **Agreement (3 headline analyses)** | **On-target  (bstick)** | **Off-target (bstick)** | **Interpretation  (sedaDNA + other proxies)** |
| **5** | **I** | **I** | **I** | **Agree** | **I** | **I** | Zone 1  Euro-Canadian /  “Anthropocene” |
| **8** | **I** | **I** | **I** | **Agree** | **I** | **I** |  |
| **10** | **I** | **I** | **I** | **Agree** | **I** | **I** |  |
| **12** | **I** | **I** | **I** | **Agree** | **I** | **I** |  |
| **15** | **I** | **II** | **I** | **Boundary offset** | **I** | **I** | Zone 1  Euro-Canadian |
| **17** | **II** | **II** | **II** | **Agree** | **I** | **I** |  |
| **20** | **II** | **II** | **II** | **Agree** | **I** | **I** |  |
| **25** | **II** | **III** | **II** | **Boundary offset** | **I** | **II** | Zone 2  Post-farming |
| **30** | **II** | **III** | **II** | **Boundary offset** | **I** | **II** |  |
| **35** | **II** | **III** | **II** | **Boundary offset** | **I** | **II** |  |
| **38** | **III** | **III** | **III** | **Agree** | **I** | **II** | Zone 2  Indigenous Farming |
| **40** | **III** | **III** | **III** | **Agree** | **I** | **II** |  |
| **42** | **III** | **III** | **III** | **Agree** | **I** | **II** |  |
| **45** | **III** | **III** | **III** | **Agree** | **I** | **II** |  |
| **47** | **III** | **III** | **III** | **Agree** | **I** | **II** |  |
| **50** | **III** | **III** | **III** | **Agree** | **I** | **II** |  |
| **52** | **III** | **III** | **III** | **Agree** | **I** | **II** |  |
| **55** | **III** | **III** | **III** | **Agree** | **I** | **II** |  |
| **57** | **III** | **III** | **III** | **Agree** | **I** | **II** |  |
| **60** | **III** | **III** | **III** | **Agree** | **I** | **II** |  |
| **62** | **III** | **IV** | **III** | **Boundary offset** | **I** | **II** |  |
| **65** | **IV** | **IV** | **IV** | **Agree** | **II** | **II** | Zone 3  Pre-farming |
| **70** | **IV** | **IV** | **IV** | **Agree** | **II** | **II** |  |
| **75** | **IV** | **IV** | **IV** | **Agree** | **II** | **II** |  |
| **80** | **IV** | **IV** | **IV** | **Agree** | **II** | **II** |  |
| **82** | **IV** | **IV** | **IV** | **Agree** | **II** | **II** |  |
| **85** | **IV** | **IV** | **IV** | **Agree** | **II** | **II** |  |
| **88** | **IV** | **IV** | **IV** | **Agree** | **II** | **II** |  |

Per-depth zone assignments from constrained incremental sum of squares (CONISS) (Grimm, 1987) cluster analysis applied to 28 depth intervals (5–88 cm), zone_bstick (Bennett, 1996): two-zone broken-stick solution with offset between on-target and off-target taxa, suggesting 3 primary ecological zones within the upper 88 cm at Crawford Lake based on sedaDNA. See Figures S35-S37 for dendrogram and stratigraphy plots.

**Table S8 NCBI-NT reference database coverage for key interpreted taxa at time of analysis (2020) versus current (June 2026).** Pre-2021 sequences: count of sequences with accession date before 2021-01-01 in NCBI-NT. Post-2020 sequences: new sequences added after 2020.

| **Taxon** | **Rank** | **Group** | **Pre-Jan2021 sequences** | **New post-Jan2021 sequences** | **% post- 2020** | **Assignment robustness** | **Notes** |
| --- | --- | --- | --- | --- | --- | --- | --- |
| Botryococcus braunii | species | algae | 215,058 | 111 | 0.1% | High | Algal biomarker; comprehensively covered in 2020 |
| Chlorella | genus | algae | 110,399 | 1,311 | 1.2% | High | Green alga; essentially complete in 2020 |
| Zea mays | species | plants | 4,878,782 | 10,670 | 0.2% | High | Genome well-established; assignment robust |
| Phaseolus vulgaris | species | plants | 828,028 | 62,471 | 7.0% | High | Common bean; well represented in 2020 |
| Legionella | genus | bacteria | 117,021 | 45,413 | 28.0% | High | Well covered in 2020; 117k pre-2020 sequences |
| Schizophyllum commune | species | fungi | 42,999 | 16,884 | 28.2% | Moderate | Adequate 2020 coverage; see Tentative tier in Table S8 |
| Microvirga | genus | bacteria | 13,093 | 7,772 | 37.2% | Moderate | Well represented for genus-level LCA in 2020 |
| Cyanobacteria | phylum | cyanobacteria | 538,375 | 1,081,684 | 66.8% | High | Post-2020 growth dominated by MAGs; phylum-level unaffected |
| Planktothrix agardhii | species | cyanobacteria | 2,686 | 6,176 | 69.7% | Moderate | Significant growth but genus-level ID robust with 2,686 pre-2020 seqs |
| Synechococcus | genus | cyanobacteria | 36,054 | 97,541 | 73.0% | Moderate | 36k pre-2020 sequences sufficient for genus-level LCA; growth from MAGs |
| Planktothrix rubescens | species | cyanobacteria | 2,566 | 11,720 | 82.0% | Moderate | Adequate 2020 representation for genus-level LCA |
| Polynucleobacter | genus | bacteria | 10,950 | 74,581 | 87.2% | Moderate | Largest growth; mostly environmental MAGs; 10k pre-2020 seqs sufficient |
| Pelolinea | genus | bacteria | 38 | 46 | 54.8% | Low | Sparsely represented in both databases (84 total); assignment confidence limited |

**Table S9 Calculated BLAST floor.** Minimum read length at which BLAST reports any alignment at all, and that alignment's bit score reaches MEGAN's *MinScore* = 50 threshold, by BLAST task, mismatch placement scenario, and target percent identity:

| BLAST task | Mismatch placement | Target % identity | Min length  any hit | Min length passes MinScore=50 |
| --- | --- | --- | --- | --- |
| blastn | ends (realistic, ancient-DNA-like) | 100 | 18 bp | 27 bp |
| blastn | ends | 99 | 18 bp | 27 bp |
| blastn | ends | 98 | 18 bp | 28 bp |
| blastn | ends | 97 | 18 bp | 28 bp |
| blastn | ends | 96 | 18 bp | 28 bp |
| blastn | ends | 95 | 18 bp | 28 bp |
| blastn | even (worst case) | 100 | 18 bp | 27 bp |
| blastn | even | 99 | 18 bp | 27 bp |
| blastn | even | 98 | 18 bp | 30 bp |
| blastn | even | 97 | 22 bp | 30 bp |
| blastn | even | 96 | 22 bp | 30 bp |
| blastn | even | 95 | 22 bp | 32 bp |
| megablast | ends (realistic, ancient-DNA-like) | 100 | 28 bp | 28 bp |
| megablast | ends | 99 | 28 bp | 28 bp |
| megablast | ends | 98 | 29 bp | 29 bp |
| megablast | ends | 97 | 29 bp | 29 bp |
| megablast | ends | 96 | 29 bp | 29 bp |
| megablast | ends | 95 | 29 bp | 29 bp |
| megablast | even (worst case) | 100 | 28 bp | 28 bp |
| megablast | even | 99 | 28 bp | 28 bp |
| megablast | even | 98 | 56 bp | 56 bp |
| megablast | even | 97 | 82 bp | 82 bp |
| megablast | even | 96 | *no hit within 120 bp test range* | *no hit within 120 bp test range* |
| megablast | even | 95 | *no hit within 120 bp test range* | *no hit within 120 bp test range* |

**Table S10 Branta canadensis mtDNA vs nuclear, shotgun vs capture**. Unique reads, MQ≥30. Includes 0-round (shotgun), 1-round (C#/CL#) and 2-round (CLO-#) capture. Nuclear reads are diverse (low duplication), short, and low-identity.

| **Library** | **Depth (cm)** | **Type** | **Total**  **reads (M)** | **mtDNA unique** | **mtDNA %NM≤1** | **mtDNA dup%** | **nuclear unique** | **nuclear %NM≤1** | **nuclear dup%** | **nuclear mean len (bp)** |
| --- | --- | --- | --- | --- | --- | --- | --- | --- | --- | --- |
| CLO-5-EAP | 5 | capture (2 round) | 2.37 | 10 | 10 | 95.6 | 2398 | 2.1 | 75.4 | 27.2 |
| CLO-8-EAP | 8 | capture (2 round) | 1.98 | 7 | 14.3 | 70.8 | 20396 | 1.6 | 40.2 | 27.2 |
| CLO-10-EAP | 10 | capture (2 round) | 1.92 | 0 | — | — | 3357 | 7.2 | 55.8 | 40.9 |
| CLO-12-EAP | 12 | capture (2 round) | 2.26 | 4 | 25 | 84.6 | 1205 | 6.1 | 65.2 | 32.6 |
| CLO-15-EAP | 15 | capture (2 round) | 1.33 | 7 | 0 | 99.9 | 4044 | 9.1 | 78.9 | 45.4 |
| CLO-17-EAP | 17 | capture (2 round) | 2.19 | 33 | 15.2 | 90 | 5379 | 2.8 | 35.3 | 28.5 |
| CLO-20-EAP | 20 | capture (2 round) | 1.58 | 21 | 23.8 | 97 | 2591 | 2.1 | 41.7 | 27.8 |
| CLO-25-EAP | 25 | capture (2 round) | 2.12 | 14 | 7.1 | 93.7 | 1094 | 3.6 | 64.4 | 35 |
| CLO-30-EAP | 30 | capture (2 round) | 2.75 | 13 | 30.8 | 97 | 1799 | 2.6 | 90.2 | 30.4 |
| CLO-35-EAP | 35 | capture (2 round) | 2.62 | 36 | 22.2 | 94.2 | 3235 | 8 | 47.7 | 38.1 |
| CLO-38-EAP | 38 | capture (2 round) | 2 | 29 | 69 | 99.1 | 3132 | 1.7 | 48.2 | 28.5 |
| CLO-42-EAP | 42 | capture (2 round) | 2.51 | 4683 | 87.2 | 96.1 | 2306 | 13.7 | 68.3 | 37.2 |
| CLO-45-EAP | 45 | capture (2 round) | 2.73 | 7048 | 90.4 | 96.3 | 1469 | 28.5 | 84 | 47.3 |
| CLO-47-EAP | 47 | capture (2 round) | 2.89 | 2502 | 87 | 98.4 | 1459 | 17.5 | 83.4 | 45.6 |
| CLO-50-EAP | 50 | capture (2 round) | 2.08 | 495 | 83 | 99.4 | 5675 | 3.2 | 66 | 28.5 |
| CLO-52-EAP | 52 | capture (2 round) | 2.78 | 2164 | 87.9 | 98.7 | 1252 | 10.6 | 86.6 | 36.5 |
| CLO-55-EAP | 55 | capture (2 round) | 2.75 | 1286 | 88.6 | 98.1 | 1334 | 9.2 | 83.1 | 34.1 |
| CLO-57-EAP | 57 | capture (2 round) | 1.84 | 9136 | 85.3 | 91.3 | 2018 | 24.4 | 70.5 | 41.4 |
| CLO-62-EAP | 62 | capture (2 round) | 2.88 | 125 | 75.2 | 99.2 | 10185 | 1.6 | 36.4 | 27.5 |
| CLO-65-EAP | 65 | capture (2 round) | 3.24 | 44 | 84.1 | 99.3 | 1702 | 1.5 | 51.7 | 29.3 |
| CLO-70-EAP | 70 | capture (2 round) | 2.19 | 19 | 31.6 | 99.1 | 1065 | 1.8 | 56 | 31.7 |
| CLO-75-EAP | 75 | capture (2 round) | 2.53 | 15 | 13.3 | 92 | 3443 | 1.2 | 40.6 | 27.3 |
| CLO-80-EAP | 80 | capture (2 round) | 1.44 | 4 | 25 | 89.2 | 175 | 1.7 | 72 | 33.2 |
| CLO-82-EAP | 82 | capture (2 round) | 1.77 | 37 | 67.6 | 99 | 1811 | 1.1 | 54.5 | 28.4 |
| CLO-85-EAP | 85 | capture (2 round) | 2.49 | 4 | 25 | 96.4 | 516 | 3.1 | 78.7 | 33.9 |
| CLO-90-EAP | 88 | capture (2 round) | 2.38 | 2 | 0 | 99 | 462 | 1.9 | 90.3 | 36.5 |
| *CLO-BK1-EAP* |  | *capture (2 round)* | *0.2* | *1* | *0* | *98.1* | *2* | *0* | *0* | *51* |
| *CLO-BK2-EAP* |  | *capture (2 round)* | *0.31* | *3* | *0* | *100* | *4* | *0* | *20* | *46.5* |
| *CLO-BK3-EAP* |  | *capture (2 round)* | *0.03* | *0* | *—* | *—* | *12* | *0* | *20* | *31.8* |
| *LiB-BK2-EAP* |  | *capture (2 round)* | *0.02* | *0* | *—* | *—* | *4* | *0* | *33.3* | *36* |
| CLO-5-Shg | 5 | shotgun | 5.92 | 7 | 0 | 0 | 279150 | 0.3 | 7 | 25.1 |
| CLO-20-Shg | 20 | shotgun | 7.35 | 17 | 5.9 | 5.6 | 234634 | 0.4 | 4.3 | 25.3 |
| CLO-35-Shg | 35 | shotgun | 7.27 | 9 | 0 | 0 | 103868 | 0.7 | 4.7 | 25.9 |
| CLO-40-Shg | 40 | shotgun | 5.12 | 107 | 90.7 | 2.7 | 37021 | 26.5 | 4.9 | 31.9 |
| CLO-42-Shg | 42 | shotgun | 6.15 | 21 | 81 | 0 | 47770 | 3.9 | 4.4 | 26.8 |
| CLO-45-Shg | 45 | shotgun | 4.47 | 16 | 87.5 | 0 | 15754 | 7.2 | 5.5 | 28.9 |
| CLO-57-Shg | 57 | shotgun | 4.73 | 15 | 93.3 | 11.8 | 41291 | 7.7 | 4.9 | 27.3 |
| CLO-60-Shg | 60 | shotgun | 8.92 | 25 | 48 | 13.8 | 128989 | 1.9 | 5.5 | 25.9 |
| CLO-75-Shg | 75 | shotgun | 5.98 | 9 | 0 | 10 | 150803 | 0.6 | 4.3 | 25.6 |
| *CLO-BK1-Shg* |  | *shotgun* | *2.01* | *2* | *50* | *66.7* | *10891* | *0.5* | *57.1* | *25.6* |
| *LiB-BK-Shg* |  | *shotgun* | *0* | *0* | *—* | *—* | *0* | *—* | *—* | *—* |
| C10 | 10 | capture (1 round) | 1.06 | 2 | 0 | 50 | 5235 | 8.8 | 23.6 | 46.2 |
| C20 | 20 | capture (1 round) | 0.98 | 3 | 0 | 0 | 1939 | 6.7 | 16.3 | 34.8 |
| C30 | 30 | capture (1 round) | 0.7 | 6 | 16.7 | 92.9 | 829 | 6 | 29 | 36.6 |
| C40 | 40 | capture (1 round) | 1.35 | 15028 | 83.3 | 41.9 | 4966 | 61.8 | 19 | 46.9 |
| C50 | 50 | capture (1 round) | 1.11 | 323 | 91 | 69.9 | 1737 | 18.5 | 17.9 | 35 |
| C60 | 60 | capture (1 round) | 1.03 | 267 | 89.9 | 81.7 | 1767 | 13.1 | 24.5 | 36 |
| C70 | 70 | capture (1 round) | 1.11 | 9 | 33.3 | 90.8 | 1243 | 4.3 | 24.9 | 36.3 |
| CL80 | 80 | capture (1 round) | 0.91 | 0 | — | — | 575 | 4.5 | 5.7 | 34.9 |
| CL90 | 88 | capture (1 round) | 0.02 | 0 | — | — | 25 | 16 | 3.8 | 36.6 |
| *CLBK1* |  | *capture (1 round)* | *0.01* | *0* | *—* | *—* | *3* | *0* | *0* | *44* |

**Table S11 Nuclear vs mtDNA Branta competitive test.** Reads mapping to the *Branta* nuclear genome vs a distant-bird decoy (chicken, Gallus gallus GCF_016699485.2).

| **Metric (pooled sediment, unique reads MQ≥30)** | **Shotgun** | **Capture** |
| --- | --- | --- |
| Reads mapping to Branta nuclear | 1039280 | 83502 |
| Reads mapping to Chicken decoy | 927962 | 76125 |
| Chicken / Branta ratio | 0.89 | 0.91 |
| Goose-preferential (fewer mismatches to Branta), unique | 670890 | 56352 |
| Tie (equal mismatches), raw | 352775 | 54846 |
| Chicken-preferential (fewer to chicken), raw | 36945 | 8979 |
| Goose-preferential unique as ×(mtDNA unique signal) | ~2969× | — |
| Corr(goose-preferential, mtDNA) across shotgun libs | -0.36 | — |

If nuclear reads were Branta-specific, few would map to a distant bird; instead ~90% do, and the goose-preferential residual is still ~3,000× the mtDNA signal and anti-correlates with it. Implying much deeper sequencing is needed to reconstruct authentic nuclear signals.

**Supplementary Dataset Captions**

**Dataset S1. Characterization of the hybridization‑capture bait set.** Edit distance and percent sequence divergence for the 169,972 80‑bp capture baits used in this study, relative to their aligned references, across seven animal taxa (i.e., *Alces*, *Bison*, *Bos*, *Branta*, *Gavia*, *Mammuthus*, *Odocoileus*). The workbook contains an overall summary and per‑taxon summary of bait counts and divergence statistics, the full per‑bait table (i.e., edit distance, bait length, divergence, taxon), edit distance and divergence distributions, pairwise comparisons quantifying baits shared between taxa and their relative divergence, and individual bait tables for representative taxa.

**Dataset S2. Capture enrichment performance and bait conservation.** Comparison of shotgun versus hybridization‑enriched sequencing to quantify target enrichment. Includes overall enrichment statistics, such as per‑taxon (baitset; n = 756) and per‑reference (FullRefs; n = 3,543) shotgun coverage, enriched coverage, and fold enrichment; enrichment breakdowns by category (mutually exclusive and cumulative), shotgun‑coverage distribution, sequence‑conservation analyses of baits shared among taxon pairs (shared vs. unique divergence), and sample‑level read enrichment summaries (total and on‑target reads before vs. after capture).

**Dataset S3. Read‑level mapping and divergence of enriched sequences.** Edit distance, percent divergence, and read length for enriched reads mapped both to the bait set (Baitset; 1,402,036 reads across 757 taxa) and to the full reference database (FullRefs; 4,218,307 reads across 3,530 references). The workbook provides overall and per‑taxon/per‑reference summaries, edit‑distance, divergence, and cumulative distributions, the Figure 4 comparison of read versus bait divergence, and 100,000‑read subsamples of the underlying per‑read data. Roughly 99% of reads fall below 10% divergence.

**Dataset S4. Ensemble sedaDNA support using combined *BLASTn*/*MEGAN* and *Holi* classifications.** Both on- and off-target classifications are reported with full associated classification metadata from both approaches, as well as summary sheets showing condensed important metadata and classification support metrics.

**Supplementary References**

Bennett, K. D. (1996). Determination of the number of zones in a biostratigraphical sequence. *New Phytologist*, *132*(1), 155–170. https://doi.org/10.1111/J.1469-8137.1996.TB04521.X;PAGE:STRING:ARTICLE/CHAPTER

Dabney, J., Knapp, M., Glocke, I., Gansauge, M.-T., Weihmann, A., Nickel, B., Valdiosera, C., Garcia, N., Paabo, S., Arsuaga, J.-L., & Meyer, M. (2013). Complete mitochondrial genome sequence of a Middle Pleistocene cave bear reconstructed from ultrashort DNA fragments. *Proceedings of the National Academy of Sciences*, *110*(39), 15758–15763. https://doi.org/10.1073/pnas.1314445110

Edgar, R. C. (2004). MUSCLE: Multiple sequence alignment with high accuracy and high throughput. *Nucleic Acids Research*, *32*(5), 1792–1797. https://doi.org/10.1093/nar/gkh340

Ekdahl, E. J., Teranes, J. L., Guilderson, T. P., Turton, C. L., McAndrews, J. H., Wittkop, C. A., & Stoermer, E. F. (2004). Prehistorical record of cultural eutrophication from Crawford Lake, Canada. *Geology*, *32*(9), 745–748. https://doi.org/10.1130/G20496.1

Ewels, P., Magnusson, M., Lundin, S., & Käller, M. (2016). MultiQC: summarize analysis results for multiple tools and samples in a single report. *Bioinformatics*, *32*(19), 3047–3048. https://doi.org/10.1093/BIOINFORMATICS/BTW354

Gansauge, M. T., Aximu-Petri, A., Nagel, S., & Meyer, M. (2020). Manual and automated preparation of single-stranded DNA libraries for the sequencing of DNA from ancient biological remains and other sources of highly degraded DNA. *Nature Protocols*, *15*(8), 2279–2300. https://doi.org/10.1038/s41596-020-0338-0

Grimm, E. C. (1987). CONISS: a FORTRAN 77 program for stratigraphically constrained cluster analysis by the method of incremental sum of squares. *Computers & Geosciences*, *13*(1), 13–35. https://doi.org/10.1016/0098-3004(87)90022-7

Karpinski, E., Mead, J. I., & Poinar, H. N. (2016). Molecular identification of paleofeces from Bechan Cave, southeastern Utah, USA. *Quaternary International*, *443*, 140–146. https://doi.org/10.1016/j.quaint.2017.03.068

Kircher, M., Sawyer, S., & Meyer, M. (2012). Double indexing overcomes inaccuracies in multiplex sequencing on the Illumina platform. *Nucleic Acids Research*, *40*(1), 1–8. https://doi.org/10.1093/nar/gkr771

Li, H., & Durbin, R. (2009). Fast and accurate short read alignment with Burrows-Wheeler transform. *Bioinformatics (Oxford, England)*, *25*(14), 1754–1760. https://doi.org/10.1093/bioinformatics/btp324

Meyer, M., & Kircher, M. (2010). Illumina sequencing library preparation for highly multiplexed target capture and sequencing. *Cold Spring Harbor Protocols*, *5*(6). https://doi.org/10.1101/pdb.prot5448

Murchie, T. J., Cocker, S. L., Baleka, S., Vogel, N. A., Natola, L., Karpinski, E., Tirlea, D., Barrera, M. A., Grant, D. M., Morien, E., Long, G. S., Rutledge, L. Y., Zazula, G. D., Jensen, B. J., Froese, D. G., & Poinar, H. N. (2026). Ground squirrel coprolites preserve complex archives of ancient environmental DNA over 700,000 years. *Nature Communications*, *17*(1), 4868-. https://doi.org/10.1038/s41467-026-72977-6

Murchie, T. J., Karpinski, E., Eaton, K., Duggan, A. T., Baleka, S., Zazula, G., MacPhee, R. D. E., Froese, D., & Poinar, H. N. (2022). Pleistocene mitogenomes reconstructed from the environmental DNA of permafrost sediments. *Current Biology*, *32*(4), 851-860.e7. https://doi.org/10.1016/J.CUB.2021.12.023

Murchie, T. J., Kuch, M., Duggan, A. T., Ledger, M. L., Roche, K., Klunk, J., Karpinski, E., Hackenberger, D., Sadoway, T., MacPhee, R., Froese, D., & Poinar, H. (2021). Optimizing extraction and targeted capture of ancient environmental DNA for reconstructing past environments using the PalaeoChip Arctic-1.0 bait-set. *Quaternary Research*, *99*, 305–328. https://doi.org/10.1017/qua.2020.59

Murchie, T. J., Long, G. S., Lanoil, B. D., Froese, D., & Poinar, H. N. (2023). Permafrost microbial communities follow shifts in vegetation, soils, and megafauna extinctions in Late Pleistocene NW North America. *Environmental DNA*, *5*(6), 1759–1779. https://doi.org/10.1002/EDN3.493

Murchie, T. J., Monteath, A. J. A. J., Mahony, M. E., Long, G. S., Cocker, S., Sadoway, T., Karpinski, E., Zazula, G., MacPhee, R. D. E., Froese, D., Poinar, H. N., Cocker, S., Sadoway, T., Zazula, G., MacPhee, R. D. E., Froese, D., & Poinar, H. N. (2021). Collapse of the mammoth-steppe in central Yukon as revealed by ancient environmental DNA. *Nature Communications*, (1), 1–18. https://doi.org/10.1038/s41467-021-27439-6

Peltzer, A., Jäger, G., Herbig, A., Seitz, A., Kniep, C., Krause, J., & Nieselt, K. (2016). EAGER: efficient ancient genome reconstruction. *Genome Biology*, *17*(60). https://doi.org/10.1186/S13059-016-0918-Z/TABLES/10

Renaud, G., Stenzel, U., & Kelso, J. (2014). LeeHom: Adaptor trimming and merging for Illumina sequencing reads. *Nucleic Acids Research*, *42*(18), e141. https://doi.org/10.1093/nar/gku699

Rowe, A. G., Bataille, C. P., Baleka, S., Combs, E. A., Crass, B. A., Fisher, D. C., Ghosh, S., Holmes, C. E., Krasinski, K. E., Lanoë, F., Murchie, T. J., Poinar, H., Potter, B., Rasic, J. T., Reuther, J., Smith, G. M., Spaleta, K. J., Wygal, B. T., & Wooller, M. J. (2024). A female woolly mammoth’s lifetime movements end in an ancient Alaskan hunter-gatherer camp. *Science Advances*, *10*(3). https://doi.org/DOI: 10.1126/sciadv.adk0818

Schubert, M., Lindgreen, S., & Orlando, L. (2016). AdapterRemoval v2: Rapid adapter trimming, identification, and read merging. *BMC Research Notes*, *9*(88). https://doi.org/10.1186/S13104-016-1900-2/FIGURES/3
